# Advanced lesion symptom mapping analyses and implementation as *BCBtoolkit*

**DOI:** 10.1093/gigascience/giy004

**Published:** 2018-02-08

**Authors:** Chris Foulon, Leonardo Cerliani, Serge Kinkingnéhun, Richard Levy, Charlotte Rosso, Marika Urbanski, Emmanuelle Volle, Michel Thiebaut de Schotten

**Affiliations:** 1 Brain Connectivity and Behaviour Group, Sorbonne Universities, Paris France; 2 Frontlab, Institut du Cerveau et de la Moelle épinière (ICM), UPMC UMRS 1127, Inserm U 1127, CNRS UMR 7225, Paris, France; 3 Centre de Neuroimagerie de Recherche CENIR, Groupe Hospitalier Pitié-Salpêtrière, Paris, France; 4 Abnormal Movements and Basal Ganglia team, Inserm U 1127, CNRS UMR 7225, Sorbonne Universities, UPMC Univ Paris 06, Institut du Cerveau et de la Moelle épinière, ICM, Paris, France; 5 APHP, Urgences Cérébro-Vasculaires, Groupe Hospitalier Pitié-Salpêtrière, Paris, France; 6 Medicine and Rehabilitation Department, Hôpitaux de Saint-Maurice, Saint-Maurice, France

**Keywords:** Brain, MRI, Lesion, Statistics, Software, Open source, Connectivity, Disconnection, Behaviour

## Abstract

**Background:**

Patients with brain lesions provide a unique opportunity to understand the functioning of the human mind. However, even when focal, brain lesions have local and remote effects that impact functionally and structurally connected circuits. Similarly, function emerges from the interaction between brain areas rather than their sole activity. For instance, category fluency requires the associations between executive, semantic, and language production functions.

**Findings:**

Here, we provide, for the first time, a set of complementary solutions for measuring the impact of a given lesion on the neuronal circuits. Our methods, which were applied to 37 patients with a focal frontal brain lesions, revealed a large set of directly and indirectly disconnected brain regions that had significantly impacted category fluency performance. The directly disconnected regions corresponded to areas that are classically considered as functionally engaged in verbal fluency and categorization tasks. These regions were also organized into larger directly and indirectly disconnected functional networks, including the left ventral fronto-parietal network, whose cortical thickness correlated with performance on category fluency.

**Conclusions:**

The combination of structural and functional connectivity together with cortical thickness estimates reveal the remote effects of brain lesions, provide for the identification of the affected networks, and strengthen our understanding of their relationship with cognitive and behavioral measures. The methods presented are available and freely accessible in the *BCBtoolkit* as supplementary software [1].

Recent advances in neuroimaging techniques have allowed for further examination of the structural and functional organization of the human brain. While diffusion weighted imaging (DWI) tractography [2] depicts how brain areas are connected together, functional magnetic resonance imaging (*f*MRI) measures the activity within and interaction between brain areas [3]. These methods have been successfully applied to the healthy human brain; however, they remain underused in patients with brain lesions.

Patients with brain lesions provide a unique opportunity to understand the functioning of the human mind. Lesion symptom mapping analyses traditionally assume that visible and directly damaged areas are responsible for a patient's symptoms [4–7]. Following this logic, the areas that are the most frequently damaged by the lesion are considered as the neuronal substrate for the function. Previous studies that used this method have identified critical areas dedicated to, for example, language production [8], comprehension [9], spatial awareness [10–13], and other high-level cognitive functions [14–17]. However, anatomical disconnections between regions are also important considerations for the exploration of cognitive deficits [18, 19]. The dysfunction of distant areas that are connected to the lesioned tissue has also been reported in *f*MRI studies. These studies have shown that the networks are disrupted even by distant lesions through disconnection and diaschisis mechanisms [20–22].

Nonlocal effects of lesions have previously been explored using various forms of atlas-based analyses of tract damage [23–32], lesion-driven tractography [32–34], disconnectome mapping [35–39], and lesion-driven resting-state *f*MRI (rs-*f*MRI) connectivity [34, 40]. However, determining what these methods actually measure and identifying how to properly combine them are not always clear to the scientific community. Furthermore, there is an extremely limited availability of free, open-source software that applies methods to measure the nonlocal effects of lesions. These resources and scientific tools remain very much inaccessible and present a potential threat to reproducible science [41].

Disconnections and diaschisis can have an impact on distant regions in several respects through maladaptive responses and pathological spread [42]. When disconnected from its inputs and outputs, a region can no longer contribute to the elaboration of the supported function. This phenomenon is called diaschisis [20, 21, 43]. Once deprived from its inputs and/or outputs, transneuronal degeneration in the region will occur [42], dendrite and synapse density will decrease in number, myelin content will be altered, and neurons will reduce in size or die through a mechanism called apoptosis, a programmed cell death [44–46]. Hence, a white matter disconnection leads to both functional and anatomical changes that extend well beyond the visible damage. New approaches are therefore required to capture the long-range effects that follow brain disconnections. For instance, cortical thickness [see, 47] and other volumetric [eg, voxel-based morphometry 48] analyses have been used to study the structural changes associated with brain lesions but have not been applied in the context of brain disconnection.

In response to this need, here, we provide a set of complementary solutions to measure both the circuit and the subsequent changes within the circuit that are caused by a lesion. We applied these methods to 37 patients with focal brain lesions following a stroke or surgical resection. We first assessed the risk of disconnection in well-known white matter tracts and tested their relationship with category fluency performance. Category fluency is an appropriate test to explore disconnection since it requires the associations between executive, semantic, and language production functions [49, 50]. We then developed a tractography-based approach in order to produce maps of the areas that are directly disconnected by the lesion and tested their relationship with category fluency performance. We additionally calculated the rs-*f*MRI connectivity of these areas to reveal the whole network of directly and indirectly disconnected regions that participate in category fluency. Finally, we explored potential microstructural changes in the latter disconnected regions by estimating neuronal loss or local connectivity degeneration derived from magnetic resonance-based measures of cortical thickness and resting-state *f*MRI entropy.

## Methods

### Participants and category fluency task

Thirty-seven right-handed patients (French native speakers; 19 females; mean age 48 ±14.2 years, age ranging from 23 to 75 years) who presented with a frontal lobe lesion at the chronic stage (>3 months) were included in this study (see Table 1 for demographics). These patients were recruited from the stroke unit and the neuroradiology department at Salpêtrière Hospital, the neurological unit at Saint-Antoine Hospital, and the neuroradiology department at Lariboisière Hospital in Paris. Patients with a history of psychiatric or neurological disease, drug abuse, or MRI contraindications were not included. Additionally, we gathered behavioral data from 54 healthy participants (French native speakers; 27 females; mean age 45.8 ±14.4 years, age ranging from 22 to 71 years) in order to constitute a normative group.

**Table 1:** Demographical and clinical data

ID	Age (years)	Education (years)	Gender	Lesion side	Lesion volume (mm^3^)	Lesion delay (months)	Etiology
P01	56	17	F	Right	255	7	Stroke
P02	55	19	M	Left	34374	76	Hematoma
P03	46	17	F	Left	14847	126	Stroke
P04	50	11	F	Left	110145	137	Surgery
P05	64	14	M	Right	59048	119	Stroke
P06	32	16	F	Right	15946	129	epilepsy
P07	51	11	M	Bilateral	113170	54	Stroke
P08	70	5	F	Left	51530	85	Surgery
P09	47	11	M	Right	7809	115	Hematoma
P10	62	13	F	Bilateral	21295	14	Hematoma
P11	41	16	M	Right	55848	29	Surgery
P12	46	12	M	Bilateral	2542	51	Hematoma
P13	67	15	M	Left	4102	133	Stroke
P14	49	9	M	Bilateral	14929	19	Hematoma
P15	36	14	F	Right	40854	82	Surgery
P16	40	22	F	Left	24829	56	Hematoma
P17	40	14	M	Bilateral	14364	7	Hematoma
P18	23	16	F	Right	21681	47	Surgery
P19	54	22	M	Right	51897	48	Stroke
P20	71	17	M	Left	25779	91	Hematoma
P21	23	15	F	Right	29513	36	Surgery
P22	27	9	F	Left	12986	30	Surgery
P23	26	13	F	Left	2640	19	Surgery
P24	32	14	F	Left	12653	4	Surgery
P25	59	16	F	Left	97	9	Hematoma
P26	26	13	F	Left	26928	32	Stroke
P27	58	12	M	Left	1026	3	Stroke
P29	75	12	F	Left	14938	16	Hematoma
P30	52	13	F	Right	11978	20	Surgery
P31	58	12	M	Right	13263	21	Surgery
P32	62	5	M	Right	20281	9	Surgery
P33	41	17	M	Left	7463	29	Surgery
P34	42	17	M	Left	24319	6	Infection
P35	60	12	M	Right	41897	24	Surgery
P36	51	14	F	Right	39213	17	Surgery
P37	51	12	F	Right	8133	48	Surgery
P38	33	17	M	Right	140947	48	Surgery

All participants performed a category fluency task [51] in French. They were instructed to enumerate as many animals as possible during a timed period of 120 seconds. A clinical neuropsychologist (M. U.) recorded the results. Repetition and declination of the same animal were not taken into account in the final category fluency score.

The local ethics committee (Comités de protection des personnes, CPP Ile de France VI, Groupe hospitalier Pitie Salpetriere, reference project number 16-10) approved the experiment. All participants provided written, informed consent in accordance with the Declaration of Helsinki. Participants also received a small indemnity for their participation.

### Magnetic resonance imaging

An axial 3-dimensional magnetization prepared rapid gradient echo dataset covering the entire head was acquired for each participant (176 slices, voxel resolution = 1 × 1 × 1 mm, echo time = 3 msec, repetition time = 2300 msec, flip angle = 9°).

Additionally, the same participants underwent an *f*MRI session of resting state. During the resting-state session, participants were instructed to relax, keep their eyes closed, and avoid falling asleep. Functional images were obtained using T2-weighted echo-planar imaging with blood oxygenation level–dependent contrast using SENSitivity Encoding (SENSE) imaging, an echo time of 26 msec, and a repetition time of 3000 msec. Each dataset comprised 32 axial slices acquired continuously in ascending order covering the entire cerebrum with a voxel resolution of 2 × 2 × 3 mm; 200 volumes were acquired using these parameters for a total acquisition time of 10 minutes.

Finally, DWI was also acquired for 54 participants of the normative group (French native speakers; 27 females; mean age 45.8 ±14.4 years, age ranging from 22 to 71 years) and consisted of 70 near-axial slices acquired using a fully optimized acquisition sequence for the tractography of DWI, which provided isotropic (2 × 2 × 2 mm) resolution and coverage of the entire head with a posterior–anterior phase of acquisition. The acquisition was peripherally gated to the cardiac cycle [52], with an echo time = 85 msec. We used a repetition time equivalent to 24 RR (ie, interval of time between 2 heart beat waves). At each slice location, 6 images were acquired with no diffusion gradient applied. Additionally, 60 diffusion-weighted images were acquired in which gradient directions were uniformly distributed on the hemisphere with electrostatic repulsion. The diffusion weighting was equal to a *b*-value of 1500 s/mm^2^.

### Stereotaxic space registration

As spatial normalization can be affected by the presence of a brain lesion, additional processing was required before the normalization could be calculated. For instance, in the case of bilateral lesions, the registration was weighted as previously reported [53]. For unilateral lesions, the first step was to produce an enantiomorphic filling of the damaged area [54]. Each patient's lesion (or signal abnormalities due to the lesion) was manually segmented (using FSLview; [55]). Unilateral lesions were replaced symmetrically by the healthy tissue of the contralateral hemisphere. Enantiomorphic T1 images were fed into FMRIB's Automated Segmentation Tool (FAST) [56] for estimation of the bias field and subsequent correction of radiofrequency field inhomogeneity. This improved the quality of the automated skull stripping performed using a brain extraction tool (BET) [57] and the registration to the MNI152 using affine and diffeomorphic deformations [58]. The original T1 images (non enantiomorphic) were registered to the MNI152 space using the same affine and diffeomorphic deformations as calculated above. Subsequently, lesions were segmented again in the MNI152 space under the supervision of an expert neurologist (E. V.). This method has been made freely available as the tool *normalisation* as part of *BCBtoolkit* [1].

The following sections are hypotheses driven and outlined in Supplementary Fig. 1.

**Figure 1:**
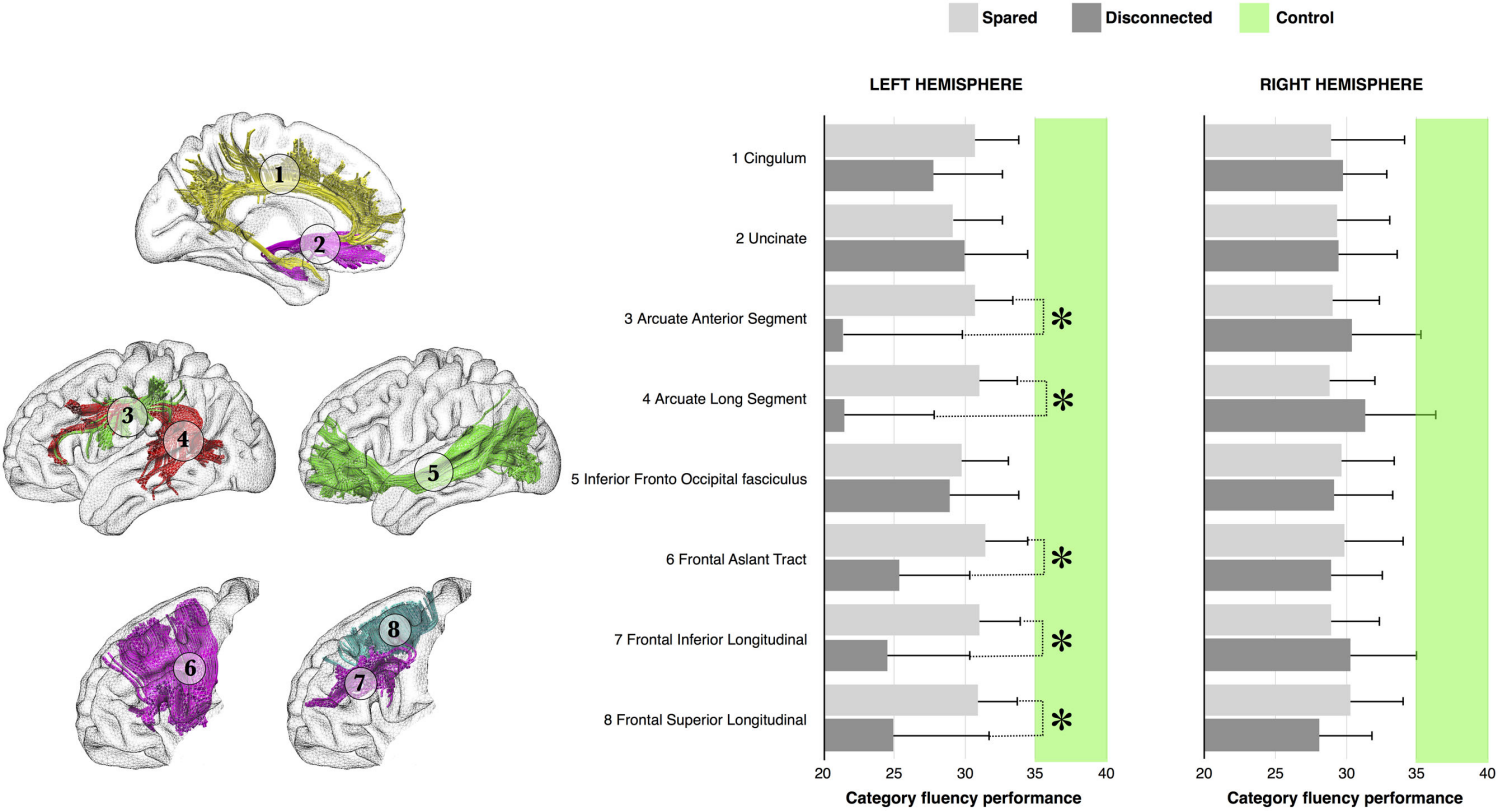
Category fluency performance (mean performance with 95% confidence intervals [CIs]) for patients with (dark gray) or without (light gray) disconnection of each tract of interest. The green intervals indicate the range of controls’ performance corresponding to 95% CIs. * *P* < 0.05.

### White matter tracts disconnection

Each patient's lesion was compared with an atlas of white matter tracts [59], indicating for each voxel, the probability of finding a white matter tract such as the arcuate fasciculus, the frontal aslant tract, or the uncinate fasciculus in the MNI152 coordinate system. We considered a tract to be involved when the likelihood of a tract being present in a given voxel was estimated above 50% [23]. This method is freely available as *tractotron* in *BCBtoolkit* [1]. We focused on frontal lobe tracts with a potential effect on executive, semantic, and language functions since all of the patients had a frontal lesion. These tracts included the cingulum, the frontal aslant, and the frontal superior and inferior longitudinal tracts for the executive functions [60]; the uncinate and the inferior fronto-occipital fasciculi for the semantic access [61, 62]; and the anterior and long segment of the arcuate fasciculi for the phonemic system [63, 64]. A Kruskal-Wallis test was used to compare performance on the category fluency test for each tract between both preserved and disconnected patients and control participants. Subsequently, for each significant tract between patients, Mann-Whitney post hoc comparisons were performed (Fig. 1).

### Direct disconnection of brain areas: structural connectivity network

This approach used the DWI datasets of 10 participants in the normative group to track fibers that passed through each lesion.

For each participant, tractography was estimated as indicated in [65].

Patients’ lesions in the MNI152 space were registered to each control native space using affine and diffeomorphic deformations [58] and, subsequently, used as seed for the tractography in Trackvis [66]. Tractography from the lesions were transformed in visitation maps [67, 68], binarized, and brought to the MNI152 using the inverse of precedent deformations. Finally, we produced a percentage overlap map by summing at each point in the MNI space the normalized visitation map of each healthy patient. Hence, in the resulting *disconnectome map*, the value in each voxel took into account the interindividual variability of tract reconstructions in controls and indicated a probability of disconnection from 50% to 100% for a given lesion (ie, thus explaining more than 50% of the variance in disconnection and corresponding to a large effect size). This procedure was repeated for all lesions, allowing the construction of a *disconnectome map* for each patient/lesion. These steps were automatized in the tool *disconnectome map* as part of the *BCBtoolkit*. Note that sample size and age effects are carefully explored and reported in the Supplementary Material. Overall, 10 patients are sufficient to produce a good enough *disconnectome map* that matches the overall population (more than 70% of shared variance). We also demonstrate in the Supplementary Material that *disconnectome maps* show a very high anatomical similarity between decades and no decrease of this similarity with age.

**Figure 2:**
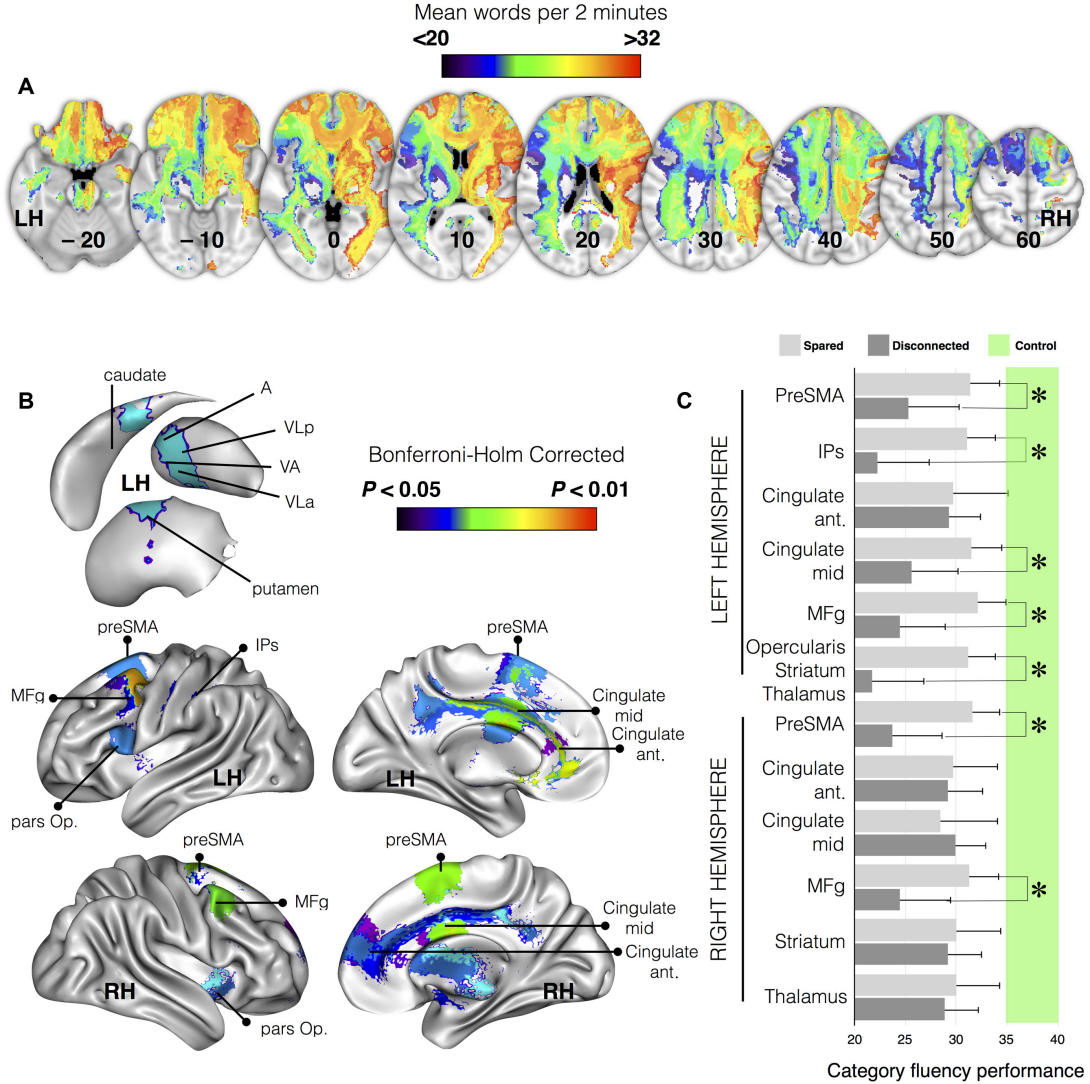
Areas directly disconnected by the lesion that significantly contributed to a decreased score on category fluency task (referred to as “disconnected areas” in the manuscript). A) Representative slices from *disconnectome maps* computed for category fluency performance; blue clusters indicate group average low performance and red clusters indicate high performance. B) Brain areas contributing significantly after correction for multiple comparisons. C) Category fluency performance (mean performance with 95% confidence intervals [CIs]) for patients with (dark gray) or without (light gray) disconnection of each of the examined cortical regions. The green interval indicates performance in matched controls with 95% CIs. Abbreviations: A, anterior group of thalamic nuclei; LH, left hemisphere; IPs, intraparietal sulcus; MFg, middle frontal gyrus; pars Op., frontal pars opercularis; PreSMA, presupplementary motor area; RH, right hemisphere; VA, ventral anterior; VLa, ventrolateral anterior; VLp, ventrolateral posterior. * *P* < 0.05 Bonferroni-Holm corrected for multiple comparisons.

Thereafter, we used *AnaCOM2*, which is available within the *BCBtoolkit*, in order to identify the disconnections that are associated with a given deficit, that is, connections that are critical for a given function (Fig. 2). *AnaCOM2* is comparable to *AnaCOM* [69] but has been reprogrammed and optimized to work on any Linux or Macintosh operating systems.

Initially, *AnaCOM* is a cluster-based lesion symptom mapping approach that identifies clusters of brain lesions that are associated with a given deficit, that is, the regions that are critical for a given function. In the context of this article, *AnaCOM2* used *disconnectome maps* instead of lesion masks to identify clusters of disconnection that are associated with category fluency deficits, that is, the connections that are critical for a given function. Compared to standard voxel-based lesion-symptom mapping (VLSM) [8], *AnaCOM2* regroups voxels with the same distribution of neuropsychological scores into clusters of voxels. Then, for each cluster larger than 8 mm^3^, *AnaCOM2* will perform a Kruskal-Wallis test between patients with a disconnection, patients spared of disconnection, and controls. Resulting *P* values are Bonferroni-Holm corrected for multiple comparisons. Subsequently, significant clusters (*P* value < 0.05) are used to perform a post hoc Mann-Whitney comparison between 2 subgroups of interest (ie, disconnected patients and healthy participants). Post hoc results are Bonferroni-Holm corrected for multiple comparisons (statistical tests and corrections are computed using R language [70]).

Patient–control comparisons have been chosen as a first step in order to avoid drastic reduction of statistical power when 2 or more nonoverlapping areas are responsible for patients’ reduced performance [69]. Nonparametric statistics have been chosen, as it is fair to consider that some clusters will not show a Gaussian distribution. *AnaCOM2* resulted in a statistical map that reveals, for each cluster, the significance of a deficit in patients who undertake a given task as compared to controls.

In the following sections, the term “clusters” systematically refers to the result of the post hoc Mann-Whitney comparison between disconnected patients and healthy participants who survived Bonferroni-Holm correction for multiple comparisons.

### fMRI meta-analyses

**Figure 3:**
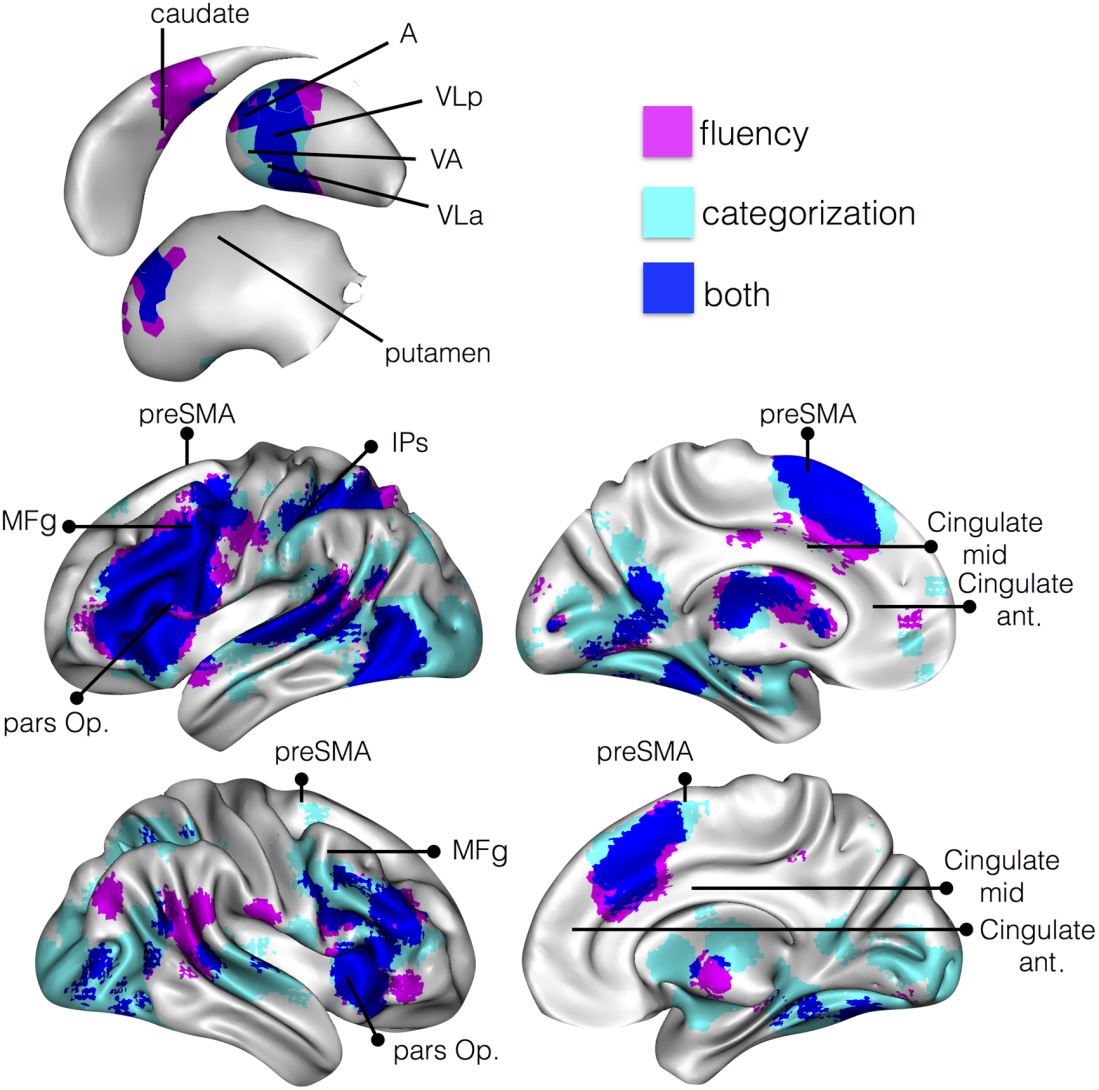
Areas classically activated with functional magnetic resonance imaging (*P* < 0.01 corrected for False Discovery Rate) during fluency (pink) and categorization (cyan) tasks. Areas involved in both fluency and categorization are highlighted in dark blue. Abbreviations: A, anterior group of thalamic nuclei; IPs, intraparietal sulcus; MFg, middle frontal gyrus; PreSMA, presupplementary motor area; VA, ventral anterior; VLa, ventrolateral anterior; VLp, ventrolateral posterior.

A method described by Yarkoni et al. [71, 72] was used to identify the functional networks involved in category fluency. We searched for brain regions that are consistently activated in studies that load highly on the following 2 features: “fluency” (120 studies, 4214 activations) and “category” (287 studies, 10179 activations). The results were superimposed on the 3D reconstruction of the MNI152 images (Fig. 3).

### Indirect disconnection of brain areas: functional connectivity network

Rs-*f*MRI images were first motion corrected using MCFLIRT [73], then corrected for slice timing, smoothed with a full half width maximum equal to 1.5 times the largest voxel dimension, and finally filtered for low temporal frequencies using a Gaussian-weighted local fit to a straight line. These steps are available in Feat as part of the FSL package [74].

Rs-*f*MRI images were linearly registered to the enantiomorphic T1 images and, subsequently, to the MNI152 template (2 mm) using affine transformations. Confounding signals were discarded from rs-*f*MRI by regressing out a confound matrix from the functional data. The confound matrix included the estimated motion parameters obtained from the previously performed motion correction, the first eigenvariate of the white matter and cerebrospinal fluid (CSF) as well as their first derivative. Eigenvariates can easily be extracted using fslmeants combined with the –eig option. White matter and CSF eigenvariates were extracted using masks based on the T1-derived 3-classes segmentation thresholded to a probability value of 0.9, registered to the rs-*f*MRI images, and binarized. Finally, the first derivative of the motion parameters, white matter, and CSF signal was calculated by linear convolution between their time course and a [−1 0 1] vector.

For each control participant, we extracted the time course that corresponded to each significant cluster, which was identified by the statistical analyses of the *disconnectome maps*. These time courses were subsequently correlated to the rest of the brain so as to extract seed-based resting-state networks. In order to obtain the most representative networks at the group level, for each seed-based resting-state network, we calculated the median network across the group. The median network resulting from a seed contains, in each voxel, the median of functional connectivity across all the controls. Medians were chosen instead of average as they are less sensitive to outliers and are more representative of the group-level data [75]. The calculation of the functional connectivity was automatized and made available inside the *funcon* tool as part of *BCBtoolkit*. Medians were calculated using the function fslmaths.

**Figure 4:**
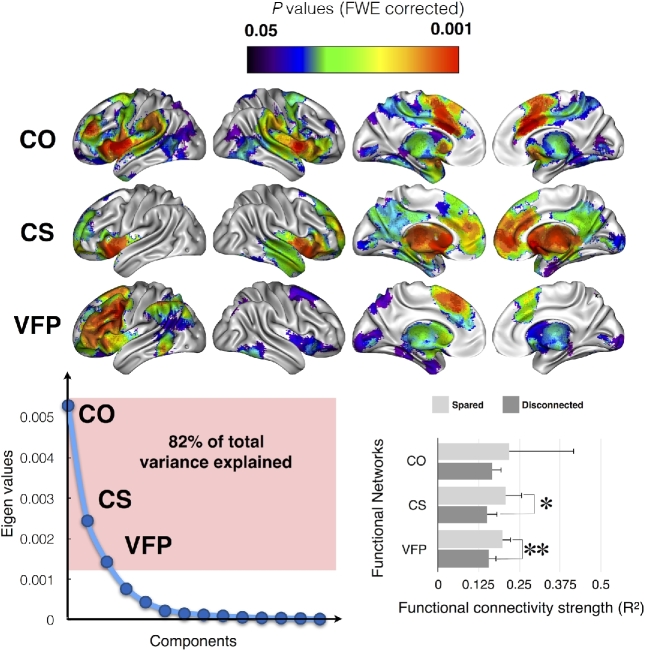
Functional networks involving the identified disconnected areas, as defined by resting state functional connectivity. Top panel, Main cortical networks involving the disconnected areas revealed by a principal component analysis. Bottom left panel, Principal component analysis of the raw functional connectivity result. Bottom right panel, Strength of the functional connectivity for patients with (dark gray) or without (light gray) involvement of the functional network. Abbreviations: CO, cingulo-opercular network; CS, cortico-striatal network; VFP, ventral fronto-parietal network. * *P* < 0.05; **, *P* < 0.01.

Visual inspection revealed that several of these resting-state networks shared a very similar distribution of activations. Therefore, an “activation” matrix was derived from the seed-based resting-state networks. This matrix consisted of columns that indicated each seed-based resting-state network and rows that represented the level of activation for each voxel in the cortex. This activation matrix was entered into a principal component analysis in SPSS (Chicago, Illinois) using a covariance matrix and varimax rotation (with a maximum of 50 iterations for convergence) in order to estimate the number of principal components to extract for each function. Components were plotted according to their eigenvalue (y; lower left panel in Fig. 4); we applied a scree test to separate the principal from residual components. This analysis revealed that 3 factors were enough to explain 82% of the variance of the calculated seed-based resting-state networks. This means that 3 factors are good enough to summarize most of the seed-based resting-state network results. Finally, brain regions that had a statistically significant relationship with the 3 components (ie, factor-networks) were detected using a linear regression with 5.000 permutations, in which the eigenvalues of the 3 components represented the independent variable and the seed-based resting-state networks represented the dependent variable. Results were Family Wise Error–corrected for multiple comparisons and projected onto the average 3D rendering of the MNI152 template in the top panel of Fig. 4. In the following sections, the term “factor-networks” systematically refers to brain regions that have a statistically significant relationship with the 3 components.

Additionally, for each patient, we extracted the time course that corresponded to each factor-network. These time courses were subsequently correlated to the rest of the brain so as to extract seed-based factor-networks in each patient. FSLstats was used to extract the strength of factor-networks functional connectivity and, subsequently, to compare patients according to their disconnection status. Note that a patient disconnected in a factor-network is a patient who has a disconnection in at least 1 of the clusters that contributed significantly to the factor-network.

### Structural changes in disconnected regions

A distant lesion can affect cortical macro- and microstructures remotely. Conscious of this, we attempted to estimate these structural changes and their relationship with category fluency within each functional factor-network. To this aim, we explored the properties of each functional network using the following 2 complementary measures: T1w-based cortical thickness to identify fine local volumetric changes and the Shannon entropy of rs-*f*MRI as a surrogate for the local complexity of the neural networks [76]. Each original functional network seeded from each cluster was thresholded and binarized at r > 0.3 and used as a mask to extract cortical thickness and entropy. Patients’ lesions were masked out for these analyses.

For the cortical thickness, a registration-based method (Diffeomorphic Registration based Cortical Thickness, DiReCT) was used [77] from the T1-weighted imaging dataset. The first step, as for the *normalization*, was to produce an enantiomorphic filling of the damaged area in order to prevent the analysis from being contaminated by the lesioned tissue. The second step of this method consisted of creating two 2-voxel thick sheets, 1 laying just between the gray matter and the white matter and the second laying between the gray matter and the CSF. The gray–white interface was then expanded to the gray–CSF interface using diffeomorphic deformation estimated with Advanced Normalization Tools (ANTs). The registration produced a correspondence field that allowed an estimate of the distance between the gray–white and the gray–CSF interfaces and thus corresponded to an estimation of cortical thickness. Voxels that belonged to the lesion were subsequently removed from the cortical thickness maps (see Supplementary Fig. S2). This approach has good scan–rescan repeatability and good neurobiological validity as it can predict with a high statistical power the age and gender of the participants [78] as well as atrophy following brain lesions [79]. Note that the striatum and thalamus were excluded from the cortical thickness analysis since they do not have a cortical ribbon.

Shannon entropy is an information theory–derived measure that estimates signal complexity [80, 81]. In the context of rs-*f*MRI, the entropy measures the local complexity of the blood oxygen level–dependent (BOLD) signal as a surrogate of the complexity of the spontaneous neuronal activity [82, 83]. Since “cells that fire together wire together” [84], for each gray matter voxel, Shannon entropy of rs-fMRI can be considered as a surrogate for the complexity of the connections within this voxel and between this voxel and the rest of the brain. Shannon entropy was extracted from the previously preprocessed rs-*f*MRI using the following formula: − sum (p*log(p)), where p indicates the probability of the intensity in the voxel [76].

FSLstats was used to extract the average cortical thickness and resting state *f*MRI entropy for each cluster and factor-network. Statistical analysis was performed using SPSS software. In our analysis, Gaussian distribution of the data was not confirmed for the cortical thickness and the entropy measures using the Shapiro–Wilk test. Therefore, nonparametric statistics were chosen to compare cortical thickness and entropy levels between patients disconnected, patients spared, and controls in each cluster and factor-network. Additionally, bivariate Spearman rank correlation coefficient analyses were performed between the cortical thickness or entropy measurement of each functional network and each patient's category fluency performance. Correlation significant at *P* < 0.0041 survives Bonferroni correction for multiple comparisons (12 networks).

## Results

### White matter tracts disconnection

Patients’ lesions were compared to an atlas of white matter connections in order to identify the probability of tract disconnections [59]. A Kruskal-Wallis test indicated that for each tract, patients (ie, connected and disconnected) and control participants showed a significantly different performance on the category fluency test (all *P* < 0.001; full statistics reported in Table 2). Between patients, post hoc comparisons revealed that disconnections of the left frontal aslant (U = 90.0; *P* = 0. 0389), frontal inferior longitudinal (U = 69.0; *P* = 0. 0216) and frontal superior longitudinal (U = 75.0; *P* = 0. 0352) tracts, and the anterior (U = 28.5; *P* = 0. 0116) and long segment (U = 31.5; *P* = 0.0059) of the arcuate fasciculus were associated with a poorer performance in category fluency (Fig. 1). However, these post hoc comparisons did not survive Bonferroni-Holm correction for multiple comparisons.

**Table 2:** White matter tracts disconnection relationship with category fluency statistical report

	3 groups comparison		Patients disconnected and connected		Patients disconnected and controls		Patients connected and controls		n1^a^	n2^b^
Tract	K	*P* value	U	*P* value	U	*P* value	U	*P* value		
Cingulum left	19	0.0001	141	0.2035	189	0.0003	277	0.0003	16	21
Cingulum right	19	0.0001	161	0.5	280	0.0001	187	0.0019	23	14
Uncinate left	19	0.0001	148	0.3994	176	0.0027	291	0.0001	13	24
Uncinate right	19	0.0001	167	0.4635	209	0.0004	258	0.0003	17	20
Arcuate anterior segment left	22	0.0000	29	0.0116	12	0.0004	454	0.0001	5	32
Arcuate anterior segment right	19	0.0001	126	0.3855	118	0.0025	348	0.0001	16	21
Arcuate long segment left	23	0.0000	32	0.0059	13	0.0001	453	0.0002	6	31
Arcuate Long segment right	19	0.0001	107	0.2559	117	0.0068	349	0.0001	9	28
Inferior fronto-occipital fasciculus left	19	0.0001	165	0.5	196	0.0011	271	0.0001	15	22
Inferior fronto-occipital fasciculus right	19	0.0001	157	0.3457	199	0.0002	268	0.0004	17	20
Frontal aslant tract left	21	0.0000	90	0.0389	90	0.0001	377	0.0004	11	26
Frontal aslant tract right	19	0.0001	155	0.3131	194	0.0001	272	0.0012	18	19
Frontal inferior longitudinal left	21	0.0000	69	0.0216	54	0.0001	413	0.0004	9	28
Frontal inferior longitudinal right	19	0.0001	140	0.3051	171	0.0022	295	0.0001	13	34
Frontal superior longitudinal left	20	0.0000	75	0.0352	73	0.0004	393	0.0002	9	28
Frontal superior longitudinal right	19	0.0001	129	0.1992	120	0.0001	346	0.0005	13	34

Results are not corrected for multiple comparisons.

^a^Number of disconnected patients.

^b^Number of spared patients.

These results indicate that poor performance measured in patients with brain damage can be associated to some extent with white matter tract disconnections.

### Direct disconnection of brain areas: structural connectivity network

As different white matter atlases exist for the interpretation of the white matter tract disconnection [85] and atlas-based approaches cannot assess the disconnection of the subportion of tracts nor the involvement of multiple tracts by a lesion, data-driven maps of disconnection or “disconnectomes” were produced. Using tractography in a group of 10 healthy controls, the registered lesions were used as a seed to reveal white matter tracts that passed through the injured area so as to produce maps of disconnections, later referred to as *disconnectome maps*. Category fluency scores were attributed to each patient's *disconnectome map* (see Fig. 2A). A Kruskal-Wallis test indicated that for several clusters, patients (ie., connected and disconnected) and control participants showed a significantly different performance on the category fluency test (all *P* < 0.001; full statistics reported in Table 3).

**Table 3:** Direct disconnection of brain areas relationship with category fluency statistical report

Fluency score		3 groups comparison		Patients disconnected and connected (uncorrected)		Patients disconnected and controls		Patients connected and controls		n1^a^	n2^b^
	Disconnected areas	Kruskal-Wallis	*P* value	U	*P* value	U	*P* value	U	*P* value		
Left hemisphere	PreSMA	21.34128456	0.0059	212	0.0456	88.5	0.0248	377.5	0.0329	12	25
	IPs	23.35102548	0.0023	172	0.0098	18	0.0295	448	0.0324	7	30
	Cingulate ant	18.6697471	0.0125	160.5	0.7452	304	0.0248	162	0.0329	25	12
	Cingulate mid	21.52289636	0.0054	219	0.0464	95.5	0.0141	370.5	0.0329	13	24
	MFg	23.12826675	0.0026	237	0.0103	81.5	0.0054	384.5	0.0329	13	24
	Opercularis, striatum, thalamus	23.99647373	0.0017	179	0.0043	16	0.0249	450	0.0324	7	30
Right hemisphere	PreSMA	22.92724537	0.0028	208	0.0130	50.5	0.0138	415.5	0.0329	10	27
	Cingulate ant	18.8698263	0.0125	185.5	0.6470	225	0.0330	241	0.0329	20	17
	Cingulate mid	18.62681983	0.0125	137.5	0.6966	317	0.0415	149	0.0329	25	12
	MFg	22.06856039	0.0042	196	0.0382	54	0.0180	412	0.0329	10	27
	Striatum	18.604408	0.0125	154.5	0.8966	310	0.0313	156	0.0329	14	23
	Thalamus	19.117101	0.0125	192	0.5326	202	0.0248	264	0.0329	14	23

Unless specified, *P* values are Bonferroni-Holms corrected for multiple comparisons.

Abbreviation: MFg, middle frontal gyrus; SMA, supplementary motor area.

^a^Number of disconnected patients.

^b^Number of connected patients.

Results were further statistically assessed using Mann-Whitney post hoc comparisons in order to identify areas that, when deafferented due to a disconnection mechanism, lead to a significant decrease in performance in category fluency when compared to controls.

The following results are Bonferroni-Holm corrected for multiple comparisons. Main cortical areas in the left hemisphere included the pre supplementary motor area (preSMA; cluster size = 1449; Mann Whitney U = 88.5; *P* = 0.025), the anterior portion of the intraparietal sulcus (IPs; cluster size = 1143; U = 18; *P* = 0.030), anterior cingulate gyrus (cluster size = 837; U = 304; *P* = 0.025) and middle cingulate gyrus (cluster size = 898; U = 95.5; *P* = 0.014), the middle frontal gyrus (MFg; cluster size = 829; U = 81.5; *P* = 0.005), and the pars opercularis of the inferior frontal gyrus (cluster size = 5314; U = 16; *P* = 0.025).

In the right hemisphere, the preSMA (cluster size = 1050; U = 50.5; *P* = 0.014), the MFg (cluster size = 552; U = 54; *P* = 0.018), the anterior cingulate gyrus (cluster size = 572; U = 44.5; *P* = 0.009), and the middle cingulate gyrus (cluster size = 817; U = 317; *P* = 0.041) were also involved (Fig. 2B).

Subcortical areas in the left hemisphere involved the caudate, the putamen, and several ventral thalamic nuclei including the ventral anterior, the ventrolateral anterior, and the ventrolateral posterior as a part of the same cluster (cluster size = 5314; U = 16; *P* = 0.025).

In the right hemisphere, the striatum (cluster size = 527; U = 310; *P* = 0.031) and the ventral thalamic nuclei (cluster size = 935; U = 202.0; *P* = 0.025) were also involved (Fig. 2B).

Additionally, between-patient (ie, connected and disconnected, uncorrected for multiple comparisons) comparisons confirmed the critical involvement of the preSMA (U = 212; *P* = 0.0456), the MFg (U = 237; *P* = 0.01), the pars opercularis (U = 179; *P* = 0.004), and the IPs (U = 172; *P* = 0.01) in the left hemisphere. The preSMA (U = 208; *P* = 0.01) and the MFg (U = 196; *P* = 0.038) were also involved in the right hemisphere (Fig. 2C). Full statistics are reported in Table 3.

### fMRI Meta-analyses

We further examined whether the disconnected areas in patients with poor performance are functionally engaged in tasks related to fluency and categorization using a meta-analysis approach [71, 72].

The result indicates that disconnected areas reported as significantly contributing to category fluency performance in patients are classically activated by functional MRI tasks that require either fluency or categorization in healthy controls (Fig. 3).

### Indirect disconnection of brain areas: functional connectivity network

As the *disconnectome mapping* method cannot measure the indirect disconnection produced by a lesion (ie, it fails to measure the disconnection in a region that is not directly anatomically connected to a damaged area but that nonetheless remains a part of the same large network of functionally connected areas), we used functional connectivity in healthy controls. This allowed us to reveal the entire network of regions that are functionally connected to the areas that were reported as contributing significantly to the category fluency performance when directly disconnected. When compared to tractography, functional connectivity has the added advantage of revealing the areas that contribute to the network through both direct and indirect structural connections.

Principal component analysis indicated that the significant areas that contributed to category fluency performance belonged to 3 main functional networks (ie, factor-networks) (Fig. 4), which accounted for more than 80% of the total variance of the functional connectivity results.

The left cingulate clusters (anterior and middle), the right anterior cingulate, the middle frontal gyrus, the thalamus, and the operculum all belonged to the cingulo-opercular network (CO) [86], including the right preSMA, posterior cingulate, and rostral portion of the middle frontal gyrus.

The middle of the cingulate gyrus and the striatum in the right hemisphere both belonged to a cortico-striatal network (CS) [87] that involves the right thalamus and striatum.

Finally, the left MFg, preSMA, IPs, pars opercularis, thalamus, and striatum were all involved in a larger, left ventral fronto-parietal network (VFP), which also included other areas such as the right preSMA, the frontal eye field, and the temporo-parietal junction [88].

Additional analyses revealed the differences in the functional connectivity of these factor-networks relative to the disconnected status of areas involved in category fluency. Between-patient (ie, connected and disconnected) comparisons revealed significantly lower functional connectivity in the left VFP network (U = 54.0; *P* = 0.006) and in the CS network (U = 63.0; *P* = 0.027) when anatomically disconnected. The CO network, however, did not show any significant difference (U = 40.0; *P* = 0.213). Overall, the strength of the functional connectivity for each patient did not correlate significantly with the fluency performance.

### Structural changes in disconnected regions

Additional exploratory analyses revealed structural changes related to the disconnections. We estimated these changes using the following 2 complementary measures: T1w-based cortical thickness to identify fine local volumetric changes and the Shannon entropy of rs-*f*MRI as a surrogate for the local complexity of the neural networks [76].

When compared to controls, patients showed a reduced cortical thickness in the left pars opercularis (H = 13; *P* = 0.0012), MFg (H = 8; *P* = 0.0143), preSMA (H = 8; *P* = 0.0224), IPs (H = 9; *P* = 0.0131), and right anterior (H = 7; *P* = 0.0296) and middle cingulate gyrus (H = 23; *P* = 0.000). When compared to patients with no disconnection, only the right middle cingulate gyrus survived the Bonferroni-Holm correction for multiple comparisons (U = 67; *P* = 0.004). When compared to controls, disconnected patients showed reduced entropy for all regions (all *P* < 0.05, except for the right middle frontal gyrus). However, when compared to patients with no disconnection, none of the comparisons survived the Bonferroni-Holm correction for multiple comparisons. Uncorrected *P* values are reported as an indication in Table 4 and the bar chart in Supplementary Fig. S3.

**Table 4:** Cortical thickness and functional magnetic resonance imaging entropy measures in disconnected areas

Cortical thickness		3 groups comparison		Patients disconnected and connected		Patients disconnected and controls		Patients connected and controls		n1^a^	^n2^ ^b^
	Disconnected areas	Kruskal-Wallis	*P* value	U	*P* value	U	*P* value	U	*P* value		
Left hemisphere	PreSMA	8	0.0224	109	0.0944	168	0.0057	514	0.0565	12	25
	IPs	9	0.0131	54	0.0251	59	0.0019	667	0.1134	7	30
	Cingulate ant	5	0.0822	110	0.1000	465	0.0175	269	0.2061	25	12
	Cingulate mid	5	0.0759	139	0.2998	278	0.1436	435	0.0137	13	24
	MFg	8	0.0143	109	0.0695	172	0.0028	502	0.0710	13	24
	Opercularis	13	0.0012	40	0.0061	44	0.0006	583	0.0225	7	30
Right hemisphere	PreSMA	4	0.1328	134	0.4931	214	0.1711	523	0.0254	10	27
	Cingulate ant	7	0.0296	167	0.4696	362	0.0191	295	0.0169	20	17
	Cingulate mid	23	0.0000	61	0.0020	223	0.1414	254	0.1415	25	12
	MFg	6	0.0587	116	0.2634	163	0.0359	538	0.0359	10	27
Shannon entropy											
Left hemisphere	PreSMA	24	0.0000	86	0.2171	85	0.0004	210	0.0000	12	25
	IPs	27	0.0000	40	0.0422	18	0.0002	260	0.0000	7	30
	Cingulate ant	44	0.0000	84	0.1158	109	0.0000	3	0.0000	25	12
	Cingulate mid	36	0.0000	97	0.3029	45	0.0000	127	0.0000	13	24
	MFg	16	0.0004	100	0.4246	125	0.0043	272	0.0003	13	24
	Opercularis, striatum, thalamus	17	0.0002	65	0.3680	75	0.0181	287	0.0001	7	30
Right hemisphere	PreSMA	8	0.0177	82	0.2364	117	0.0078	413	0.0243	10	27
	Cingulate ant	55	0.0000	81	0.0640	16	0.0000	4	0.0000	20	17
	Cingulate mid	22	0.0000	111	0.4596	203	0.0001	114	0.0003	25	12
	MFg	22	0.0000	55	0.0497	136	0.0533	209	0.0000	10	27
	Striatum	23	0.0000	110	0.4436	202	0.0001	100	0.0001	14	23
	Thalamus	58	0.0000	67	0.0204	0	0.0000	6	0.0000	14	23

Results are not corrected for multiple comparisons.

Abbreviation: IPs, intraparietal sulcus; MFg, middle frontal gyrus; SMA, supplementary motor area.

^a^Number of disconnected patients.

^b^Number of spared patients.

None of these measures correlated significantly with the fluency performance.

**Figure 5:**
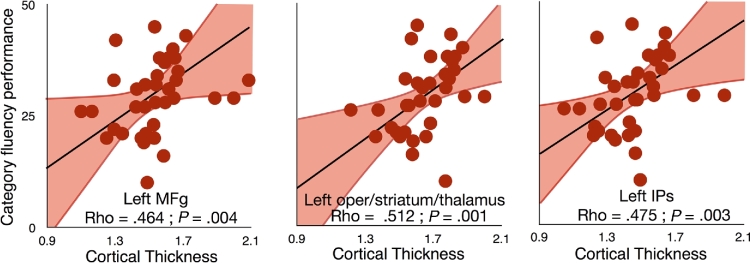
Dimensional relationship between cortical thickness measured in rs-fMRI disconnected networks and category fluency. Note that regression lines (in black) and intervals (mean confidence intervals in red) are for illustrative purposes since we performed a rank-order correlation. Abbreviations: IPs, intraparietal sulcus; MFg, middle frontal gyrus.

In order to further assess the integrity of the whole network of regions that were functionally connected to the areas reported as having significantly contributed to category fluency performance, we also extracted the cortical thickness and entropy from the regions that were functionally connected to the disconnected areas. Correlation analyses indicated that a thinner cortex in the ventral fronto-parietal network seeded from the left MFg (Spearman Rho = .464 ± 0.341; *P* = .004), IPs (Rho = .475 ± 0.341; *P* = .003), and left pars opercularis/striatum/thalamus (Rho = .512 ± 0.341; *P* = .001) corresponded to a reduced performance in category fluency (Fig. 5). Additionally, a thinner cortical thickness in the left preSMA functional network (Rho = .376 ± 0.341; *P* = .024) and a higher rs-*f*MRI entropy (Rho = −.420 ± 0.370; *P* = .019) in the mid cingulate gyrus functional network were associated with poorer performance in category fluency. These last 2 results, however, did not survive Bonferroni-Holm correction for multiple comparisons.

The same analyses were repeated controlling for age and lesion size and confirmed the results for the ventral fronto-parietal network seeded from the left MFg (Spearman Rho = .423; *P* = .01), IPs (Rho = .538; *P* = .001), and left opercularis (Rho = .590 ± 0.341; *P* < .001) and corresponded to a reduced performance in category fluency (Fig. 5). Additionally, a thinner cortical thickness in the left preSMA functional network (Rho = .439; *P* = .007) and a higher rs-*f*MRI entropy (Rho = −.420 ± 0.370; *P* = .019) in the mid cingulate gyrus functional network were associated with poorer performance in category fluency.

## Discussion

A large set of complementary methods can capture the impact of lesions on distant regions and expose the subsequent consequences on patients’ neuropsychological performance. Several of these methods are built directly into our freely available software package *BCBtoolkit*. This package can be used to measure the pathophysiological mechanisms that cause cognitive deficits and to assess the relationship between these mechanisms and their consequential effects. Here, we evaluated the risk of disconnection of classically defined white matter tracts and tested their relationship with category fluency performance. We then used a tractography-based approach to reveal regions that were structurally disconnected by the lesion and to assess their relationship with category fluency performance as compared to controls and other patients. Functional connectivity from the disconnected regions revealed large networks of interconnected areas. Within these regions/networks, measures of cortical thickness and entropy of the rs-*f*MRI images were correlated to fluency performance, suggesting that some structural changes that occurred within these networks were due to the remote effect of a lesion that led to cognitive impairments. Consequently, the *BCBtoolkit* provided investigators with an ability to quantify the effect of brain damage on the whole brain and to explore its relationship to behavioral and cognitive abilities.

The investigation into the contribution of white matter tract disconnection is an approach that is more than a century old and postulates an interruption in the course of white matter tracts in single case patients [89, 90]. Our method provides an anatomical rationale and puts forth a statistical methodology that enables it to be extended to group-level studies. In the case of category fluency performance, this analysis particularly revealed a significant involvement of the anterior and long segments of the arcuate fasciculus, which are implicated in the language network [90–92]. However, these tracts have been defined, for convenience, by their shape (eg, uncinate for hook-shaped connections and arcuate for arched-shaped connections) and should not be considered as a single unit, as, ultimately, subportions could contribute differently to the elaboration of the cognition and behavior.

Data-driven maps of disconnection, or *disconnectomes*, were consequently produced in order to identify the subportion of disconnected tracts and reveal the pattern of cortico-subcortical areas that were disconnected by the lesion. For the first time, we exemplify that this method goes beyond assessing only lesions and can be used to assess the relationship between disconnected areas and the patient's neuropsychological performance. Here, this approach revealed that category fluency performance significantly decreased when several cortical and subcortical clusters were directly disconnected. The observed areas are consistent with previous lesion studies on fluency tasks [93]. Furthermore, each area identified as significantly involved in this analysis corresponded, almost systematically, to activation loci derived from *f*MRI studies in healthy controls who performed fluency and/or categorization tasks. This result suggests that the method appropriately identified altered functional networks that contribute to the category fluency test. Nonetheless, one might argue that a cascade of polysynaptic events can influence behavior and that dysfunctional, disconnected areas will also impact other indirectly connected areas.

In order to explore this additional dimension, we calculated the functional connectivity of the previously identified disconnected regions (ie, clusters). In the case of the present analysis on category fluency performance, we revealed that the disconnected areas belonged to the following 3 large functional networks (ie, factor-networks): a left-dominant ventral fronto-parietal network; a mirror of the right-lateralized ventral attention network [94], which links key language territories [88] and is associated with executive functions [95, 96]. In addition, we showed the involvement of the cingulo-opercular network, a network that interacts with the fronto-parietal control network for the control of goal-directed behaviors [97], which together with the cortico-striatal network may also be linked to a reduced performance in fluency tasks [98]. The cingulo-opercular and cortico-striatal networks may also have contributed to performance through the global inertia or the ability of participants to allocate and coordinate resources during the task [99]. Finally, disconnection was associated with a significant reduction of functional connectivity in 2 of the 3 factor-networks investigated. This is an important result, as functional connectivity appeared to be less significantly impaired in bilateral networks, suggesting that the proportion of the preserved functional network in both of the intact hemispheres may contribute to the strength of functional connectivity.

Changes in connectivity should induce changes in the microstructure of the areas of projection and provoke cognitive or behavioural consequences. Measures of the cortical thickness revealed a significant thinning for some, but not all, directly disconnected areas. This result may reflect a potential transneuronal degeneration mechanism [42]. However, current limitations in spatial resolution and MRI signal might have biased this measure in some regions due to changes in myelination in the lower layers of the cortex [100]. Cortical thickness analyses revealed that the left dominant ventral fronto-parietal network, whether it is seeded from MFg, IPs, or subcortical structures in the left hemisphere, had a reduced cortical thickness associated with the category fluency performance. This result indicates a strong and encouraging relationship between the integrity of a network derived from measures of cortical thickness and behavioral performances. Future research can benefit from this approach to stratify patient populations and predict potential recovery.

Additionally, we explored whether structural changes such as other neural (eg, synaptic plasticity) or nonneural factors (eg, altered properties of the vasculature) could also be captured by measures of rs-*f*MRI entropy. Our results replicated recently published results that showed a strong decrease of entropy in both hemispheres when patients were compared to controls [101]. This indicates a large-scale effect of brain lesion on the overall BOLD dynamic of the brain. Finally, the result between patients (connected and disconnected) did not survive the correction for multiple comparisons, suggesting that, although promising, Shannon entropy measures of BOLD may be too noisy of a measure to capture very fine microstructural events with high enough statistical power.

Previous reports indicated that *AnaCOM* suffers from lower specificity than VLSM (Rorden et al. [102]. *AnaCOM* compares performance of patients with that of controls, an approach that has previously been criticized [102]. In the context of our study, classic VLSM did not reveal any significant area involved with category fluency. In classic VLSM approaches, nonoverlapping lesions are competing for statistical significance, fundamentally assuming that a single region is responsible for the symptoms. In the present study, we followed Associationist principles [19, 18], assuming that several interconnected regions will contribute to the elaboration of the behavior. By comparing the performance between patients and a control population using *AnaCOM2*, several nonoverlapping regions can reach significance without competing for it. Hence, our results differ theoretically and methodologically from previous approaches. Perhaps more importantly, the network of disconnected areas revealed by *AnaCOM2* is typically considered as functionally engaged during fluency and categorization tasks in healthy controls.

Newer multivariate methods have also been shown to provide superior performance compared to traditional VLSM (ie, support vector regression lesion-symptom mapping) [7, 103]. For instance, such approaches have been used to model the statistical relationship between damaged voxels in order to reduce false positives. In the *disconnectome maps*, this relationship has been preestablished using an anatomical prior derived from tractography in healthy controls. Therefore, it is not recommended to use multivariate approaches with the *disconnectome maps*, as they might come into conflict with the prebuilt anatomical association between the voxels. Additionally, these approaches require a much larger database of patients than the current study. Future research that uses large lesion databases will be required to explore the effect of multivariate statistical analysis on *disconnectome maps*.

Multivariate approaches also elegantly demonstrated that false positives can be driven by the vascular architecture [7]. This is an important limitation concerning any voxel and vascular lesion symptom mapping. Here, the group of patients explored had stroke and surgical lesions. Although we cannot exclude the participation of the vascular architecture in the present findings, the heterogeneity of the lesions included in our analyses may have limited this factor. Additionally, the statistical interaction between vascular architecture and the *disconnectome map* results remain to be explored in large databases of lesions.

Methods used to estimate cortical thickness have previously been reported to perform poorly in peri-infarct regions, and the quality of the tissue segmentation may be particularly poor for stroke patients [79]. Here, we followed previously published recommendations for applying DiReCT [77] to the data from stroke patients; the lesion was masked out, the tissue segmentations were visually inspected, and manual boundary correction was performed when necessary (see Supplementary Fig. S2 for an example).

Finally, we applied our methods to the neural basis of category fluency as a proof of concept. The anatomy of category fluency should be, ideally, replicated in a larger sample of patients that includes adequate lesion coverage of the entire brain to provide a more comprehensive understanding of category fluency deficit after a brain lesion. While gathering such a large dataset of patients with brain lesions would have been impossible to achieve before, it might soon become possible thanks to collaborative initiatives such as the Enigma Consortium stroke recovery initiative [104, 105].

## Conclusion

Overall, using *BCBtoolkit*, researchers and clinicians can measure distant effects of brain lesions and associate these effects with neuropsychological outcomes. However, our methods require the manual delineation of lesion masks, automatization remaining a big challenge, especially on T1 images [105]. Taken together, these neuroimaging measures help discern the natural history of events that occur in the brain after a lesion, as well as assist in the localization of functions. These methods, gathered in the *BCBtoolkit*, are freely available as supplementary software [1, 107].

## Availability of supporting data

Patients’ lesions registered to the reference map MNI152 are available as supplementary material via the BCBlab website [106] and via the GigaScience database GigaDB [107]. However, we are not able to fully share the actual clinical sample data because sharing of the clinical raw data is not covered by the participants’ consent. A copy of the consent form as signed by the participants is available via GigaDB.

## Availability of supporting source code and requirements

- Project name: BCBtoolkit
- Project home page: http://toolkit.bcblab.com
- Operating system(s): Linux, MacOS
- Programming language: Java, Bash, R
- Other requirements: FSL, R, Python 2.7, Numpy
- License: BSD 3-Clause

An archival copy of the supporting source code is also available via GigaDB [107].

## Additional material

Figure S1: Step by step, hypotheses-driven analyses with BCBtoolkit.

Figure S2: Native T1, enantiomorphic deformation and derived cortical thickness of 3 representative patients.

Figure S3: Cortical thickness and Shannon entropy measures (mean with 95% confidence intervals) in patients with (dark gray) or without (light gray) disconnection for each of the disconnected areas. The green interval indicates performance in matched controls with 95% confidence intervals.

## Competing interests

The authors declare that they have no competing interests.

## Author contributions

C.F. implemented the methods inside the *BCBtoolkit*, performed the analyses, and wrote the manuscript. L.C. created the pipeline for the preprocessing of the resting state and for the functional correlation and revised the manuscript. S.K. conceived and helped to upgrade the statistical analyses. C.R. collected the neuroimaging data. M.U. and E.V recruited the participants; collected and built the database of patients; matched healthy controls, including the neuropsychological and neuroimaging data; and revised the manuscript. E.V. also participated in the conception of the lesion study and provided funding for the database acquisition. R.L. provided funding for the study and revised the manuscript. M.T.d.S. wrote the manuscript, provided funding, conceived and coordinated the study, and reviewed and collected neuroimaging data.

## Supplementary Material

GIGA-D-17-00149_Original-Submission.pdf

GIGA-D-17-00149_Revision-1.pdf

GIGA-D-17-00149_Revision-2.pdf

Response-to-Reviewer-Comments_Original-Submission.pdf

Response-to-Reviewer-Comments_Revision-1.pdf

Reviewer-1-Report_Original-Submission -- Aaron Boes 07 Aug 2017 Reviewed

Reviewer-1-Report_Revision-1 -- Aaron Boes 28 Nov 2017 Reviewed

Reviewer-2-Report_Original_Submission -- Muthuraman Muthuraman 15 Aug 2017 Reviewed

Reviewer-2-Report_Revision-1 -- Muthuraman Muthuraman 29 Nov 2017 Reviewed

Supplement Materials
