# Supplementary material for: Advanced lesion symptom mapping analyses and implementation as BCBtoolkit
Source: Gigascience. 2018 Feb 8;7(3):giy004. doi: 10.1093/gigascience/giy004 (PMC5863218; doi:10.1093/gigascience/giy004)
Supplement: GIGA-D-17-00149_Revision-1.pdf [file giy004_giga-d-17-00149_revision-1.pdf]

GigaScience

Advanced lesion symptom mapping analyses and implementation as BCBtoolkit

--Manuscript Draft--

**Manuscript Number:**	GIGA-D-17-00149R1
**Full Title:**	Advanced lesion symptom mapping analyses and implementation as BCBtoolkit
**Article Type:**	Technical Note
**Funding Information:**	Agence Nationale de la Recherche (ANR-09-RPDOC-004-01) — Dr. Emmanuelle Volle<br>Agence Nationale de la Recherche (ANR-13- JSV4-0001-01) — Dr. Michel Thiebaut de Schotten<br>Agence Nationale de la Recherche (ANR-10-IAIHU-06) — Not applicable<br>Fondation pour la Recherche Médicale — Pr. Richard Levy
**Abstract:**	Background: Patients with brain lesions provide a unique opportunity to understand the functioning of the human mind. However, even when focal, brain lesions have local and remote effects that impact functionally and structurally connected circuits. Similarly, function emerges from the interaction between brain areas rather than their sole activity. For instance, category fluency requires the association between executive, semantic and language production functions.<br>Findings: Here we provide, for the first time, a set of complementary solutions to measure the impact of a given lesion upon the neuronal circuits. Our methods, which were applied to 37 patients with a focal frontal brain lesion, revealed a large set of directly and indirectly disconnected brain regions that had significantly impacted category fluency performance. The directly disconnected regions corresponded to areas that are classically considered as functionally engaged in verbal fluency and categorization tasks. These regions were also organized into larger directly and indirectly disconnected functional networks, including the left ventral fronto-parietal network, whose cortical thickness correlated with performance on category fluency.<br>Conclusions: The combination of structural and functional connectivity together with cortical thickness estimates reveals the remote effects of brain lesions, provide for the identification of the affected networks and strengthen our understanding of their relationship with cognitive and behavioural measures. The methods presented are available and freely accessible in the BCBtoolkit as supplementary software (http://toolkit.bcblab.com).
**Corresponding Author:**	Michel Thiebaut de Schotten<br>CNRS Délégation Paris B<br>Paris, Île-de-France FRANCE
**Corresponding Author Secondary Information:**	
**Corresponding Author's Institution:**	CNRS Délégation Paris B
**Corresponding Author's Secondary Institution:**	
**First Author:**	Chris Foulon
**First Author Secondary Information:**	
**Order of Authors:**	Chris Foulon<br>Leonardo Cerliani<br>Serge Kinkingnéhun<br>Richard Levy<br>Charlotte Rosso<br>Marika Urbanski<br>Emmanuelle Volle<br>Michel Thiebaut de Schotten

Powered by Editorial Manager® and ProduXion Manager® from Aries Systems Corporation

**Order of Authors Secondary Information:**	
**Response to Reviewers:**	Changes have been highlighted in blue in the manuscript.<br><br>Reviewer #1: In this article Foulon et al present a novel multimodal suite of software for evaluation of focal brain lesions and their associated networks. They demonstrate the utility of their tools using an analysis of 37 patients with frontal lobe lesions and relate lesions to category fluency performance. 54 healthy comparison subjects are also included and all subjects were asked to name as many animals as they could within 60 seconds. 10 of the 54 subjects have DTI imaging.<br><br>Subjects had structural T1 images and 10 minutes of resting state data.<br><br>Overall I think this is a valuable contribution to the literature and to the lesion mapping community. The main strength in my opinion is the sheer number of analyses performed that all attempt to address various aspects of lesion-associated networks as it relates to category fluency performance. The fact that these analyses can be done by anyone using freely available software packages provided by the authors is important. The authors provide a well-written and well-referenced introductory overview of lesion mapping and diaschisis.<br><br>Thank you.<br><br>Some of the limitations of the article in its current form include the following:<br><br>While the category fluency analysis servers primary to illustrate the novel methods used and is valuable in that regard the sample size for this complex functional task seems underpowered. If the primary goal of the article was to definitively outline the neural basis of category fluency I'm not sure this sample would warrant a high impact journal.<br><br>We explored category fluency as a proof of concept. We now acknowledge that a larger dataset should be investigated in the discussion.<br>'Finally, we applied our methods to the neural basis of category fluency as a proof of concept. The anatomy of category fluency should be, ideally, replicated in a larger sample of patients including lesions involving the entire brain to provide a more comprehensive understanding of category fluency deficit after a brain lesion. While gathering such a large dataset of patients with brain lesions would have been impossible to achieve before, it might soon become possible thanks to collaborative initiatives such as the Enigma Consortium stroke recovery initiative (http://enigma.ini.usc.edu/ongoing/enigma-stroke-recovery/) (Liew et al., 2017).'<br><br>While the authors focus on lesion-associated networks it would still be useful to display a standard lesion overlap image and perform some type of VLSM on the lesions themselves. If lesion symptom mapping of the lesions themselves is not significant this doesn't necessarily hinder their conclusions and may actually support the need for lesion-associated network analyses.<br><br>Thank you for this suggestion. In the context of our study, classical VLSM did not reveal any significant area involved with category fluency. We added this information in the discussion.<br><br>Because there are so many novel analyses the methods section feels inadequate to me. It is difficult to follow exactly what analyses are being performed without reading carefully through the manuscript multiple times.<br><br>Apologies if this was unclear, we now produced a figure to help the reader to follow the analyses step by step, reorganised the result section so that it mirrors the method section exactly. We clarified the Anacom section and used a fixed terminology in the methods and results.<br><br>"In the following sections of the manuscript, the term clusters systematically refers to the result of the post-hoc Mann-Whitney comparison between disconnected patients

and healthy subjects that survived Bonferroni-Holm correction for multiple comparisons."
We also chose a fixed term for the three functional networks produced by the functional connectivity calculation followed by a PCA:
"In the following sections of the manuscript, the term factor-networks systematically refers to brain regions having a statistically significant relationship with the three components."

Whenever possible the authors should be more explicit about what is being used as seed regions for a given analysis (e.g. lesion masks or statistical maps from prior analysis) and when they are using data from controls versus the patients themselves. If space limitation is the problem perhaps a supplemental methods section would help.

We're sorry if this was unclear. As mentioned above we now use a fixed terminology to indicate exactly what is being used for the analyses.

Normalization - Uses a creative method of filling in the damaged tissue by copying the images from the healthy hemisphere opposite the lesion. This helps with the accuracy of registration into MNI space. One limitation of this approach is the requirement of manually tracing the lesion in both the native space and in MNI152 space, but this will be a valuable tool nonetheless.

Indeed, this is one of the limitations, which has now been included in the discussion:
"However, our methods require the manual delineation of lesion masks, automatization remaining a big challenge, especially on T1-images (Liew et al., 2017)."

White matter disconnection -
This tool takes the lesion mask and its overlap with standard regions of interest defined using a white matter atlas (Rojkova, 2016).

I wonder why the authors chose this particular atlas.

The main reason for this choice is that this atlas maps the main white matter tracts of the frontal lobes where are mainly located the lesions of our dataset.

The white matter tracts appear quite large and if I understand the methods the defined the presence of a tract by a probability of 50% or greater. When doing this for the corticospinal tract as a quality check there are voxels that appear to me to be entirely outside of where the corticospinal tract is.

The corticospinal tract actually originates from different part of the brain and does include motor areas primary somatosensory cortex and premotor areas. The overall extent of the tract of our atlas is comparable to post-mortem sections (Rademacher 2001).
Additionally, we now added the JHU atlas inside the tractotron.

I wonder if the authors could input more than one white matter atlas into their toolbox, such as other freely available atlases (e.g. JHU).

Thank you for the suggestion, we added the JHU inside the Tractotron.

I also think it would be beneficial to use the probability data from the white matter atlas to weight the lesion involvement in the tract. For example if a lesion hits the center of a tract it would carry a greater weight than hitting the periphery. This could be done by using a summation of each voxel's probability from each white matter tract as opposed to thresholding and binarizing the white matter tract.

As such the lesion load for each tract could be treated as a continuous variable and correlated to the behavior of interest.

This is good suggestion as mentioned in the introduction we provide here a simple example of the use of the methods. The neuroscientific community is more than welcome to use this material to explore disconnection differently.

Powered by Editorial Manager® and ProduXion Manager® from Aries Systems Corporation

Direct disconnection -
This analysis used the lesion masks as seed regions of interest. Each lesion mask was brought into the native space of 10 healthy subjects that had DTI imaging and tracts were produced. The resulting tracts were thresholded, binarized and statistics were performed to relate the tracts to category fluency using AnaCOM2, a package for lesion symptom mapping.

The authors note they binarized the tracts produced in Trackvis. What threshold was used and what is the justification for choosing it?

Apologies if this was unclear in the manuscript. We didn't use a threshold in trackvis, we binarized the fibre density map to indicate presence/absence of the connection rather than the number of streamlines at the individual level. Later on, the group maps are thresholded at >50% to restrict the analysis to fibres represented in more than half of the healthy control population. >50% correspond to an effect size >0.5, i.e. 50% of the variance explained corresponding to a large effect.
We now clarified the methods accordingly

Indirect disconnection -
The significant clusters resulting from the direct disconnection analysis were used as seed ROIs for a resting state functional connectivity analysis using the normative fcMRI data at the group level.

How was the median network calculated at the group level?

We clarified this in the methods

"The median network resulting from a seed contains, in each voxel, the median of functional connectivity across all the control subjects. Medians were chosen instead of average as they are less sensitive to outliers and are more representative of the group level data (Kenney 1939). The calculation of the functional connectivity was automatized and made available inside the Funcon tool as part of BCBtoolkit. Medians were calculated using the function fslmaths."

Additional details about this analysis would be helpful. Were the significant 'disconnection' sites from the direct disconnection analysis used to seed fcMRI analyses in the control cohort and then the resulting network strength was tested using the patient data? How was connected versus disconnected status determined in these groups?

Thank you for this remark. We omitted to mention this analysis in the method section. This has now been clarified as follows:

"Additionally, for each patient, we extracted the time course that corresponded to each factor-network. These time courses were subsequently correlated to the rest of the brain so as to extract seed-based factor-networks in each patient. FSLstats was employed to extract the strength of factor-networks functional connectivity and subsequently compare patients according to their disconnection status. Note that a patient disconnected in a factor-network is a patient who has a disconnection in at least one of the cluster that contributed significantly to the factor-network."

Structural Changes
Cortical thickness was assessed across entire networks as defined using the indirect disconnection analysis above.

It is not clear to me that there is any way to relate the cortical thinning to the lesion. For example it is possible that these patients have thinning in association with aging and poor vascular health and these factors contributed to category fluency deficits. Does thinning at these remote sites relate to lesion size?

In order to control for the effect of aging and lesion size the same analysis was repeated regressing out for these two parameters. We're happy to report that the result remained significant.

'The same analyses were repeated controlling for age and lesion size and confirmed the results for ventral fronto-parietal network seeded from the left MFg (Spearman Rho = .423; p = .01) , IPs (Rho = .538; p = .001) and left opercularis (Rho = .590 ± 0.341 ; p < .001) corresponded to a reduced performance in category fluency (Fig. 5). Additionally, a thinner cortical thickness in the left preSMA functional network (Rho = .439 ; p = .007) and a higher rs-fMRI entropy (Rho = – .420 ± 0.370 ; p = .019) in the mid cingulate gyrus functional network was associated with poorer performance in category fluency'

Shannon entropy as a structural measure could be better described.

Thank you for this. We now describe Shannon entropy more in details in the methods of the manuscript.

'Shannon entropy is an information theory derived measure that estimates signal complexity (Shannon, 1997 ; Gray, 2011). In the context of rs-fMRI, measure of entropy measure the local complexity of the Blood Oxygen Level Dependent (BOLD) signal as a surrogate of the complexity of the spontaneous neuronal activity (Ogawa et al., 1990 ; Biswal et al., 1995). Since cells that fire together wire together (Hebb, 1949), for each grey matter voxel Shannon entropy of rs-fMRI can be considered as a surrogate for the complexity of the connections within this voxel and between this voxel and the rest of the brain. Shannon entropy was extracted from the previously preprocessed rs-fMRI using the following formula: -sum(p*log(p)) where p indicates the probability of the intensity in the voxels (Tononi et al. 1998)'

Other comments:
The order of the methods does not match the results for structural changes and indirect disconnection.

Thanks we changed the order of the results to match.

The authors may consider adding larger datasets for the normative fcMRI and DTI analyses from the freely available human connectome project.

The is a good suggestion, unfortunately our normative datasets are matched for age, education and acquisition parameters. Additionally, we tested our methods in the context of category fluency, which is a language task, while our patients are french, and the HCP does not contain data with the particular test, we cannot use it to extend our dataset.

Reviewer #2: In this manuscript the authors demonstrate impact of a lesion on the functional and structural connections or disconnections in frontal lobe lesion patients using category fluency task. Additionally cortical thickness was used which provide a bridge between the cognitive and behaviour measures and the applied methods are available in an open source toolbox called bcbtoolkit.

Major comments:

1. White matter disconnection- Which two groups were compared using the Kruskal Wallis test is not clear?

Apologies if this was unclear. We now clarified the methods accordingly:

"Thereafter, we used AnaCOM2 available within the BCBtoolkit in order to identify the disconnections that are associated with a given deficit, i.e. connections that are critical for a given function. AnaCOM2 is comparable to AnaCOM (Kinkingnehun et al., 2007) but has been reprogrammed and optimised to work on any Linux or Macintosh operating systems.
Initially, AnaCOM is a cluster-based lesion symptom mapping approach, which identifies clusters of brain lesions that are associated with a given deficit, i.e. the regions that are critical for a given function. In the context of this paper, AnaCOM2

used disconnectome maps instead of lesion masks, to identify cluster of disconnection that are associated with category fluency deficits, i.e. the networks that are critical for a given function. Compared to standard VLSM (Bates et al., 2003), AnaCOM2 regroups voxels with the same distribution of neuropsychological scores into clusters of voxels. Then, for each cluster, AnaCOM2 will perform a Kruskal-Wallis test between patients with a disconnection, patients spared of disconnection and controls. Resulting p-values are Bonferroni-Holm corrected for multiple comparisons. Subsequently significant clusters (p-value < 0.05) are used to perform a post-hoc Mann-Whitney comparison between two subgroups of interest (i.e. disconnected patients and healthy subjects). Post-hoc results are Bonferroni-Holm corrected for multiple comparisons (statistical tests and corrections are computed using R language: R Core Team 2016, https://www.r-project.org).
Patients-controls comparison have been chosen as a first step in order to avoid drastic reduction of statistical power when two or more non-overlapping areas are responsible for patients reduced performance (Kinkingnehun et al., 2007). Non-parametric statistics have been chosen as it is fair to consider that some clusters will not show a Gaussian distribution. AnaCOM2 resulted in a statistical map that reveals, for each cluster, the significance of a deficit in patients undertaking a given task as compared to controls."

2. The authors have done several different analyses and it is very difficult to follow and does not look like hypothesis driven more exploratory analyses. For the reader would be very useful to have a pipeline figure to illustrate the various analyses steps.

Thank you for mentioning this we now indicate in the methods that all analyses were data drive and provide an outline of the analyses as supplementary figure 1

"The following sections of the manuscript are hypotheses driven and outlined in supplementary figure 1."

3. Connectome maps based on only 10 normal participants for the DTI and comparing them to n=37 patient cohort is not very trivial? The covariance in the tracts was based comparing present or not present could also be artifacts?

Thank you for pointing at this. We kept the normal participants' dataset as small as possible to avoid increasing drastically the size of the BCBtoolkit.
10 participants are usually considered as the minimum when it comes to build templates.
However, the optimal number of participants has never been assessed in the context of the disconnectome maps. Here we explored this question gathering diffusion data from the 54 healthy participants explored behaviourally in our study. This material has now been added as supplementary material with the manuscript.

"The optimal number of participants was calculated disconnectome maps from separate paired populations of equal gender distribution. This approach was repeated for groups consisting of 4, 6, 8, 10, 12, 14, 16, 18 and 20 subjects. Squared spatial Pearson's correlations between each pair (i.e. square of fslcc from FSL) was employed to calculate the percentage of shared variance (i.e. the similarity). Figure 1a indicates a steep increase of shared variance between disconnectome maps produced from 4 to 10 participants followed by a slower increase from 10 to 20 participants. This result indicates that, using the disconnectome, 10 subjects are sufficient to produce a good enough disconnectome map that matches the overall population (above 70% of shared variance). A larger dataset (n = 36) can be downloaded on our website (http://www.bcblab.com/opendata). Additionally, HCP 7T data (n = 166) have been prepared for the disconnectome and are available on demand to the authors (hd.chrisfoulon@gmail.com or michel.thiebaut@gmail.com)."
We also measured whether the shape of the disconnectome changes over age. We assessed this question by producing disconnectome maps for each decade. We quantified similarities using squared spatial Pearson's for the 21-30-year-old maps and the maps for the other decades. The result indicates that disconnectome maps show a very high anatomical similarity between decades and no decrease of this similarity with age. Hence disconnectome maps in our sample did not show any age-related changes.

	4. The category fluency task scores was a simple integer value over the whole paradigm which was then correlated is not clear?<br><br>The performance value corresponds to the number of animals the subjects were able to produce in 120 seconds<br><br>5. The authors bring in functional connectivity as support for the dysfunctional and disconnected area will impact the indirectly connected areas. Better approach would be to prove this by causal approaches like effective connectivity (direction of the connection) instead of functional connectivity which is only correlation.<br><br>The effective connectivity is beyond the scope of this study.<br><br>6. Some parts mainly the methods and the results are difficult to follow english can be improved like for ex: in discussion „consequences upon a patients' "<br><br>Thank you for pointing at this English mistake. The manuscript has now been read and edited by a native speaker.<br><br>Minor comments:<br><br>Full forms of acronyms at first mention: What is RR?<br>Thanks, we now clarified this point in the text.
**Additional Information:**	
**Question**	**Response**
Are you submitting this manuscript to a special series or article collection?	No
**Experimental design and statistics**<br><br>Full details of the experimental design and statistical methods used should be given in the Methods section, as detailed in our Minimum Standards Reporting Checklist. Information essential to interpreting the data presented should be made available in the figure legends.<br><br>Have you included all the information requested in your manuscript?	Yes
**Resources**<br><br>A description of all resources used, including antibodies, cell lines, animals and software tools, with enough information to allow them to be uniquely identified, should be included in the Methods section. Authors are strongly encouraged to cite Research Resource Identifiers (RRIDs) for antibodies, model organisms and tools, where possible.<br><br>Have you included the information requested as detailed in our Minimum Standards Reporting Checklist?	Yes

**Availability of data and materials**<br><br>All datasets and code on which the conclusions of the paper rely must be either included in your submission or deposited in publicly available repositories (where available and ethically appropriate), referencing such data using a unique identifier in the references and in the "Availability of Data and Materials" section of your manuscript.<br><br>Have you have met the above requirement as detailed in our Minimum Standards Reporting Checklist?	Yes

Powered by Editorial Manager® and ProduXion Manager® from Aries Systems Corporation

Manuscript Click here to download Manuscript foulonetalv17.docx

Advanced lesion symptom mapping analyses and implementation as *BCBtoolkit*

Foulon C[a,b,c*], Cerliani L[a,b,c], Kinkingnéhun S[a], Levy R[b], Rosso C[c,d,e], Urbanski M[a,b,f], Volle E[a,b,c], Thiebaut de Schotten M[a,b,c*]

[a] Brain Connectivity and Behaviour Group, Sorbonne Universities, Paris France.

[b] Frontlab, Institut du Cerveau et de la Moelle épinière (ICM), UPMC UMRS 1127, Inserm U 1127, CNRS UMR 7225, Paris, France.

[c] Centre de Neuroimagerie de Recherche CENIR, Groupe Hospitalier Pitié-Salpêtrière, Paris, France.

[d] Abnormal Movements and Basal Ganglia team, Inserm U 1127, CNRS UMR 7225, Sorbonne Universities, UPMC Univ Paris 06, Institut du Cerveau et de la Moelle épinière, ICM, Paris, France

[e] APHP, Urgences Cérébro-Vasculaires, Groupe Hospitalier Pitié-Salpêtrière, Paris, France.

[f] Medicine and Rehabilitation Department, Hôpitaux de Saint-Maurice, Saint-Maurice, France.

* Corresponding authors hd.chrisfoulon@gmail.com and michel.thiebaut@gmail.com

Competing interests:

The authors declare that they have no competing interests

Abstract

Background: Patients with brain lesions provide a unique opportunity to understand the functioning of the human mind. However, even when focal, brain lesions have local and remote effects that impact functionally and structurally connected circuits. Similarly, function emerges from the interaction between brain areas rather than their sole activity. For instance, category fluency requires the association between executive, semantic and language production functions.

Findings: Here we provide, for the first time, a set of complementary solutions to measure the impact of a given lesion upon the neuronal circuits. Our methods, which were applied to 37 patients with a focal frontal brain lesion, revealed a large set of directly and indirectly disconnected brain regions that had significantly impacted category fluency performance. The directly disconnected regions corresponded to areas that are classically considered as functionally engaged in verbal fluency and categorization tasks. These regions were also organized into larger directly and indirectly disconnected functional networks, including the left ventral fronto-parietal network, whose cortical thickness correlated with performance on category fluency.

Conclusions: The combination of structural and functional connectivity together with cortical thickness estimates reveals the remote effects of brain lesions, provide for the identification of the affected networks and strengthen our understanding of their relationship with cognitive and behavioural measures. The methods presented are available and freely accessible in the *BCBtoolkit* as supplementary software (http://toolkit.bcblab.com).

Keywords

Brain, MRI, Lesion, Statistics, Software, Open source, Connectivity, Disconnection, Behaviour

Recent advances in neuroimaging techniques, allowed for the further examination of the structural and the functional organization of the human brain. While diffusion weighted imaging (DWI) tractography [1] depicts how brain areas are connected together, functional magnetic resonance imaging (*f*MRI) measures the activity within and interaction between brain areas in the elaboration of functions [2]. These methods have been successfully applied to the healthy human brain, however, they remain underused in patients with brain lesions.

Patients with brain lesions provide a unique opportunity to understand the functioning of the human mind. Lesion symptom mapping analyses traditionally assume that visible and directly damaged areas are responsible for a patient's symptoms [3-6]. Following this logic, the areas that are the most frequently damaged by the lesion are considered as the neuronal substrate for the function. Previous studies employing this method have pinpointed critical areas dedicated to, for example, language production [7], comprehension [8], spatial awareness [9-12] and other high-level cognitive functions [13-16]. However, anatomical disconnections between regions are also important considerations for the exploration of cognitive deficit [17, 18]. The dysfunction of distant areas that are connected to the lesioned tissue has also been reported in *f*MRI studies. They have shown that the networks are disrupted even by distant lesions through disconnection and diaschisis mechanisms [19-21].

Non-local effects of lesions have previously been explored using various forms of atlas-based analyses of tract damage [22-31], lesion-driven tractography [31-33], disconnectome-mapping [34-38] and lesion-driven resting state *f*MRI (rs-*f*MRI) connectivity [33, 39]. However, determining what these methods actually measure and identifying how to properly combine them are not always fully clear to the scientific community. Furthermore, there is an extremely limited availability of free, open-source software that applies methods to measure the non-local effects of lesions. These resources and scientific tools remain very much inaccessible and present a potential threat to reproducible science [40].

Disconnections and diaschisis can have an impact upon distant regions in several respects through maladaptive responses and pathological spread [41]. When disconnected from its inputs and outputs, a region can no longer contribute to the elaboration of the supported function. This phenomenon is called diaschisis [19, 20, 42]. Once deprived from its inputs

and/or outputs, transneuronal degeneration in the region will occur [41], dendrites and synapses density will decrease in number, myelin content will be altered and neurons will reduce in size or die through a mechanism called apoptosis, a programmed cell death [43-45]. Hence, a white matter disconnection leads to both functional and anatomical changes that extend well beyond the visible damage. New approaches are therefore required to capture the long-range effects that follow brain disconnections. For instance, cortical thickness [e.g. 46] and other volumetric [e.g. voxel based morphometry 47] analyses have been previously used to study the structural changes associated with brain lesions, but have not been applied in the context of brain disconnection.

In response to this need, we provide here a set of complementary solutions to measure both the circuit, and the subsequent changes within the circuit that is caused by a lesion. We applied these methods to 37 patients with a focal brain lesion following a stroke or a surgical resection. We first assessed the risk of disconnection in well-known white matter tracts and tested their relationship with category fluency performance. Category fluency is an appropriate test to explore disconnection since it requires the association between executive, semantic and language production functions [48, 49]. We then developed a tractography-based approach in order to produce maps of the areas that are directly disconnected by the lesion and tested their relationship with category fluency performance. We additionally calculated the rs-*f*MRI connectivity of these areas to reveal the whole network of directly and indirectly connected regions that participate in category fluency. Finally, we explored potential microstructural changes in the latter disconnected regions, by estimating neuronal loss or local connectivity degeneration derived from MR-based measures of cortical thickness and resting state *f*MRI entropy.

Methods

Participants and Category fluency task

Thirty-seven right-handed patients (French-native speakers; 19 females; mean age 48 ±14.2 years, age ranging from 23 to 75 years) who presented with a frontal lobe lesion at the chronic stage (> 3 months) were included in this study (see table 1 for demographics). These patients were recruited from the stroke unit and the neuroradiology department at Salpêtrière

Hospital, the neurological unit at Saint-Antoine Hospital and the neuroradiology department at Lariboisière Hospital in Paris. Patients with a history of psychiatric or neurological disease, drug abuse, or MRI contraindications were not included. Additionally, we gathered behavioural data from 54 healthy participants (French-native speakers; 27 females; mean age 45.8 ±14.4 years, age ranging from 22 to 71 years) in order to constitute a normative group.

All participants performed a category fluency task [50] in French. They were instructed to enumerate as many animals as possible during a timed period of 120 seconds. The results were recorded by a clinical neuropsychologist (M.U.). Repetition and declination of the same animal were not taken into account in the final category fluency score.

The experiment was approved by the local ethics committee; all participants provided written informed consent in accordance to the Declaration of Helsinki. Participants also received a small indemnity for their participation.

Magnetic resonance imaging

An axial three-dimensional magnetization prepared rapid gradient echo (MPRAGE) dataset covering the whole head was acquired for each participant (176 slices, voxel resolution = 1 × 1 × 1 mm, echo time = 3 msec, repetition time = 2300 msec, flip angle = 9°).

Additionally, the same participants underwent an *f*MRI session of resting state. During the resting state session, participants were instructed to relax, keep their eyes closed but to avoid falling asleep. Functional images were obtained using T2-weighted echo-planar imaging (EPI) with blood oxygenation level-dependent contrast using SENSE imaging an echo time of 26 msec and a repetition time of 3000 msec. Each dataset comprised 32 axial slices acquired continuously in ascending order covering the entire cerebrum with a voxel resolution of 2 x 2 x 3 mm. 200 volumes were acquired using these parameters for a total acquisition time of 10 minutes.

Finally, diffusion weighted imaging was also acquired for 54 participants of the normative group (French-native speakers; 27 females; mean age 45.8 ±14.4 years, age ranging from 22 to 71 years) and consisted in a total of 70 near-axial slices acquired using a fully optimised acquisition sequence for the tractography of diffusion-weighted imaging (DWI), which provided isotropic (2 × 2 × 2 mm) resolution and coverage of the whole head with a posterior-anterior phase of acquisition. The acquisition was peripherally-gated to the cardiac

cycle [51] with an echo time = 85 msec. We used a repetition time equivalent to 24 RR (i.e. interval of time between two heart beat waves). At each slice location, 6 images were acquired with no diffusion gradient applied. Additionally, 60 diffusion-weighted images were acquired, in which gradient directions were uniformly distributed on the hemisphere with electrostatic repulsion. The diffusion weighting was equal to a b-value of 1500 sec mm^{-2}.

Stereotaxic space registration

As spatial normalisation can be affected by the presence of a brain lesion, additional processing was required before calculating the normalisation. To this purpose, the first step was to produce an enantiomorphic filling of the damaged area [52]. Each patient's lesion (or signal abnormalities due to the lesion) were manually segmented (using FSLview; http://fsl.fmrib.ox.ac.uk) and replaced symmetrically by the healthy tissue of the contralateral hemisphere. Enantiomorphic T1 images were fed into FAST [53] for estimation of the bias field and subsequent correction of radiofrequency field inhomogeneity. This improved the quality of the automated skull stripping performed using bet [54] and the registration to the MNI152 using affine and diffeomorphic deformations [55]. The original T1 images (non enantiomorphic) were registered to the MNI152 space using the same affine and diffeomorphic deformations as calculated above. Subsequently, lesions were segmented again in the MNI152 space under the supervision of an expert neurologist (E.V.). This method has been made freely available as the tool *normalisation* as part of *BCBtoolkit* (http://toolkit.bcblab.com).

The following sections of the manuscript are hypotheses-driven and outlined in supplementary figure 1.

White matter tracts disconnection

Each patient's lesion was compared with an atlas of white matter tracts [56], indicating for each voxel, the probability of finding a white matter tract such as the arcuate fasciculus, the frontal aslant tract or the uncinate fasciculus in the MNI152 coordinate system. We considered a tract to be involved when the likelihood of a tract being present in a given voxel was estimated above 50% [22]. This method is freely available as *tractotron* in *BCBtoolkit* (http://toolkit.bcblab.com). We focused on frontal lobe tracts with a potential effect on

executive, semantic and language functions since all of the patients had a frontal lesion. These tracts included the cingulum, the frontal aslant and the frontal superior and inferior longitudinal tracts for the executive functions [57], the uncinate and the inferior fronto-occipital fasciculi for the semantic access [58, 59] and the anterior and long segment of the arcuate fasciculi for the phonemic system [60, 61]. A Kruskall-Wallis test was employed to compare performance on the category fluency test for each tract between both preserved and disconnected patients and control participants. Subsequently, for each significant tract between patients, Mann-Whitney post-hoc comparisons were performed (**Fig.1)**.

Direct disconnection of brain areas: structural connectivity network

This approach employed the diffusion weighted imaging datasets of 10 participants in the normative group to track fibres passing through each lesion.

For each participant, tractography was estimated as indicated in [62].

Patients' lesions in the MNI152 space were registered to each control native space using affine and diffeomorphic deformations [55], and subsequently, used as seed for the tractography in Trackvis (http://trackvis.org). Tractography from the lesions were transformed in visitation maps [63, 64], binarized and brought to the MNI152 using the inverse of precedent deformations. Finally, we produced a percentage overlap map by summing at each point in the MNI space the normalized visitation map of each healthy subject. Hence, in the resulting *disconnectome map*, the value in each voxel took into account the inter-individual variability of tract reconstructions in controls, and indicated a probability of disconnection from 50 to 100% for a given lesion (i.e. thus explaining more than 50% of the variance in disconnection and corresponding to a large effect size). This procedure was repeated for all lesions, allowing the construction of a *disconnectome map* for each patient/lesion. These steps were automatized in the tool *disconnectome map* as part of the *BCBtoolkit*. Note that sample size and age effects were carefully explored and reported in the supplementary material. Overall, 10 subjects are sufficient to produce a good enough *disconnectome map* that matches the overall population (more than 70% of shared variance). We also demonstrate in the supplementary material that *disconnectome maps* show a very high anatomical similarity between decades and no decrease of this similarity with age.

Thereafter, we used *AnaCOM2* available within the *BCBtoolkit* in order to identify the

disconnections that are associated with a given deficit, i.e. connections that are critical for a given function. *AnaCOM2* is comparable to *AnaCOM* [65] but has been reprogrammed and optimised to work on any Linux or Macintosh operating systems.

Initially, *AnaCOM* is a cluster-based lesion symptom mapping approach, which identifies clusters of brain lesions that are associated with a given deficit, i.e. the regions that are critical for a given function. In the context of this paper, *AnaCOM2* used *disconnectome maps* instead of lesion masks, to identify clusters of disconnection that are associated with category fluency deficits, i.e. the connections that are critical for a given function. Compared to standard VLSM [7], *AnaCOM2* regroups voxels with the same distribution of neuropsychological scores into clusters of voxels. Then, for each cluster above $8mm^3$, *AnaCOM2* will perform a Kruskal-Wallis test between patients with a disconnection, patients spared of disconnection and controls. Resulting p-values are Bonferroni-Holm corrected for multiple comparisons. Subsequently, significant clusters (p-value < 0.05) are used to perform a post-hoc Mann-Whitney comparison between two subgroups of interest (i.e. disconnected patients and healthy subjects). Post-hoc results are Bonferroni-Holm corrected for multiple comparisons (statistical tests and corrections are computed using R language: R Core Team 2016, https://www.r-project.org).

Patients-controls comparisons have been chosen as a first step in order to avoid drastic reduction of statistical power when two or more non-overlapping areas are responsible for patients reduced performance [65]. Non-parametric statistics have been chosen, as it is fair to consider that some clusters will not show a Gaussian distribution. *AnaCOM2* resulted in a statistical map that reveals, for each cluster, the significance of a deficit in patients undertaking a given task as compared to controls.

In the following sections of the manuscript, the term clusters systematically refers to the result of the post-hoc Mann-Whitney comparison between disconnected patients and healthy subjects that survived Bonferroni-Holm correction for multiple comparisons.

fMRI Meta-analyses

A method described by Yarkoni et al. [66] (http ://neurosynth.org) was used to identify the functional networks involved in category fluency. We searched for brain regions that are consistently activated in studies that load highly on 2 features: “fluency” (120 studies, 4214 activations) and “category” (287 studies, 10179 activations). The results were superimposed

on the 3D reconstruction of the MNI152 images (**Fig. 3**).

Indirect disconnection of brain areas: functional connectivity network

Rs-*f*MRI images were first motion corrected using MCFLIRT [67], then corrected for slice timing, smoothed with a full half width maximum equal to 1.5 times the largest voxel dimension and finally filtered for low temporal frequencies using a gaussian-weighted local fit to a straight line. These steps are available in Feat as part of FSL package [68].

Rs-*f*MRI images were linearly registered to the enantiomorphic T1 images, and subsequently to the MNI152 template (2mm) using affine transformations. Confounding signals were discarded from rs-*f*MRI by regressing out a confound matrix from the functional data. The confound matrix included the estimated motion parameters obtained from the previously performed motion correction, the first eigenvariate of the white matter and cerebrospinal fluid (CSF) as well as their first derivative. Eigenvariates can easily be extracted using fslmeants combined with the --eig option. White matter and CSF eigenvariates were extracted using masks based on the T1 derived 3-classes segmentation thresholded to a probability value of 0.9, registered to the rs-*f*MRI images and binarized. Finally, the first derivative of the motion parameters, white matter and CSF signal was calculated by linear convolution between their time course and a [-1 0 1] vector.

For each control participant, we extracted the time course that corresponded to each significant cluster which was identified by the statistical analyses of the *disconnectome maps*. These time courses were subsequently correlated to the rest of the brain so as to extract seed-based resting-state networks. In order to obtain the most representative networks at the group level, for each seed-based resting-state network, we calculated the median network across the group. The median network resulting from a seed contains, in each voxel, the median of functional connectivity across all the control subjects. Medians were chosen instead of average as they are less sensitive to outliers and are more representative of the group level data [69]. The calculation of the functional connectivity was automatized and made available inside the *funcon* tool as part of *BCBtoolkit*. Medians were calculated using the function fslmaths.

Visual inspection revealed that several of these resting state networks shared a very similar distribution of activations. Therefore, an ‘activation’ matrix was derived from the seed-based

resting-state networks. This matrix consisted of columns that indicated each seed-based resting-state network, and rows that represented the level of activation for each voxel in the cortex. This 'activation' matrix was entered into a principal component analysis in SPSS (SPSS, Chicago, IL) using a covariance matrix and varimax rotation (with a maximum of 50 iterations for convergence), in order to estimate the number of principal components to extract for each function. Components were plotted according to their eigenvalue (y) (Lower left panel in **Fig. 4**) and we applied a scree test to separate the principal from residual components. This analysis revealed that three factors were enough to explain 82% of the variance of the calculated seed-based resting-state networks. Finally, brain regions having a statistically significant relationship with the three components (i.e. factor-networks) were detected using a linear regression with 5.000 permutations, in which the eigenvalues of the three components represented the independent variable and the seed-based resting-state networks the dependent variable. Results were Family Wise Error (FWE) corrected for multiple comparisons, and projected onto the average 3D rendering of the MNI152 template in the top panel of **Fig. 4**. In the following sections of the manuscript, the term factor-networks systematically refers to brain regions having a statistically significant relationship with the three components.

Additionally, for each patient, we extracted the time course that corresponded to each factor-network. These time courses were subsequently correlated to the rest of the brain so as to extract seed-based factor-networks in each patient. FSLstats was employed to extract the strength of factor-networks functional connectivity and subsequently, to compare patients according to their disconnection status. Note that a patient disconnected in a factor-network is a patient who has a disconnection in at least one of the cluster that contributed significantly to the factor-network.

Structural changes in disconnected regions

A distant lesion can affect cortical macro and microstructure remotely. Conscious of this, we attempted to estimate these structural changes and their relationship with category fluency within each functional factor-network. To this aim, we explored the properties of each functional network using two complementary measures: T1w-based cortical thickness to identify fine local volumetric changes and the Shannon entropy of rs-*f*MRI as a surrogate for

the local complexity of the neural networks [70]. Each original functional network seeded from each cluster was thresholded and binarized at r>0.3 and used as a mask to extract cortical thickness and entropy. Patients' lesions were masked out for these analyses.

For the cortical thickness, a registration-based method (Diffeomorphic Registration based Cortical Thickness, DiReCT) was employed [71] from the T1-weighted imaging dataset. The first step as for the *normalisation* was to produce an enantiomorphic filling of the damaged area in order to avoid the analysis to be contaminated by the lesioned tissue. The second step of this method consisted in creating two two-voxel thick sheets, one laying just between the grey matter and the white matter and the second laying between the grey matter and the CSF. Then, the grey/white interface was expanded to the grey/CSF interface using diffeomorphic deformation estimated with ANTs. The registration produced a correspondence field that allows an estimate of the distance between the grey/white and the grey/CSF interfaces, and thus corresponded to an estimation of cortical thickness. Voxels belonging to the lesion were subsequently removed from the cortical thickness maps (see **supplementary figure 2**). This approach has good scan-rescan repeatability and good neurobiological validity as it can predict with a high statistical power the age and gender of the participants [72] as well as atrophy following brain lesions [73]. Note that the striatum and the thalamus were excluded from the cortical thickness analysis since they do not have a cortical ribbon.

Shannon entropy is an information theory derived measure that estimates signal complexity [74, 75]. In the context of rs-*f*MRI, the entropy measures the local complexity of the Blood Oxygen Level Dependent (BOLD) signal as a surrogate of the complexity of the spontaneous neuronal activity [76, 77]. Since “cells that fire together wire together” [78], for each grey matter voxel Shannon entropy of rs-fMRI can be considered as a surrogate for the complexity of the connections within this voxel and between this voxel and the rest of the brain. Shannon entropy was extracted from the previously preprocessed rs-*f*MRI using the following formula: -sum(p*log(p)) where p indicates the probability of the intensity in the voxel [70].

FSLstats was employed to extract the average cortical thickness and resting state *f*MRI entropy for each cluster and factor-network. Statistical analysis was performed using SPSS software (SPSS, Chicago, IL). In our analysis, Gaussian distribution of the data was not confirmed for the cortical thickness and the entropy measures using the Shapiro–Wilk test. Therefore, non-parametric statistics were chosen to compare cortical thickness and entropy levels between patients disconnected, spared and controls in each cluster and factor network.

Additionally, bivariate Spearman rank correlation coefficient analyses were performed between the cortical thickness or entropy measurement of each functional network and each patient's category fluency performance. Correlation significant at $p < 0.0041$ survives Bonferroni correction for multiple comparisons (12 networks).

Data availability

Patients' lesions registered to the MNI152 were made available as supplementary material available at the following link http://opendata.bcblab.com. However, we were not able to fully share the actual clinical sample data due to consent issues.

Results

White matter tracts disconnection

Patients' lesions were compared to an atlas of white matter connections in order to identify the probability of tract disconnections [56]. A Kruskall-Wallis test indicated that for each tract, patients (i.e. connected and disconnected) and control participants showed a significantly different performance on the category fluency test (all $p < 0.001$, full statistics reported in **Table 2**). Between patients, post-hoc comparisons revealed that disconnections of the left frontal aslant (U = 90.0 ; p = 0. 0389), frontal inferior longitudinal (U = 69.0 ; p = 0. 0216) and frontal superior longitudinal (U = 75.0 ; p = 0. 0352) tracts, the anterior (U = 28.5 ; p = 0. 0116) and long segment (U = 31.5 ; p = 0.0059) of the arcuate fasciculus were associated with a poorer performance in category fluency (**Fig. 1**). However, these post-hoc comparisons did not survive Bonferroni-Holm correction for multiple comparisons.

These results indicate that poor performance measured in patients with brain damage can be associated to some extent with white matter tract disconnections.

Direct disconnection of brain areas: structural connectivity network

As different white matter atlases exist for the interpretation of the white matter tract

disconnection [79], and atlas-based approaches cannot assess the disconnection of the subportion of tracts nor the involvement of multiple tracts by a lesion, data driven maps of disconnection or 'disconnectomes' were produced. Using tractography in a group of 10 healthy controls, the registered lesions were used as a seed to reveal white matter tracts that passed through the injured area so as to produce maps of disconnections, later referred to as *disconnectome maps*. Category fluency scores were attributed to each patient's *disconnectome map* (see Fig. 2a). A Kruskall-Wallis test indicated that, for several clusters, patients (i.e. connected and disconnected) and control participants showed a significantly different performance on the category fluency test (all $p < 0.001$, full statistics reported in **Table 3**).

Results were further statistically assessed using Mann-Whitney post-hoc comparisons in order to identify areas that, when deafferented due to a disconnection mechanism, lead to a significant decrease in performance in category fluency when compared to controls.

The following results are Bonferroni-Holm corrected for multiple comparisons. Main cortical areas in the left hemisphere included the pre-supplementary motor area (PreSMA; Cluster size = 1449; Mann Whitney U = 88.5 ; p = 0.025), the anterior portion of the intraparietal sulcus (Cluster size = 1143; U = 18 ; p = 0.030), anterior (Cluster size = 837; U = 304 ; p = 0.025) and the middle (Cluster size = 898; U = 95.5 ; p = 0.014) cingulate gyrus, the middle frontal gyrus (MFg, Cluster size = 829; U = 81.5 ; p = 0.005), the pars opercularis of the inferior frontal gyrus (Cluster size = 5314; U = 16 ; p = 0.025).

In the right hemisphere, the preSMA (Cluster size = 1050; U = 50.5 ; p = 0.014), the MFg (Cluster size = 552; U = 54 ; p = 0.018), the anterior (Cluster size = 572; U = 44.5 ; p = 0.009) and the middle (Cluster size = 817; U = 317 ; p = 0.041) cingulate gyrus were also involved (**Fig. 2b**)

Subcortical areas in the left hemisphere involved the caudate, the putamen and several ventral thalamic nuclei including the ventral anterior (VA), the ventrolateral anterior (VLa) and the ventrolateral posterior (VLp) as a part of the same cluster (Cluster size = 5314; U = 16 ; p = 0.025)

In the right hemisphere, the striatum (Cluster size = 527; U = 310 ; p = 0.031), and the ventral thalamic nuclei (Cluster size = 935; U = 202.0 ; p = 0.025) were also involved (**Fig. 2b**).

Additionally, between patients (i.e. connected and disconnected, uncorrected for multiple comparisons) comparisons confirmed the critical involvement of the preSMA (U = 212, p = 0.0456) the MFg (U = 237, p = 0.01), the pars opercularis (U = 179, p = 0.004) and the intra-parietal sulcus (IPs, U = 172, p = 0.01) in the left hemisphere. The preSMA (U = 208; p = 0.01) and the MFg (U = 196; p = 0.038) were also involved in the right hemisphere (**Fig. 2c)**.

Full statistics are reported in **Table 3**

fMRI Meta-analyses

We further examined whether the disconnected areas in patients with poor performance are functionally engaged in tasks related to fluency and categorization using a meta-analysis approach (http://neurosynth.org) [66].

The result indicates that disconnected areas reported as significantly contributing to category fluency performance in patients are classically activated by functional MRI tasks requiring either fluency or categorization in healthy controls (**Fig. 3**).

Indirect disconnection of brain areas

As the *disconnectome mapping* method cannot measure the indirect disconnection produced by a lesion (i.e. it fails to measure the disconnection in a region that is not directly, anatomically connected to a damaged area, but that nonetheless remains a part of the same large network of functionally connected areas), we therefore employed functional connectivity in healthy controls. This allowed us to reveal the entire network of regions that are functionally connected to the areas that were reported as contributing significantly to the category fluency performance when directly disconnected. When compared to tractography, functional connectivity has the added advantage of revealing the areas that contribute to the network through both direct, as well as indirect, structural connections.

Principal component analysis indicated that the significant areas contributing to category fluency performance belonged to 3 main functional networks (i.e. factor networks) (**Fig. 4**), which accounted for more than 80% of the total variance of the functional connectivity results.

The left cingulate clusters (anterior and middle), the right anterior cingulate, the middle frontal gyrus, the thalamus, and the operculum all belonged to the cingulo-opercular network [CO,80] including also the right preSMA, posterior cingulate and the rostral portion of the middle frontal gyrus.

The middle of the cingulate gyrus and the striatum in the right hemisphere both belonged to a cortico-striatal network [CS,81] involving the right thalamus and striatum.

Finally, the left MFg, preSMA, IPs, the pars opercularis, the thalamus and the striatum were all involved in a larger, left ventral fronto-parietal network, which also included other areas such as the right preSMA, the frontal eye field and the temporo-parietal junction [VFP,82].

Additional analyses investigated the differences in the functional connectivity of these factor-networks relative to the disconnected status of areas involved in category fluency. Between patients (i.e. connected and disconnected) comparisons revealed significantly lower functional connectivity in the left VFP network ($U = 54.0$, $p = 0.006$) and in the CS network ($U = 63.0$, $p = 0.027$) when anatomically disconnected. The CO network, however, did not show any significant difference ($U = 40.0$, $p = 0.213$). Overall, the strength of the functional connectivity for each patient did not correlate significantly with the fluency performance.

Structural changes in disconnected regions

Additional exploratory analyses investigated structural changes related to the disconnections. We estimated these changes using two complementary measures: T1w-based cortical thickness to identify fine local volumetric changes and the Shannon entropy of rs-*f*MRI as a surrogate for the local complexity of the neural networks [70].

When compared to controls, patients showed a reduced cortical thickness in the left pars opercularis ($H = 13$; $p = 0.0012$), the MFg ($H = 8$; $p = 0.0143$), the preSMA ($H = 8$; $p = 0.0224$), the IPs ($H = 9$; $p = 0.0131$) and the right anterior ($H = 7$; $p = 0.0296$) and middle

cingulate gyrus (H = 23 ; p = 0.000). When compared to patients with no disconnection, solely the right middle cingulate gyrus survived the Bonferroni-Holm correction for multiple comparisons (U = 67; p = 0.004). When compared to controls, disconnected patients showed reduced entropy for all regions (all $p < 0.05$, except for the right middle frontal gyrus). However, when compared to patients with no disconnection, none of the comparisons survived the Bonferroni-Holm correction for multiple comparisons. Uncorrected p values are reported as an indication in **table 4, and bar chart in supplementary figure 3.**

None of these measures correlated significantly with the fluency performance.

In order to further assess the integrity of the whole network of regions that were functionally connected to the areas reported as having significantly contributed to the category fluency performance, we also extracted the cortical thickness and entropy from the regions that were functionally connected to the disconnected areas. Correlation analyses indicated that a thinner cortex in the ventral fronto-parietal network seeded from the left MFg (Spearman Rho = .464 ± 0.341; p = .004) , IPs (Rho = .475 ± 0.341 ; p = .003) and left oper./striatum/thalamus (Rho = .512 ± 0.341 ; p = .001) corresponded to a reduced performance in category fluency (**Fig. 5**). Additionally, a thinner cortical thickness in the left preSMA functional network (Rho = .376 ± 0.341 ; p = .024) and a higher rs-*f*MRI entropy (Rho = – .420 ± 0.370 ; p = .019) in the mid cingulate gyrus functional network was associated with poorer performance in category fluency. These two last results, however, did not survive Bonferroni-Holm correction for multiple comparisons.

The same analyses were repeated controlling for age and lesion size and confirmed the results for ventral fronto-parietal network seeded from the left MFg (Spearman Rho = .423; p = .01) , IPs (Rho = .538; p = .001) and left opercularis (Rho = .590 ± 0.341 ; p < .001) corresponded to a reduced performance in category fluency (**Fig. 5**). Additionally, a thinner cortical thickness in the left preSMA functional network (Rho = .439 ; p = .007) and a higher rs-*f*MRI entropy (Rho = – .420 ± 0.370 ; p = .019) in the mid cingulate gyrus functional network was associated with poorer performance in category fluency

Discussion

A large set of complementary methods can capture the impact of lesions on distant regions and expose the subsequent consequences upon patients' neuropsychological performance. Several of these methods are built directly into our freely available software package *BCBtoolkit*. This package can be employed to measure the pathophysiological mechanisms that cause cognitive deficits, and assess the relationship between these mechanisms and their consequential effects. Here we evaluated the risk of disconnection of classically defined white matter tracts and tested their relationship with category fluency performance. We then employed a tractography-based approach in order to reveal regions that were structurally disconnected by the lesion and assess their relationship with category fluency performance as compared to controls and other patients. Functional connectivity from the disconnected regions revealed large networks of interconnected areas. Within these regions/networks, measures of cortical thickness and of entropy of the rs-*f*MRI images were correlated to fluency performance, suggesting that some structural changes that occurred within these networks were due to the remote effect of a lesion that led to cognitive impairments. Consequently, the *BCBtoolkit* provided investigators with an ability to quantify the effect of brain damage upon the whole-brain, and explore its relationship to behavioural and cognitive abilities.

The investigation into the contribution of white matter tract disconnection is more than a century old approach and postulates an interruption in the course of white matter tracts in single case patients [83, 84]. Our method provides an anatomical rationale, as well as puts forth a statistical methodology enabling it to be extended to group-level studies. In the case of category fluency performance, this analysis particularly revealed a significant involvement of the anterior and long segments of the arcuate fasciculus, which are implicated in the language network [84-86]. However, these tracts have been defined by their shape for convenience (e.g. uncinate for hook-shaped connections or arcuate for arched-shaped connections) and should not be considered as a single unit, as ultimately, sub-portions could contribute differently to the elaboration of the cognition and behaviour.

Data driven maps of disconnection or ‘disconnectomes’ were consequently produced in order to identify the sub-portion of disconnected tracts and reveal the pattern of cortico-subcortical areas that were disconnected by the lesion. For the first time, we exemplify that this method can go beyond assessing only lesions, and can be employed to assess the relationship between disconnected areas and the patient’s neuropsychological performance. Here, this approach revealed that category fluency performance significantly decreased when several cortical and subcortical clusters were directly disconnected. The observed areas are consistent with previous lesion studies on fluency tasks [87]. Furthermore, each area identified as significantly involved in this analysis corresponded, almost systematically, to activation loci derived from *f*MRI studies in healthy controls performing fluency and/or categorisation tasks. This result suggests that the method appropriately identified altered functional networks contributing to the category fluency test. Nonetheless, one might argue that a cascade of polysynaptic events can influence behaviour and that dysfunctional, disconnected areas will also impact other indirectly connected areas.

In order to explore this additional dimension, we calculated the functional connectivity of the previously identified disconnected regions (i.e. clusters). In the case of the present analysis on category fluency performance, we revealed that the disconnected areas belonged to 3 large functional networks (i.e. facto-networks): a left dominant ventral fronto-parietal network, a mirror of the right-lateralized ventral attention network [88], which link key language territories [82] and is associated with executive functions [89, 90]. We additionally showed the involvement of the cingulo-opercular network, a network that interacts with the fronto-parietal control network for the control of goal-directed behaviours [91], which together with cortico-striatal network may also be linked to a reduced performance in fluency tasks [92]. The cingulo-opercular and cortico-striatal networks may also have contributed to performance through the global inertia or the ability of participants to allocate and coordinate resources during the task [93]. Finally, disconnection was associated with a significant reduction of functional connectivity in 2 out of the 3 factor-networks investigated. This is an important result, as functional connectivity appeared to be less significantly impaired in bilateral networks, suggesting that the proportion of the preserved functional network in both of the intact hemispheres may contribute to the strength of functional connectivity.

Changes in connectivity should induce changes in the microstructure of the areas of projection, and provoke cognitive or behavioural consequences. Measures of the cortical thickness revealed a significant thinning for some, but not all, directly disconnected areas. This result may reflect a potential transneuronal degeneration mechanism [41]. However, current limitations in spatial resolution and magnetic resonance imaging signal might have biased this measure in some regions due to changes in myelination in the lower layers of the cortex [94]. Cortical thickness analyses revealed that the left dominant ventral fronto-parietal network, whether it is seeded from MFg, IPs or subcortical structures in the left hemisphere, had a reduced cortical thickness associated to the category fluency performance. This result indicates a strong and encouraging relationship between the integrity of a network derived from measures of cortical thickness and behavioural performances. Future research can benefit from this approach to stratify patients' population and predict potential recovery.
Additionally, we explored whether structural changes such as other neural (e.g. synaptic plasticity) or non-neural factors (e.g. altered properties of the vasculature) could also be captured by measures of rs-*f*MRI entropy. Our results replicated recently published results, showing a strong decrease of entropy in both hemispheres when patients were compared to controls [95]. This indicates a large-scale effect of brain lesion on the overall blood oxygen level dependent dynamic of the brain. Finally, the result between patients (connected and disconnected patients) did not survive the correction for multiple comparisons, suggesting that, although promising, Shannon entropy measures of BOLD may be too noisy of a measure to capture very fine microstructural events with high enough statistical power.

Previous reports indicated that *AnaCOM* suffers from lower specificity than VLSM (Rorden et al., 2009). *AnaCOM* compares patients with controls performances, an approach that has previously been criticised [96]. In the context of our study, classical VLSM did not reveal any significant area involved with category fluency. In classical VLSM approaches, non-overlapping lesions are competing for statistical significance, fundamentally assuming that a single region is responsible for the symptoms. In the present study, we follow Associationist principles [97, 98] assuming that several interconnected regions will contribute to the elaboration of the behaviour. By comparing the performance between patients and a control population using *AnaCOM2*, several non-overlapping regions can reach significance, without competing for it. Hence, our results differ theoretically and methodologically from previous

approaches. Perhaps more importantly, the network of disconnected areas revealed by *AnaCOM2* is typically considered as functionally engaged for fluency and for categorization in healthy controls.

Newer multivariate methods have also been shown to provide superior performance compared to traditional VLSM [i.e. support vector regression lesion-symptom mapping, 6, 99]. For instance, such approaches have been employed to model the statistical relationship between damaged voxels in order to reduce false positives. In the *disconnectome maps*, this relationship has been pre-established using an anatomical prior derived from tractography in healthy controls. Therefore, it is not recommended to use multivariate approaches with the *disconnectome maps*, as they might come into conflict with the prebuilt anatomical association between the voxels. Additionally, these approaches require a much larger database of patients than the current study. Future research using large lesion databases will be required to explore the effect of multivariate statistical analysis on *disconnectome maps*.

Multivariate approaches also elegantly demonstrated that false positives can be driven by the vascular architecture [6]. This is an important limitation concerning any voxel and vascular lesion symptom mapping. Here, the group of patients explored included stroke and surgical lesions. Although we cannot exclude the participation of the vascular architecture in the present findings, the heterogeneity of the lesion included in our analyse may have limited this factor. Additionally, the statistical interaction between vascular architecture and the *disconnectome map* results remain to be explored in large database of lesions.

Methods used to estimate cortical thickness has previously been reported to perform poorly in peri-infarct regions, and the quality of the tissue segmentation may be particularly poor for stroke patients [73]. Here, we followed previously published recommendations for applying DiReCT [71] to the data from stroke patients: the lesion was masked out, the tissue segmentations were visually inspected, and manual boundary correction was performed when necessary (see **supplementary figure 2** for an example).

Finally, we applied our methods to the neural basis of category fluency as a proof of concept. The anatomy of category fluency should be, ideally, replicated in a larger sample of patients

including lesions involving the entire brain to provide a more comprehensive understanding of category fluency deficit after a brain lesion. While gathering such a large dataset of patients with brain lesions would have been impossible to achieve before, it might soon become possible thanks to collaborative initiatives such as the Enigma Consortium stroke recovery initiative (http://enigma.ini.usc.edu/ongoing/enigma-stroke-recovery/) [100].

Conclusion

Overall, using *BCBtoolkit*, researchers and clinicians can measure distant effects of brain lesions and associate these effects with neuropsychological outcomes. However, our methods require the manual delineation of lesion masks, automatization remaining a big challenge, especially on T1-images [100]. Taken together, these neuroimaging measures help discern the natural history of events occurring in the brain after a lesion, as well as assist in the localization of functions. These methods, gathered in the

BCBtoolkit, are freely available as **supplementary software** at http://toolkit.bcblab.com

Availability of supporting source code and requirements

- Project name: e.g. BCBtoolkit
- Project home page: http://toolkit.bcblab.com
- Operating system(s): Linux, MacOS
- Programming language: Java, Bash, R
- Other requirements: FSL, R, Python 2.7, Numpy
- License: BSD 3-Clause

Authors contribution

C.F. implemented the methods inside the *BCBtoolkit*, performed the analyses and wrote the manuscript. L.C. created the pipeline for the preprocessing of the resting state and for the functional correlation and revised the manuscript. S.K. conceived and help to upgrade the statistical analyses. C.R. collected the neuroimaging data. M.U. and E.V recruited the subjects, collected and built the database of patients and matched healthy controls including the neuropsychological and neuroimaging data and revised the manuscript. E.V. also

participated in the conception of the lesion study, and also provided funding for the database acquisition. R.L. provided funding for the study and revised the manuscript. M.T.d.S. wrote the manuscript, provided funding, conceived and coordinated the study, reviewed and collected neuroimaging data.

Acknowledgments

We thank Lauren Sakuma, Roberto Toro, Jean Daunizeau, Emmanuel Mandonnet, Beatrice Garcin, Stephanie J. Forkel and the BCBlab and Brainhack for useful discussions. The authors also thank the participants of this study as well as Prof. Claude Adam, Dr. Carole Azuar, Dr Marie-Laure Bréchemier, Dr. Dorian Chauvet, Dr Frédéric Clarençon Dr. Vincent Degos, Prof. Sophie Dupont, Prof. Damien Galanaud, Dr Béatrice Garcin, Dr. Florence Laigle, Dr Marc-Antoine Labeyrie, Dr. Anne Leger, Prof. Vincent Navarro, Prof. Pascale Pradat-Diehl, and Prof. Michel Wager for their help in recruiting the patients. The research leading to these results received funding from the "Agence Nationale de la Recherche" [grants number ANR-09-RPDOC-004-01 and number ANR-13- JSV4-0001-01] and from the Fondation pour la Recherche Médicale (FRM). Additional financial support comes from the program "Investissements d'avenir" ANR-10-IAIHU-06.

References

1. Jones, D.K., et al., *Non-invasive assessment of axonal fiber connectivity in the human brain via diffusion tensor MRI.* Magnetic Resonance in Medecine, 1999. **42**(1): p. 37-41.
2. Logothetis, N., *What we can do and what we cannot do with fMRI.* Nature, 2008. **453**(7197): p. 869-78.
3. Broca, P., *Perte de la parole, ramollissement chronique et destruction partielle du lobe antérieur gauche du cerveau.* Bull Soc Anthropol, 1861. **2**: p. 235-238, 301-321.
4. Damasio, H. and A. Damasio, *Lesion analysis in Neuropsychology*, ed. O.U. Press. 1989, New York.
5. Rorden, C., H.O. Karnath, and L. Bonilha, *Improving lesion-symptom mapping.* J Cogn Neurosci, 2007. **19**(7): p. 1081-8.
6. Mah, Y.H., et al., *Human brain lesion-deficit inference remapped.* Brain, 2014. **137**(Pt 9): p. 2522-31.
7. Bates, E., et al., *Voxel-based lesion-symptom mapping.* Nature Neuroscience, 2003. **6**(5): p. 448-50.
8. Dronkers, N.F., et al., *Lesion analysis of the brain areas involved in language comprehension.* Cognition, 2004. **92**(1-2): p. 145-77.

9. Karnath, H.O., S. Ferber, and M. Himmelbach, *Spatial awareness is a function of the temporal not the posterior parietal lobe.* Nature, 2001. **411**(6840): p. 950-3.
10. Bird, C.M., et al., *Visual neglect after right posterior cerebral artery infarction.* J Neurol Neurosurg Psychiatry, 2006. **77**(9): p. 1008-12.
11. Husain, M. and C. Kennard, *Visual neglect associated with frontal lobe infarction.* J Neurol, 1996. **243**(9): p. 652-7.
12. Mort, D.J., et al., *The anatomy of visual neglect.* Brain, 2003. **126**(Pt 9): p. 1986-97.
13. Coulthard, E.J., P. Nachev, and M. Husain, *Control over conflict during movement preparation: role of posterior parietal cortex.* Neuron, 2008. **58**(1): p. 144-57.
14. Volle, E., et al., *The functional architecture of the left posterior and lateral prefrontal cortex in humans.* Cereb Cortex, 2008. **18**(10): p. 2460-9.
15. Volle, E., R. Levy, and P.W. Burgess, *A new era for lesion-behavior mapping of prefrontal functions*, in *Principles of Frontal Lobe Function*, S. D.T. and R.T. Knight, Editors. 2013. p. 500–523.
16. Badre, D., et al., *Hierarchical cognitive control deficits following damage to the human frontal lobe.* Nat Neurosci, 2009. **12**(4): p. 515-22.
17. Geschwind, N., *Disconnexion syndromes in animals and man - Part I.* Brain, 1965. **88**: p. 237-294.
18. Geschwind, N., *Disconnexion syndromes in animals and man - Part II.* Brain, 1965. **88**: p. 585-644.
19. Carrera, E. and G. Tononi, *Diaschisis: past, present, future.* Brain, 2014. **137**(Pt 9): p. 2408-2422.
20. Finger, S., P.J. Koehler, and C. Jagella, *The Monakow concept of diaschisis: origins and perspectives.* Arch Neurol, 2004. **61**(2): p. 283-8.
21. Corbetta, M., et al., *Neural basis and recovery of spatial attention deficits in spatial neglect.* Nature Neuroscience, 2005. **8**(11): p. 1603-1610.
22. Thiebaut de Schotten, M., et al., *Damage to white matter pathways in subacute and chronic spatial neglect: a group study and 2 single-case studies with complete virtual "in vivo" tractography dissection.* Cereb Cortex, 2014. **24**(3): p. 691-706.
23. Thiebaut de Schotten, M., et al., *Visualization of disconnection syndromes in humans.* Cortex, 2008. **44**(8): p. 1097-103.
24. Urbanski, M., et al., *Reasoning by analogy requires the left frontal pole: lesion-deficit mapping and clinical implications.* Brain, 2016. **139**(Pt 6): p. 1783-1799.
25. Cazzoli, D., et al., *The influence of naturalistic, directionally non-specific motion on the spatial deployment of visual attention in right-hemispheric stroke.* Neuropsychologia, 2016. **92**: p. 181-189.
26. Piai, V., et al., *Neuroplasticity of language in lefthemisphere stroke: evidence linking subsecond electrophysiology and structural connections.* Human Brain Mapping, 2017.
27. Rudrauf, D., S. Mehta, and T.J. Grabowski, *Disconnection's renaissance takes shape: Formal incorporation in group-level lesion studies.* Cortex, 2008. **44**(8): p. 1084-96.
28. Fridriksson, J., et al., *Damage to the anterior arcuate fasciculus predicts non-fluent speech production in aphasia.* Brain, 2013. **136**(Pt 11): p. 3451-60.
29. Hope, T.M., et al., *Distinguishing the effect of lesion load from tract disconnection in the arcuate and uncinate fasciculi.* Neuroimage, 2016. **125**: p. 1169-73.
30. Corbetta, M., et al., *Common behavioral clusters and subcortical anatomy in stroke.* Neuron, 2015. **85**(5): p. 927-41.

31. Griffis, J.C., et al., *Damage to white matter bottlenecks contributes to language impairments after left hemispheric stroke.* Neuroimage Clin, 2017. **14**: p. 552-565.
32. He, B.J., et al., *Breakdown of functional connectivity in frontoparietal networks underlies behavioral deficits in spatial neglect.* Neuron, 2007. **53**(6): p. 905-18.
33. Turken, A.U. and N.F. Dronkers, *The neural architecture of the language comprehension network: converging evidence from lesion and connectivity analyses.* Front Syst Neurosci, 2011. **5**: p. 1.
34. Bonilha, L., et al., *The brain connectome as a personalized biomarker of seizure outcomes after temporal lobectomy.* Neurology, 2015. **84**(18): p. 1846-53.
35. Thiebaut de Schotten, M., et al., *From Phineas Gage and Monsieur Leborgne to H.M.: Revisiting Disconnection Syndromes.* Cereb Cortex, 2015. **25**(12): p. 4812-27.
36. Kuceyeski, A., et al., *Structural connectome disruption at baseline predicts 6-months post-stroke outcome.* Hum Brain Mapp, 2016. **37**(7): p. 2587-601.
37. Yourganov, G., et al., *Multivariate Connectome-Based Symptom Mapping in Post-Stroke Patients: Networks Supporting Language and Speech.* J Neurosci, 2016. **36**(25): p. 6668-79.
38. Kuceyeski, A., et al., *The Network Modification (NeMo) Tool: elucidating the effect of white matter integrity changes on cortical and subcortical structural connectivity.* Brain Connect, 2013. **3**(5): p. 451-63.
39. Boes, A.D., et al., *Network localization of neurological symptoms from focal brain lesions.* Brain, 2015. **138**(Pt 10): p. 3061-75.
40. Munafo, M., *Metascience: Reproducibility blues.* Nature, 2017. **543**(7647): p. 619-620.
41. Fornito, A., A. Zalesky, and M. Breakspear, *The connectomics of brain disorders.* Nat Rev Neurosci, 2015. **16**(3): p. 159-72.
42. Feeney, D.M. and J.C. Baron, *Diaschisis.* Stroke, 1986. **17**(5): p. 817-30.
43. Cowan, W., *Contemporary Research Methods in Neuroanatomy*. 1970: Springer.
44. Bredesen, D.E., *Neural apoptosis.* Annals of Neurology, 1995. **38**(6): p. 839-51.
45. Capurso, S.A., et al., *Deafferentation causes apoptosis in cortical sensory neurons in the adult rat.* J Neurosci, 1997. **17**(19): p. 7372-84.
46. Schaechter, J.D., et al., *Structural and functional plasticity in the somatosensory cortex of chronic stroke patients.* Brain, 2006. **129**(Pt 10): p. 2722-33.
47. Xing, S., et al., *Right hemisphere grey matter structure and language outcomes in chronic left hemisphere stroke.* Brain, 2016. **139**(Pt 1): p. 227-41.
48. Gladsjo, J.A., et al., *Norms for letter and category fluency: demographic corrections for age, education, and ethnicity.* Assessment, 1999. **6**(2): p. 147-78.
49. MacPherson, S.E. and S. Della Sala, *Handbook of Frontal Lobe Assessment*. 2015, Oxford: Oxford University Press.
50. Lezak, M., *Neuropsychological assessment*. 1995, Oxford: Oxford University Press.
51. Jones, D.K., et al., *Spatial normalization and averaging of diffusion tensor MRI data sets.* NeuroImage, 2002. **17**(2): p. 592-617.
52. Nachev, P., et al., *Enantiomorphic normalization of focally lesioned brains.* Neuroimage, 2008. **39**(3): p. 1215-26.
53. Zhang, Y., M. Brady, and S.M. Smith, *Segmentation of brain MR images through a hidden Markov random field model and the expectation-maximization algorithm.* IEEE Trans. Med. Imaging, 2001. **20**: p. 45–57.
54. Smith, S.M., *Fast robust automated brain extraction.* Hum Brain Mapp, 2002. **17**(3): p. 143-55.

55. Avants, B.B., et al., *A reproducible evaluation of ANTs similarity metric performance in brain image registration.* Neuroimage, 2011. **54**(3): p. 2033-44.
56. Rojkova, K., et al., *Atlasing the frontal lobe connections and their variability due to age and education: a spherical deconvolution tractography study.* Brain Struct Funct, 2016. **221**(3): p. 1751-66.
57. Catani, M., et al., *Short frontal lobe connections of the human brain.* Cortex, 2012. **48**(2): p. 273-91.
58. Von Der Heide, R.J., et al., *Dissecting the uncinate fasciculus: disorders, controversies and a hypothesis.* Brain, 2013. **136**(Pt 6): p. 1692-707.
59. Duffau, H., et al., *New insights into the anatomo-functional connectivity of the semantic system: a study using cortico-subcortical electrostimulations.* Brain, 2005. **128**(Pt 4): p. 797-810.
60. Catani, M., D.K. Jones, and D.H. ffytche, *Perisylvian language networks of the human brain.* Annals of Neurolology, 2005. **57**(1): p. 8-16.
61. Catani, M. and V. Bambini, *A model for Social Communication And Language Evolution and Development (SCALED).* Curr Opin Neurobiol, 2014. **28**: p. 165-71.
62. Thiebaut de Schotten, M., et al., *A lateralized brain network for visuospatial attention.* Nat Neurosci, 2011. **14**(10): p. 1245-6.
63. Ciccarelli, O., et al., *Diffusion tractography based group mapping of major white-matter pathways in the human brain.* NeuroImage, 2003. **19**(4): p. 1545-55.
64. Thiebaut de Schotten, M., et al., *Atlasing location, asymmetry and inter-subject variability of white matter tracts in the human brain with MR diffusion tractography.* Neuroimage, 2011. **54**(1): p. 49-59.
65. Kinkingnehun, S., et al., *A novel approach to clinical-radiological correlations: Anatomo-Clinical Overlapping Maps (AnaCOM): method and validation.* NeuroImage, 2007. **37**(4): p. 1237-49.
66. Yarkoni, T., et al., *Large-scale automated synthesis of human functional neuroimaging data.* Nat Methods, 2011. **8**(8): p. 665-70.
67. Jenkinson, M., et al., *Improved optimization for the robust and accurate linear registration and motion correction of brain images.* Neuroimage, 2002. **17**(2): p. 825-41.
68. Woolrich, M.W., et al., *Bayesian analysis of neuroimaging data in FSL.* Neuroimage, 2009. **45**(1 Suppl): p. S173-86.
69. Kenney, J., *Mathematics of Statistics*. 1939, London: Chapman & Hall.
70. Tononi, G., G.M. Edelman, and O. Sporns, *Complexity and coherency: integrating information in the brain.* Trends Cogn Sci, 1998. **2**(12): p. 474-84.
71. Das, S.R., et al., *Registration based cortical thickness measurement.* Neuroimage, 2009. **45**(3): p. 867-79.
72. Tustison, N.J., et al., *Large-scale evaluation of ANTs and FreeSurfer cortical thickness measurements.* Neuroimage, 2014. **99**: p. 166-79.
73. Li, Q., et al., *Cortical thickness estimation in longitudinal stroke studies: A comparison of 3 measurement methods.* Neuroimage Clin, 2015. **8**: p. 526-35.
74. Shannon, C.E., *The mathematical theory of communication. 1963.* MD Comput, 1997. **14**(4): p. 306-17.
75. Gray, R., *Entropy and Information Theory*. 2011: Springer US.
76. Ogawa, S., et al., *Brain magnetic resonance imaging with contrast dependent on blood oxygenation.* Proc Natl Acad Sci U S A, 1990. **87**(24): p. 9868-72.

77. Biswal, B., et al., *Functional connectivity in the motor cortex of resting human brain using echo-planar MRI.* Magn Reson Med, 1995. **34**(4): p. 537-41.
78. Hebb, D.O., *The Organization of Behavior: A Neuropsychological Theory.* 1949, New York: Wiley and Sons.
79. de Haan, B. and H.O. Karnath, *'Whose atlas I use, his song I sing?' - The impact of anatomical atlases on fiber tract contributions to cognitive deficits after stroke.* Neuroimage, 2017. **163**: p. 301-309.
80. Sadaghiani, S. and M. D'Esposito, *Functional Characterization of the Cingulo-Opercular Network in the Maintenance of Tonic Alertness.* Cereb Cortex, 2015. **25**(9): p. 2763-73.
81. Voorn, P., et al., *Putting a spin on the dorsal-ventral divide of the striatum.* Trends Neurosci, 2004. **27**(8): p. 468-74.
82. Smith, S.M., et al., *Correspondence of the brain's functional architecture during activation and rest.* Proc Natl Acad Sci U S A, 2009. **106**(31): p. 13040-5.
83. Lichtheim, L., *On aphasia.* Brain, 1885. **7**: p. 433-484.
84. Catani, M. and D.H. ffytche, *The rises and falls of disconnection syndromes.* Brain, 2005. **128**(Pt 10): p. 2224-39.
85. Dronkers, N.F., et al., *Paul Broca's historic cases: high resolution MR imaging of the brains of Leborgne and Lelong.* Brain, 2007. **130**(Pt 5): p. 1432-41.
86. Forkel, S.J., et al., *Anatomical predictors of aphasia recovery: a tractography study of bilateral perisylvian language networks.* Brain, 2014. **137**(Pt 7): p. 2027-39.
87. MacPherson, S.E., et al., *Handbook of Frontal Lobe Assessment*. 2015, Oxford: Oxford University Press.
88. Fox, M.D., et al., *Spontaneous neuronal activity distinguishes human dorsal and ventral attention systems.* Proc Natl Acad Sci USA, 2006. **103**(26): p. 10046-51.
89. Parlatini, V., et al., *Functional segregation and integration within fronto-parietal networks.* Neuroimage, 2017. **146**: p. 367-375.
90. Power, J.D. and S.E. Petersen, *Control-related systems in the human brain.* Curr Opin Neurobiol, 2013. **23**(2): p. 223-8.
91. Gratton, C., et al., *Distinct Stages of Moment-to-Moment Processing in the Cinguloopercular and Frontoparietal Networks.* Cereb Cortex, 2016.
92. Chouiter, L., et al., *Partly segregated cortico-subcortical pathways support phonologic and semantic verbal fluency: A lesion study.* Neuroscience, 2016. **329**: p. 275-83.
93. Bonnelle, V., et al., *Salience network integrity predicts default mode network function after traumatic brain injury.* Proc Natl Acad Sci U S A, 2012. **109**(12): p. 4690-5.
94. Wagstyl, K., et al., *Cortical thickness gradients in structural hierarchies.* Neuroimage, 2015. **111**: p. 241-50.
95. Saenger, V.M., et al., *Linking Entropy at Rest with the Underlying Structural Connectivity in the Healthy and Lesioned Brain.* Cerebral Cortex, 2017. **(in press)**(2017).
96. Rorden, C., J. Fridriksson, and H.O. Karnath, *An evaluation of traditional and novel tools for lesion behavior mapping.* Neuroimage, 2009. **44**(4): p. 1355-62.
97. Geschwind, N., *Disconnexion syndromes in animals and man. II.* Brain, 1965. **88**(3): p. 585-644.
98. Geschwind, N., *Disconnexion syndromes in animals and man. I.* Brain, 1965. **88**(2): p. 237-94.

99. Zhang, Y., et al., *Multivariate lesion-symptom mapping using support vector regression.* Hum Brain Mapp, 2014. **35**(12): p. 5861-76.

100. Liew, S.-L., et al., *The Anatomical Tracings of Lesions After Stroke (ATLAS) Dataset - Release 1.1.* bioRxiv, 2017.

Captions

Fig. 1: Category fluency performance (mean performance with 95% confidence intervals) for patients with (dark grey) or without (light grey) disconnection of each tract of interest. The green intervals indicate the range of controls' performance corresponding to 95% confidence intervals. * $p < 0.05$

Fig. 2: Areas directly disconnected by the lesion that significantly contributed to a decreased score on category fluency task (referred to as "disconnected areas" in the manuscript). a) Representative slices from *disconnectome maps* computed for category fluency performance, blue clusters indicate group average low performance and red high performance. b) Brain areas contributing significantly after correction for multiple comparisons. c) Category fluency performance (mean performance with 95% confidence intervals) for patients with (dark grey) or without (light grey) disconnection of each of the examined cortical regions. The green interval indicates performance in matched controls with 95% confidence intervals. preSMA: presupplementary motor area, IPs: intraparietal sulcus, MFg: middle frontal gyrus, pars Op.: frontal pars opercularis, A: anterior group of thalamic nuclei, VA ventral anterior VLp: ventrolateral posterior, VLa: ventrolateral anterior. * $p < 0.05$ Bonferroni-Holm corrected for multiple comparisons.

Fig. 3: Areas classically activated with *f*MRI ($p< 0.01$ FDR corrected) during fluency (pink) and categorization (cyan) tasks. Areas involved in both fluency and categorization are highlighted in dark blue.

Fig. 4: Functional networks involving the identified disconnected areas, as defined by resting state functional connectivity. Top panel, main cortical networks involving the disconnected areas revealed by a principal component analysis. Bottom left panel, principal component

analysis of the raw functional connectivity result. Bottom right panel, strength of the functional connectivity for patients with (dark grey) or without (light grey) involvement of the functional network. CO: Cingulo-opercular network, CS: cortico-striatal network, VFP: Ventral fronto-parietal network. * indicates $p < 0.05$; **, $p < 0.01$

Fig. 5: Dimensional relationship between cortical thickness measured in rs-*f*MRI disconnected networks and category fluency. Note that regression lines (in black) and intervals (mean confidence intervals in red) are for illustrative purposes since we performed a rank-order correlation.

 Click here to download Table table1.docx

Table 1: Demographical and clinical data

ID	Age (years)	Education (years)	Gender	Lesion side	Lesion volume (mm^3)	Lesion delay (months)	Aetiology
P01	56	17	F	right	255	7	stroke
P02	55	19	M	left	34374	76	hematoma
P03	46	17	F	left	14847	126	stroke
P04	50	11	F	left	110145	137	surgery
P05	64	14	M	right	59048	119	stroke
P06	32	16	F	right	15946	129	epilepsy
P07	51	11	M	bilateral	113170	54	stroke
P08	70	5	F	left	51530	85	surgery
P09	47	11	M	right	7809	115	hematoma
P10	62	13	F	bilateral	21295	14	hematoma
P11	41	16	M	right	55848	29	surgery
P12	46	12	M	bilateral	2542	51	hematoma
P13	67	15	M	left	4102	133	stroke
P14	49	9	M	bilateral	14929	19	hematoma
P15	36	14	F	right	40854	82	surgery
P16	40	22	F	left	24829	56	hematoma
P17	40	14	M	bilateral	14364	7	hematoma
P18	23	16	F	right	21681	47	surgery
P19	54	22	M	right	51897	48	stroke
P20	71	17	M	left	25779	91	hematoma
P21	23	15	F	right	29513	36	surgery
P22	27	9	F	left	12986	30	surgery
P23	26	13	F	left	2640	19	surgery
P24	32	14	F	left	12653	4	surgery
P25	59	16	F	left	97	9	hematoma
P26	26	13	F	left	26928	32	stroke
P27	58	12	M	left	1026	3	stroke
P29	75	12	F	left	14938	16	hematoma
P30	52	13	F	right	11978	20	surgery
P31	58	12	M	right	13263	21	surgery
P32	62	5	M	right	20281	9	surgery
P33	41	17	M	left	7463	29	surgery

P34	42	17	M	left	24319	6	Infection
P35	60	12	M	right	41897	24	surgery
P36	51	14	F	right	39213	17	surgery
P37	51	12	F	right	8133	48	surgery
P38	33	17	M	right	140947	48	surgery

Table 2: White matter tracts disconnection relationship with category fluency statistical report. Results are not corrected for multiple comparisons**.** n1, number of disconnected patients; n2, number of spared patients

	3 groups comparison		Patients disconnected and connected		Patients disconnected and controls		Patients connected and controls		n1	n2
Tracts	K	P value	U	P value	U	P value	U	P value		
Cingulum Left	19	0.0001	141	0.2035	189	0.0003	277	0.0003	16	21
Cingulum Right	19	0.0001	161	0.5	280	0.0001	187	0.0019	23	14
Uncinate Left	19	0.0001	148	0.3994	176	0.0027	291	0.0001	13	24
Uncinate Right	19	0.0001	167	0.4635	209	0.0004	258	0.0003	17	20
Arcuate Anterior Segment Left	22	0.0000	29	0.0116	12	0.0004	454	0.0001	5	32
Arcuate Anterior Segment Right	19	0.0001	126	0.3855	118	0.0025	348	0.0001	16	21
Arcuate Long Segment Left	23	0.0000	32	0.0059	13	0.0001	453	0.0002	6	31
Arcuate Long Segment Right	19	0.0001	107	0.2559	117	0.0068	349	0.0001	9	28
Inferior Fronto Occipital fasciculus Left	19	0.0001	165	0.5	196	0.0011	271	0.0001	15	22
Inferior Fronto Occipital fasciculus Right	19	0.0001	157	0.3457	199	0.0002	268	0.0004	17	20
Frontal Aslant Tract Left	21	0.0000	90	0.0389	90	0.0001	377	0.0004	11	26
Frontal Aslant tract Right	19	0.0001	155	0.3131	194	0.0001	272	0.0012	18	19
Frontal Inferior Longitudinal Left	21	0.0000	69	0.0216	54	0.0001	413	0.0004	9	28
Frontal Inferior Longitudinal Right	19	0.0001	140	0.3051	171	0.0022	295	0.0001	13	34
Frontal Superior Longitudinal Left	20	0.0000	75	0.0352	73	0.0004	393	0.0002	9	28
Frontal Superior Longitudinal Right	19	0.0001	129	0.1992	120	0.0001	346	0.0005	13	34

Table 3: Direct disconnection of brain areas relationship with category fluency statistical report. Unless specified, p values are Bonferroni-Holms corrected for multiple comparisons. n1, number of disconnected patients; n2, number of connected patients

		3 groups comparison		Patients disconnected and connected (uncorrected)		Patients disconnected and controls		Patients connected and controls		n1	n2
	Disconnected areas	Kruskal Wallis	P value	U	P value	U	P value	U	P value		
FLUENCY SCORE LEFT HEMISPHERE	PreSMA	21.34128456	0.0059	212	0.0456	88.5	0.0248	377.5	0.0329	12	25
	IPs	23.35102548	0.0023	172	0.0098	18	0.0295	448	0.0324	7	30
	Cingulate ant	18.6697471	0.0125	160.5	0.7452	304	0.0248	162	0.0329	25	12
	Cingulate mid	21.52289636	0.0054	219	0.0464	95.5	0.0141	370.5	0.0329	13	24
	MFg	23.12826675	0.0026	237	0.0103	81.5	0.0054	384.5	0.0329	13	24
	Opercularis, striatum, thalamus	23.99647373	0.0017	179	0.0043	16	0.0249	450	0.0324	7	30
FLUENCY SCORE RIGHT HEMISPHERE	PreSMA	22.92724537	0.0028	208	0.0130	50.5	0.0138	415.5	0.0329	10	27
	Cingulate ant	18.8698263	0.0125	185.5	0.6470	225	0.0330	241	0.0329	20	17
	Cingulate mid	18.62681983	0.0125	137.5	0.6966	317	0.0415	149	0.0329	25	12
	MFg	22.06856039	0.0042	196	0.0382	54	0.0180	412	0.0329	10	27
	Striatum	18.604408	0.0125	154.5	0.8966	310	0.0313	156	0.0329	14	23
	Thalamus	19.117101	0.0125	192	0.5326	202	0.0248	264	0.0329	14	23

Table 4: Cortical thickness and *f*MRI entropy measures in disconnected areas. Uncorrected P values. n1, number of disconnected patients; n2, number of spared patients

		3 groups comparison		Patients disconnected and connected		Patients disconnected and controls		Patients connected and controls		n1	n2
	Disconnected areas	Kruskal Wallis	P value	U	P value	U	P value	U	P value		
CORTICAL THICKNESS LEFT HEMISPHERE	PreSMA	8	0.0224	109	0.0944	168	0.0057	514	0.0565	12	25
	IPs	9	0.0131	54	0.0251	59	0.0019	667	0.1134	7	30
	Cingulate ant	5	0.0822	110	0.1000	465	0.0175	269	0.2061	25	12
	Cingulate mid	5	0.0759	139	0.2998	278	0.1436	435	0.0137	13	24
	MFg	8	0.0143	109	0.0695	172	0.0028	502	0.0710	13	24
	Opercularis	13	0.0012	40	0.0061	44	0.0006	583	0.0225	7	30
CORTICAL THICKNESS RIGHT HEMISPHERE	PreSMA	4	0.1328	134	0.4931	214	0.1711	523	0.0254	10	27
	Cingulate ant	7	0.0296	167	0.4696	362	0.0191	295	0.0169	20	17
	Cingulate mid	23	0.0000	61	0.0020	223	0.1414	254	0.1415	25	12
	MFg	6	0.0587	116	0.2634	163	0.0359	538	0.0359	10	27
SHANNON ENTROPY LEFT HEMISPHERE	PreSMA	24	0.0000	86	0.2171	85	0.0004	210	0.0000	12	25
	IPs	27	0.0000	40	0.0422	18	0.0002	260	0.0000	7	30
	Cingulate ant	44	0.0000	84	0.1158	109	0.0000	3	0.0000	25	12
	Cingulate mid	36	0.0000	97	0.3029	45	0.0000	127	0.0000	13	24
	MFg	16	0.0004	100	0.4246	125	0.0043	272	0.0003	13	24
	Opercularis, striatum, thalamus	17	0.0002	65	0.3680	75	0.0181	287	0.0001	7	30
SHANNON ENTROPY RIGHT HEMISPHERE	PreSMA	8	0.0177	82	0.2364	117	0.0078	413	0.0243	10	27
	Cingulate ant	55	0.0000	81	0.0640	16	0.0000	4	0.0000	20	17
	Cingulate mid	22	0.0000	111	0.4596	203	0.0001	114	0.0003	25	12
	MFg	22	0.0000	55	0.0497	136	0.0533	209	0.0000	10	27
	Striatum	23	0.0000	110	0.4436	202	0.0001	100	0.0001	14	23
	Thalamus	58	0.0000	67	0.0204	0	0.0000	6	0.0000	14	23

Figure1

Click here to download Figure Fig1.jpg

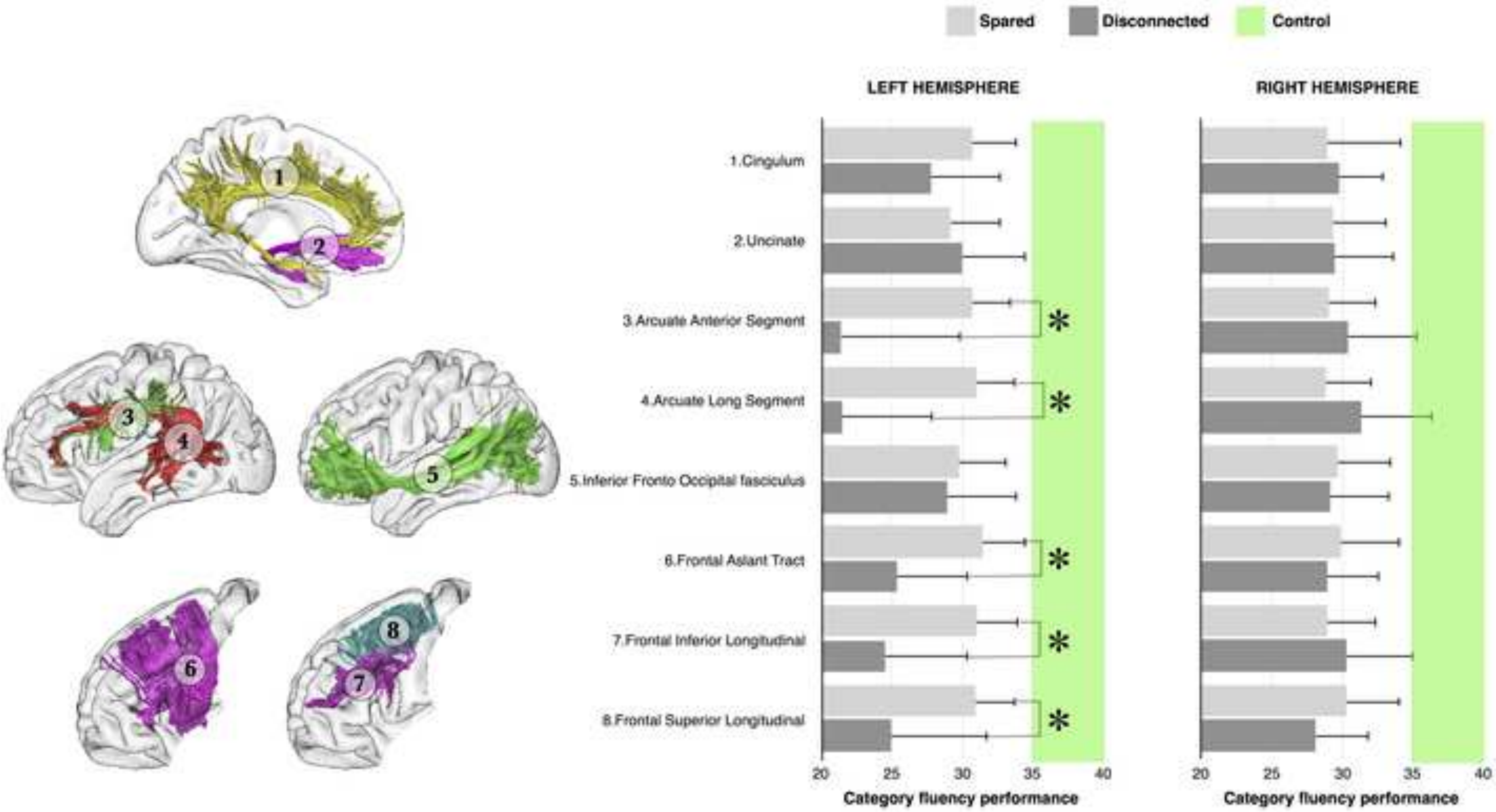

Figure2

Click here to download Figure Fig2.jpg

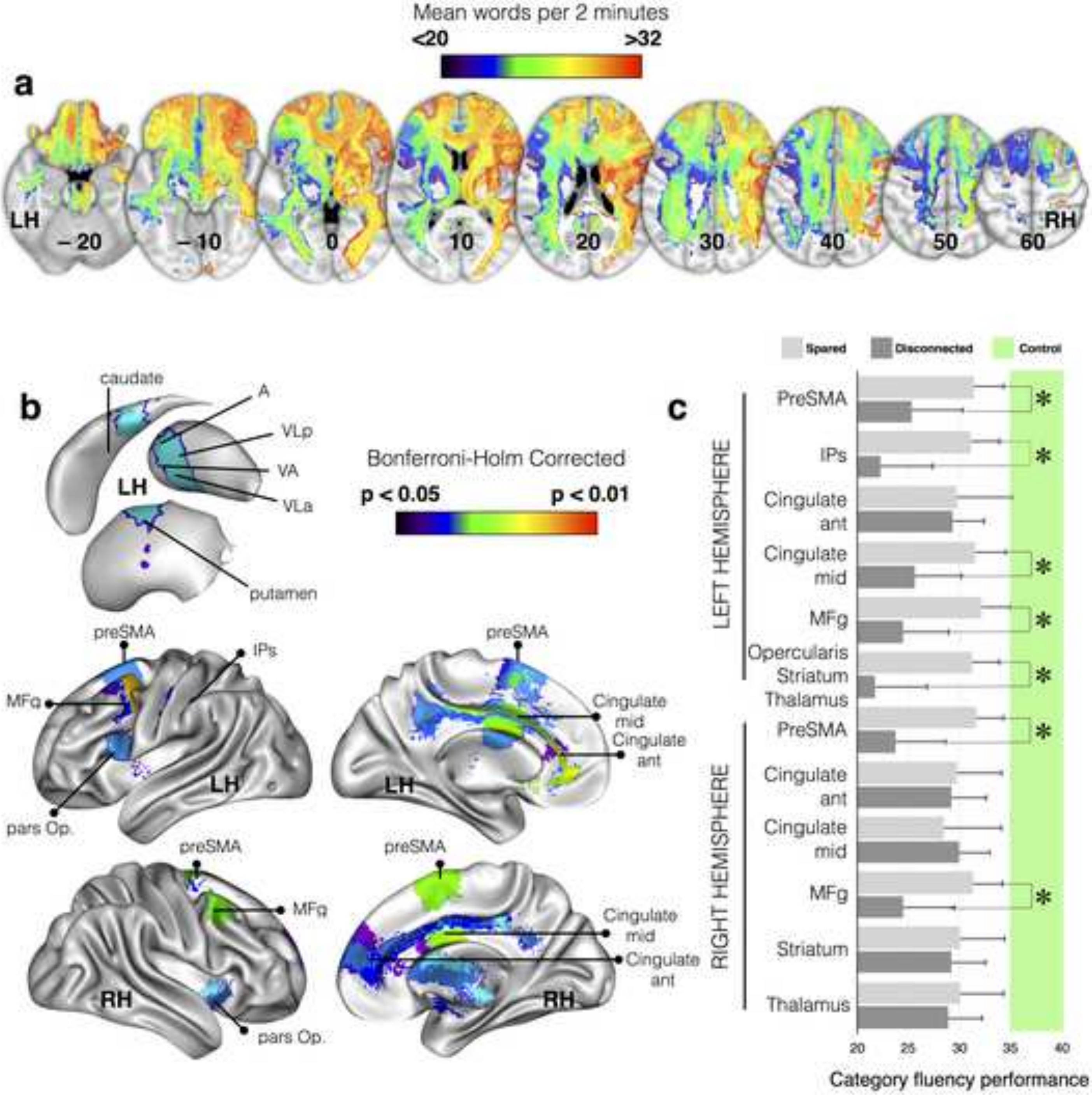

Figure3

Click here to download Figure Fig3.jpg

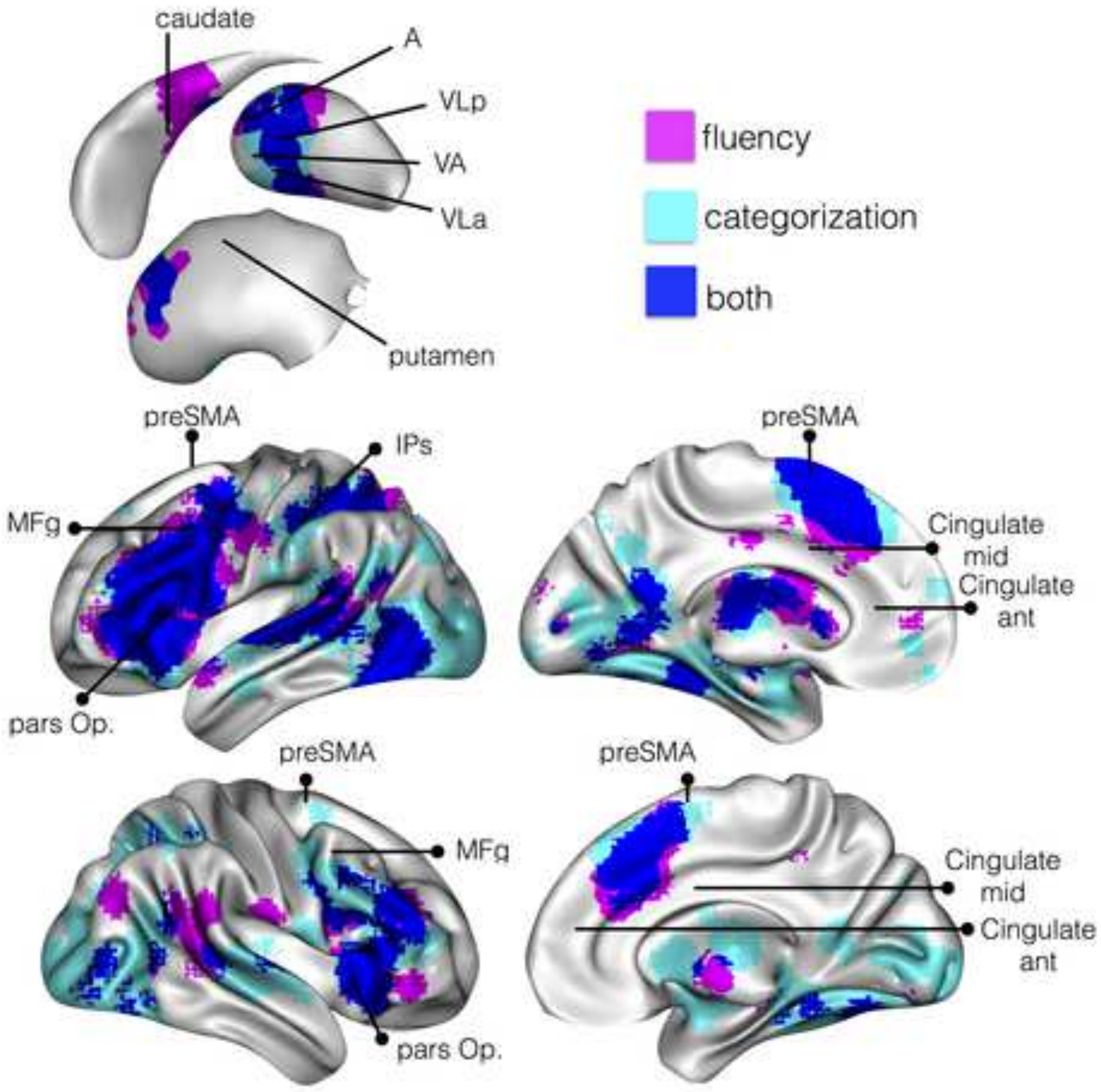

Figure4

Click here to download Figure Fig4.jpg

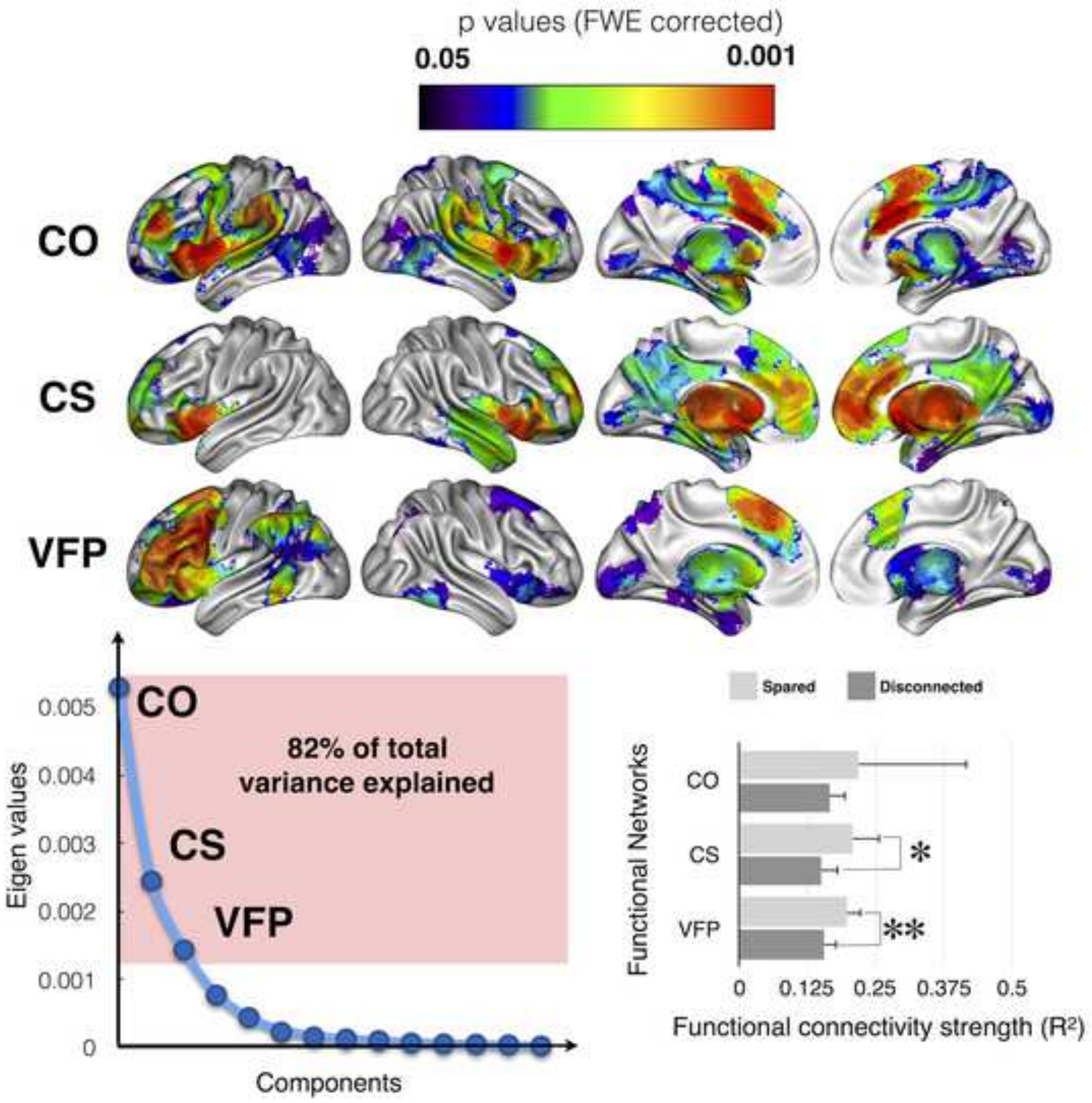

Figure5

Click here to download Figure Fig5.jpg

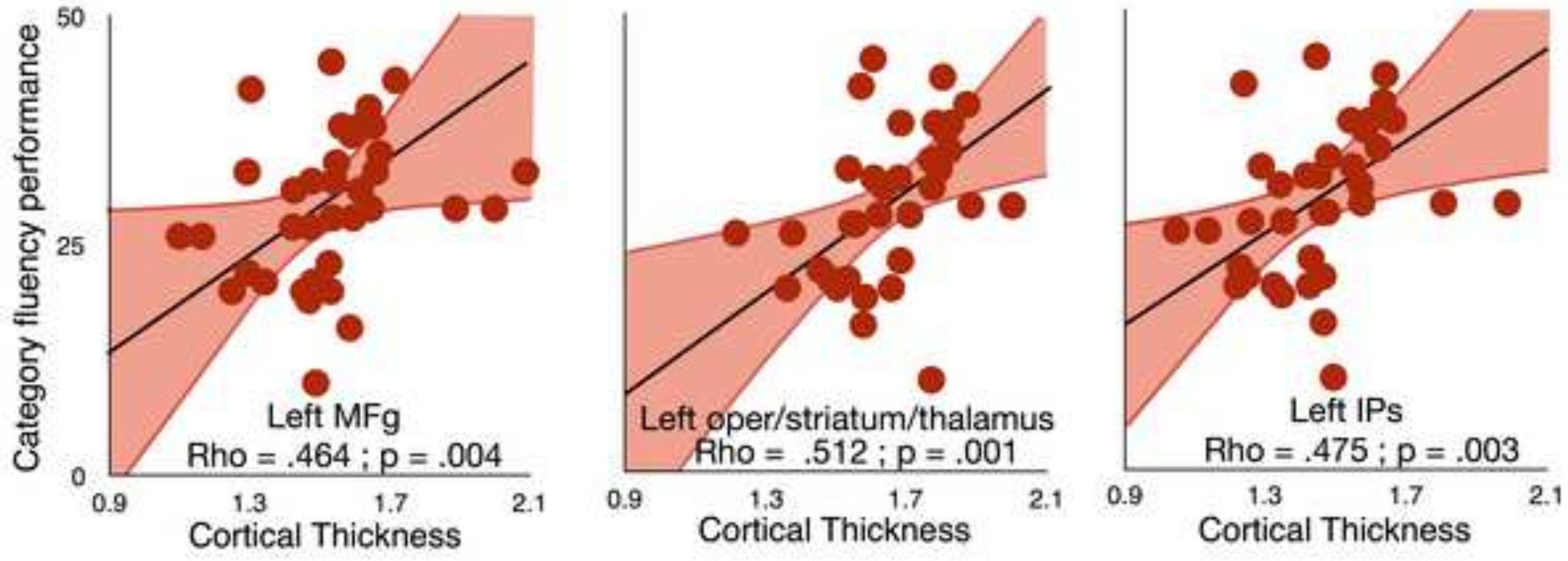

Click here to access/download
Supplementary Material
Supplementary_material.docx

Supplementary Figure 1

Click here to access/download
Supplementary Material
Supplementary figure 1.docx

Supplementary Figure 2

Click here to access/download
Supplementary Material
Supplementary figure 2.docx

Supplementary Figure 3

Click here to access/download
Supplementary Material
Supplementary figure 3.docx

Click here to access/download
Supplementary Material
Reviewers_response.docx

Dear dr. Nogoy,

Thank you for considering the revised version of our manuscript untitled '**Advanced lesion symptom mapping analyses and implementation as BCBtoolkit**' for a *technical report* in Gigascience.

We followed the reviewers' recommendations and clarified the methods. Our response to the reviewers includes figures and is uploaded as supplementary material with the manuscript.

Although we cannot share any sample of this dataset because we do not have the authorization, we made one subject available for the reviewers to assess the quality of the work. We hope this material will remain at their discretion.
https://www.dropbox.com/s/ikgszc8wbt5kej2/Data4review.zip?dl=0

We added a statement in the manuscript mentioning the inability to fully share the actual clinical sample data due to consent issues.

We also report additional analyses regressing out age and lesion size from the correlation analysis between category fluency and cortical thickness and are happy to report that the results remained significant.

We are looking forward to your assessment.

Sincerely,

Michel Thiebaut de Schotten
