# Supplementary material for: Advanced lesion symptom mapping analyses and implementation as BCBtoolkit
Source: Gigascience. 2018 Feb 8;7(3):giy004. doi: 10.1093/gigascience/giy004 (PMC5863218; doi:10.1093/gigascience/giy004)
Supplement: Supplement Materials [file giy004_supp.zip › Supplementary figure 2.docx]

**
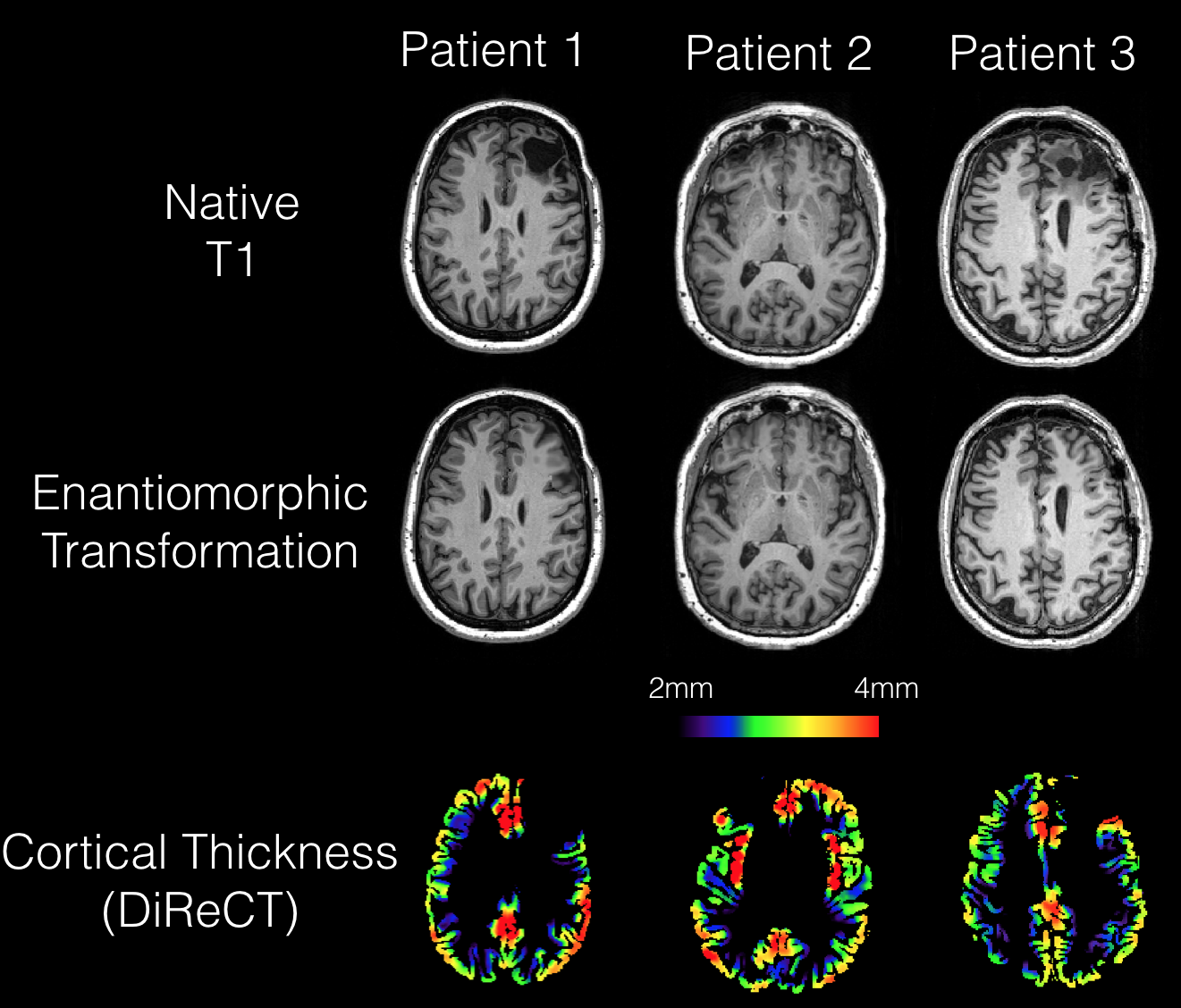
**

**Sup Fig. 2**: Native T1, enantiomorphic deformation and derived Cortical Thickness of three representative subjects
