# Supplementary material for: Advanced lesion symptom mapping analyses and implementation as BCBtoolkit
Source: Gigascience. 2018 Feb 8;7(3):giy004. doi: 10.1093/gigascience/giy004 (PMC5863218; doi:10.1093/gigascience/giy004)
Supplement: Supplement Materials [file giy004_supp.zip › Supplementary figure 3.docx]

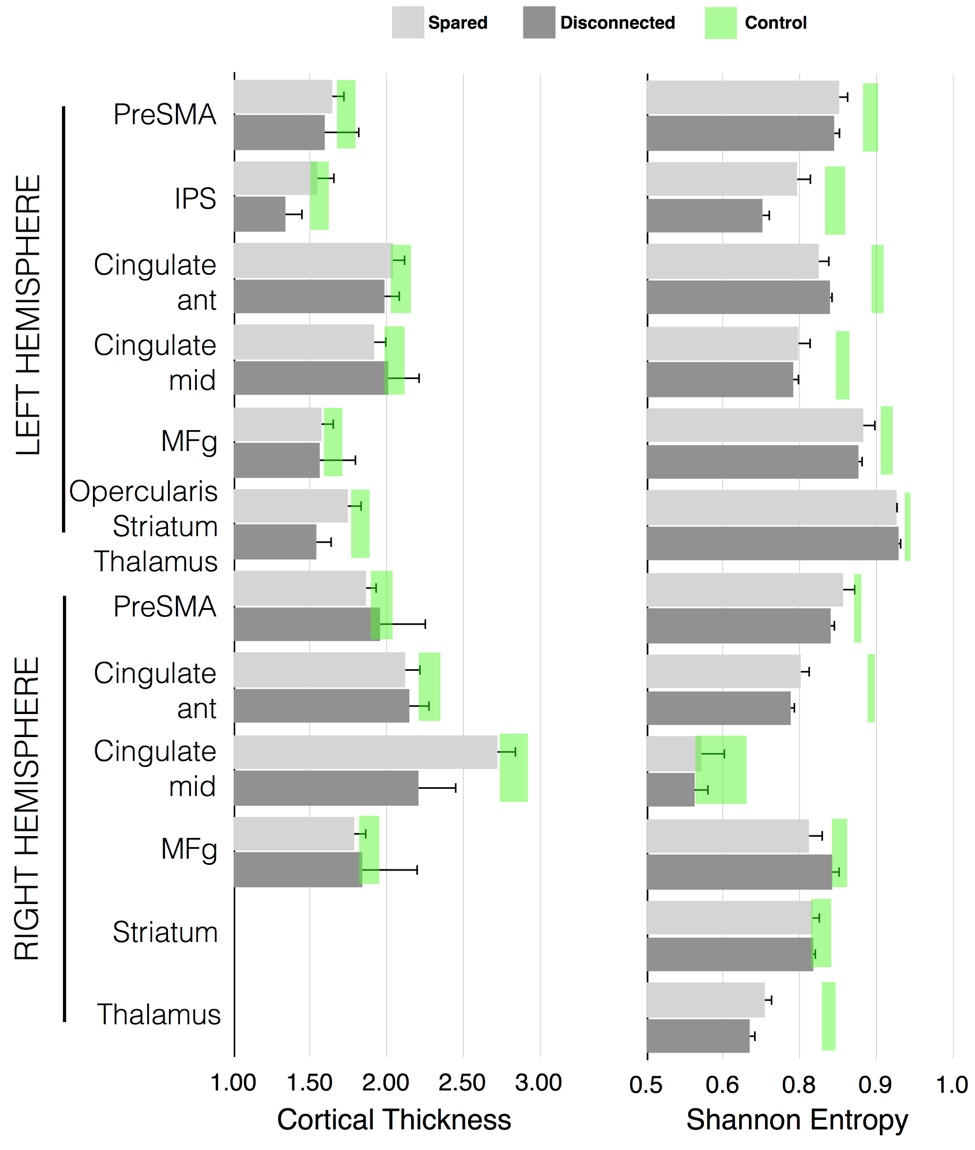


**Sup Fig. 3**: Cortical thickness and Shannon entropy measures (mean with 95% confidence intervals) in patients with (dark grey) or without (light grey) disconnection for each of the disconnected areas. The green interval indicates performance in matched controls with 95% confidence intervals.
