# Supplementary material for: Advanced lesion symptom mapping analyses and implementation as BCBtoolkit
Source: Gigascience. 2018 Feb 8;7(3):giy004. doi: 10.1093/gigascience/giy004 (PMC5863218; doi:10.1093/gigascience/giy004)
Supplement: Supplement Materials [file giy004_supp.zip › Supplementary_material.docx]

**Advanced lesion symptom mapping analyses and implementation as BCBtoolkit**

**Supplementary material**

Foulon C^a,b,c*^, Cerliani L^a,b,c^, Kinkingnéhun S^a^, Levy R^b^, Rosso C^c,d^, Urbanski M^a,b,e^, Volle E^a,b,c^, Thiebaut de Schotten M^a,b,c*^

^a^ Brain Connectivity and Behaviour Group, Brain and Spine Institute, Paris France.

^b^ Frontlab, Institut du Cerveau et de la Moelle épinière (ICM), UPMC UMRS 1127, Inserm U 1127, CNRS UMR 7225, Paris, France.

^c^ Centre de Neuroimagerie de Recherche CENIR, Groupe Hospitalier Pitié-Salpêtrière, Paris, France.

^d^ APHP, Urgences Cérébro-Vasculaires, Groupe Hospitalier Pitié-Salpêtrière, Paris, France.

^e^ Medicine and Rehabilitation Department, Hôpitaux de Saint-Maurice, Saint-Maurice, France.

- Corresponding authors [hd.chrisfoulon@gmail.com](mailto:hd.chrisfoulon@gmail.com) and [michel.thiebaut@gmail.com](mailto:michel.thiebaut@gmail.com)

**Competing interests:**

The authors declare that they have no competing interests

The optimal number of participants was calculated for *disconnectome maps* from separate paired populations of equal gender distribution. This approach was repeated for groups consisting of 4, 6, 8, 10, 12, 14, 16, 18 and 20 subjects. Squared spatial Pearson’s correlations between each pair (i.e. square of fslcc from FSL) were employed to calculate the percentage of shared variance (i.e. the similarity). This analysis indicates a steep increase of shared variance between disconnectome maps produced from 4 to 10 participants followed by a slower increase from 10 to 20 participants. This result indicates that, using the disconnectome, 10 subjects are sufficient to produce a good enough disconnectome map that matches the overall population (above 70% of shared variance). A larger dataset (n = 36) can be downloaded on our website (http://www.bcblab.com/opendata). Additionally, HCP 7T data (n = 166) have been prepared for the disconnectome and are available on demand to the authors ([hd.chrisfoulon@gmail.com](mailto:hd.chrisfoulon@gmail.com) or [michel.thiebaut@gmail.com)](mailto:michel.thiebaut@gmail.com)).

We also measured whether the shape of the disconnectome changes over age. We assessed this question by producing disconnectome maps for each decade. We quantified similarities using squared spatial Pearson’s for the 21-30-year-old maps and the maps for the other decades. The result indicates that disconnectome maps show a very high anatomical similarity between decades. Hence disconnectome maps in our sample did not show any age-related changes.


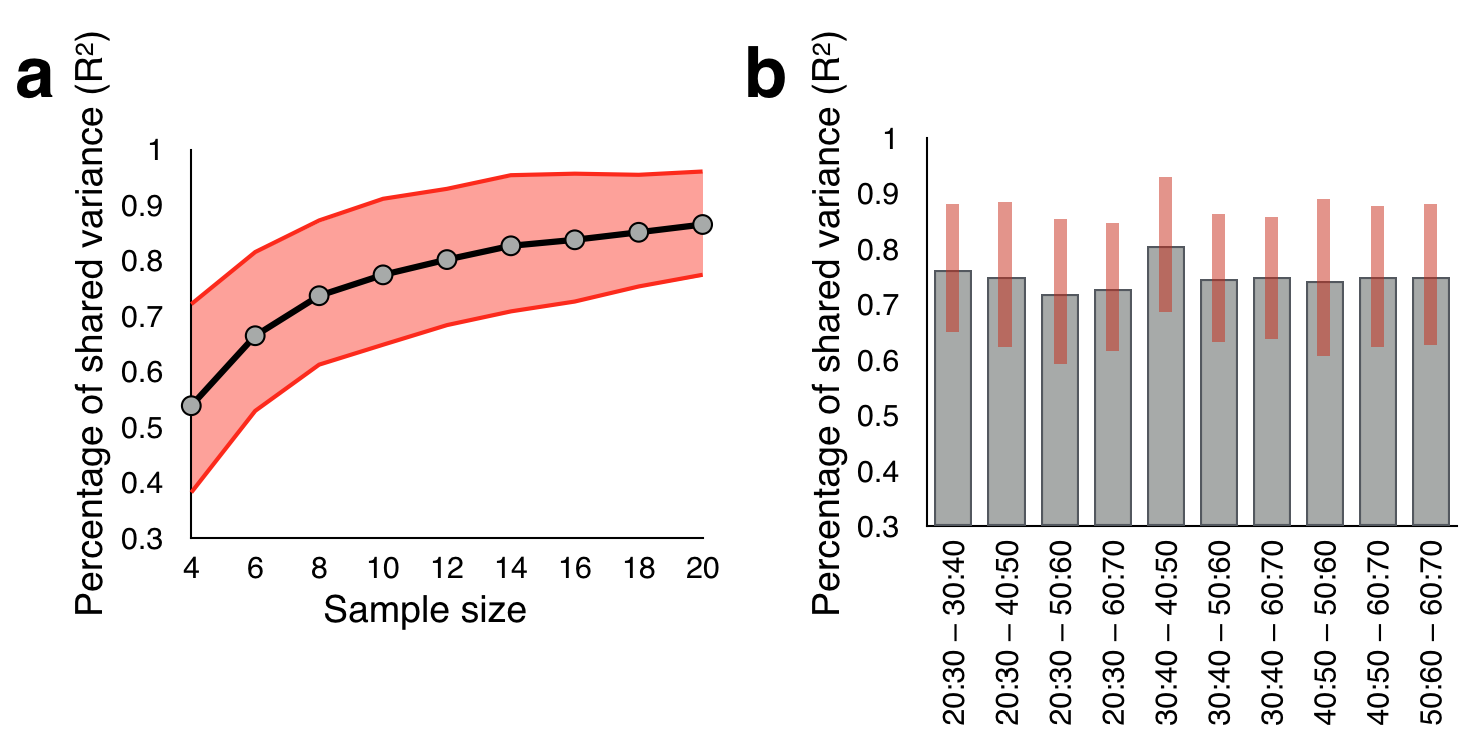


Disconnectome maps optimisation. a) Percentage of shared variance according to sample size. Red areas indicate standard deviations b) Cross-correlation between decades. Red bars indicate standard deviations.
