# Supplementary material for: Advanced lesion symptom mapping analyses and implementation as BCBtoolkit
Source: Gigascience. 2018 Feb 8;7(3):giy004. doi: 10.1093/gigascience/giy004 (PMC5863218; doi:10.1093/gigascience/giy004)
Supplement: Response-to-Reviewer-Comments_Original-Submission.pdf [file giy004_response-to-reviewer-comments_original-submission.pdf]

Author's Response To Reviewer Comments

Changes have been highlighted in blue in the manuscript.

Reviewer #1: In this article Foulon et al present a novel multimodal suite of software for evaluation of focal brain lesions and their associated networks. They demonstrate the utility of their tools using an analysis of 37 patients with frontal lobe lesions and relate lesions to category fluency performance. 54 healthy comparison subjects are also included and all subjects were asked to name as many animals as they could within 60 seconds. 10 of the 54 subjects have DTI imaging.

Subjects had structural T1 images and 10 minutes of resting state data.

Overall I think this is a valuable contribution to the literature and to the lesion mapping community. The main strength in my opinion is the sheer number of analyses performed that all attempt to address various aspects of lesion-associated networks as it relates to category fluency performance. The fact that these analyses can be done by anyone using freely available software packages provided by the authors is important. The authors provide a well-written and well-referenced introductory overview of lesion mapping and diaschisis.

Thank you.

Some of the limitations of the article in its current form include the following:

While the category fluency analysis servers primary to illustrate the novel methods used and is valuable in that regard the sample size for this complex functional task seems underpowered. If the primary goal of the article was to definitively outline the neural basis of category fluency I'm not sure this sample would warrant a high impact journal.

We explored category fluency as a proof of concept. We now acknowledge that a larger dataset should be investigated in the discussion.
'Finally, we applied our methods to the neural basis of category fluency as a proof of concept. The anatomy of category fluency should be, ideally, replicated in a larger sample of patients including lesions involving the entire brain to provide a more comprehensive understanding of category fluency deficit after a brain lesion. While gathering such a large dataset of patients with brain lesions would have been impossible to achieve before, it might soon become possible thanks to collaborative initiatives such as the Enigma Consortium stroke recovery initiative (http://enigma.ini.usc.edu/ongoing/enigma-stroke-recovery/) (Liew et al., 2017).'

While the authors focus on lesion-associated networks it would still be useful to display a standard lesion overlap image and perform some type of VLSM on the lesions themselves. If lesion symptom mapping of the lesions themselves is not significant this doesn't necessarily hinder their conclusions and may actually support the need for lesion-associated network analyses.

Thank you for this suggestion. In the context of our study, classical VLSM did not reveal any significant area involved with category fluency. We added this information in the discussion.

Because there are so many novel analyses the methods section feels inadequate to me. It is difficult to follow exactly what analyses are being performed without reading carefully through the manuscript multiple times.

Apologies if this was unclear, we now produced a figure to help the reader to follow the analyses step by step, reorganised the result section so that it mirrors the method section exactly. We clarified the Anacom section and used a fixed terminology in the methods and results.

"In the following sections of the manuscript, the term clusters systematically refers to the result of the post-hoc Mann-Whitney comparison between disconnected patients and healthy subjects that survived Bonferroni-Holm correction for multiple comparisons."
We also chose a fixed term for the three functional networks produced by the functional connectivity calculation followed by a PCA:
"In the following sections of the manuscript, the term factor-networks systematically refers to brain regions having a statistically significant relationship with the three components."

Whenever possible the authors should be more explicit about what is being used as seed regions for a given analysis (e.g. lesion masks or statistical maps from prior analysis) and when they are using data from controls versus the patients themselves. If space limitation is the problem perhaps a supplemental methods section would help.

We're sorry if this was unclear. As mentioned above we now use a fixed terminology to indicate exactly what is being used for the analyses.

Normalization - Uses a creative method of filling in the damaged tissue by copying the images from the healthy hemisphere opposite the lesion. This helps with the accuracy of registration into MNI space. One limitation of this approach is the requirement of manually tracing the lesion in both the native space and in MNI152 space, but this will be a valuable tool nonetheless.

Indeed, this is one of the limitations, which has now been included in the discussion:
"However, our methods require the manual delineation of lesion masks, automatization remaining a big challenge, especially on T1-images (Liew et al., 2017)."

White matter disconnection -
This tool takes the lesion mask and its overlap with standard regions of interest defined using a white matter atlas (Rojkova, 2016).

I wonder why the authors chose this particular atlas.

The main reason for this choice is that this atlas maps the main white matter tracts of the frontal lobes where are mainly located the lesions of our dataset.

The white matter tracts appear quite large and if I understand the methods the defined the

presence of a tract by a probability of 50% or greater. When doing this for the corticospinal tract as a quality check there are voxels that appear to me to be entirely outside of where the corticospinal tract is.

The corticospinal tract actually originates from different part of the brain and does include motor areas primary somatosensory cortex and premotor areas. The overall extent of the tract of our atlas is comparable to post-mortem sections (Rademacher 2001).
Additionally, we now added the JHU atlas inside the tractotron.

I wonder if the authors could input more than one white matter atlas into their toolbox, such as other freely available atlases (e.g. JHU).

Thank you for the suggestion, we added the JHU inside the Tractotron.

I also think it would be beneficial to use the probability data from the white matter atlas to weight the lesion involvement in the tract. For example if a lesion hits the center of a tract it would carry a greater weight than hitting the periphery. This could be done by using a summation of each voxel's probability from each white matter tract as opposed to thresholding and binarizing the white matter tract.

As such the lesion load for each tract could be treated as a continuous variable and correlated to the behavior of interest.

This is good suggestion as mentioned in the introduction we provide here a simple example of the use of the methods. The neuroscientific community is more than welcome to use this material to explore disconnection differently.

Direct disconnection -
This analysis used the lesion masks as seed regions of interest. Each lesion mask was brought into the native space of 10 healthy subjects that had DTI imaging and tracts were produced. The resulting tracts were thresholded, binarized and statistics were performed to relate the tracts to category fluency using AnaCOM2, a package for lesion symptom mapping.

The authors note they binarized the tracts produced in Trackvis. What threshold was used and what is the justification for choosing it?

Apologies if this was unclear in the manuscript. We didn't use a threshold in trackvis, we binarized the fibre density map to indicate presence/absence of the connection rather than the number of streamlines at the individual level. Later on, the group maps are thresholded at >50% to restrict the analysis to fibres represented in more than half of the healthy control population. >50% correspond to an effect size >0.5, i.e. 50% of the variance explained corresponding to a large effect.
We now clarified the methods accordingly

Indirect disconnection -
The significant clusters resulting from the direct disconnection analysis were used as seed ROIs

for a resting state functional connectivity analysis using the normative fcMRI data at the group level.

How was the median network calculated at the group level?

We clarified this in the methods

"The median network resulting from a seed contains, in each voxel, the median of functional connectivity across all the control subjects. Medians were chosen instead of average as they are less sensitive to outliers and are more representative of the group level data (Kenney 1939). The calculation of the functional connectivity was automatized and made available inside the Funcon tool as part of BCBtoolkit. Medians were calculated using the function fslmaths."

Additional details about this analysis would be helpful. Were the significant 'disconnection' sites from the direct disconnection analysis used to seed fcMRI analyses in the control cohort and then the resulting network strength was tested using the patient data? How was connected versus disconnected status determined in these groups?

Thank you for this remark. We omitted to mention this analysis in the method section. This has now been clarified as follows:

"Additionally, for each patient, we extracted the time course that corresponded to each factor-network. These time courses were subsequently correlated to the rest of the brain so as to extract seed-based factor-networks in each patient. FSLstats was employed to extract the strength of factor-networks functional connectivity and subsequently compare patients according to their disconnection status. Note that a patient disconnected in a factor-network is a patient who has a disconnection in at least one of the cluster that contributed significantly to the factor-network."

Structural Changes
Cortical thickness was assessed across entire networks as defined using the indirect disconnection analysis above.

It is not clear to me that there is any way to relate the cortical thinning to the lesion. For example it is possible that these patients have thinning in association with aging and poor vascular health and these factors contributed to category fluency deficits.
Does thinning at these remote sites relate to lesion size?

In order to control for the effect of aging and lesion size the same analysis was repeated regressing out for these two parameters. We're happy to report that the result remained significant.

'The same analyses were repeated controlling for age and lesion size and confirmed the results for ventral fronto-parietal network seeded from the left MFg (Spearman Rho = .423; $p = .01$) , IPs (Rho = .538; $p = .001$) and left opercularis (Rho = .590 ± 0.341 ; $p < .001$) corresponded to a reduced performance in category fluency (Fig. 5). Additionally, a thinner cortical thickness in the left preSMA functional network (Rho = .439 ; $p = .007$) and a higher rs-fMRI entropy (Rho = –

.420 ± 0.370 ; p = .019) in the mid cingulate gyrus functional network was associated with poorer performance in category fluency'

Shannon entropy as a structural measure could be better described.

Thank you for this. We now describe Shannon entropy more in details in the methods of the manuscript.

'Shannon entropy is an information theory derived measure that estimates signal complexity (Shannon, 1997 ; Gray, 2011). In the context of rs-fMRI, measure of entropy measure the local complexity of the Blood Oxygen Level Dependent (BOLD) signal as a surrogate of the complexity of the spontaneous neuronal activity (Ogawa et al., 1990 ; Biswal et al., 1995). Since cells that fire together wire together (Hebb, 1949), for each grey matter voxel Shannon entropy of rs-fMRI can be considered as a surrogate for the complexity of the connections within this voxel and between this voxel and the rest of the brain. Shannon entropy was extracted from the previously preprocessed rs-fMRI using the following formula: -sum(p*log(p)) where p indicates the probability of the intensity in the voxels (Tononi et al. 1998)'

Other comments:
The order of the methods does not match the results for structural changes and indirect disconnection.

Thanks we changed the order of the results to match.

The authors may consider adding larger datasets for the normative fcMRI and DTI analyses from the freely available human connectome project.

The is a good suggestion, unfortunately our normative datasets are matched for age, education and acquisition parameters. Additionally, we tested our methods in the context of category fluency, which is a language task, while our patients are french, and the HCP does not contain data with the particular test, we cannot use it to extend our dataset.

Reviewer #2: In this manuscript the authors demonstrate impact of a lesion on the functional and structural connections or disconnections in frontal lobe lesion patients using category fluency task. Additionally cortical thickness was used which provide a bridge between the cognitive and behaviour measures and the applied methods are available in an open source toolbox called bcbtoolkit.

Major comments:

1. White matter disconnection- Which two groups were compared using the Kruskal Wallis test is not clear?

Apologies if this was unclear. We now clarified the methods accordingly:

“Thereafter, we used AnaCOM2 available within the BCBtoolkit in order to identify the disconnections that are associated with a given deficit, i.e. connections that are critical for a given function. AnaCOM2 is comparable to AnaCOM (Kinkingnehun et al., 2007) but has been reprogrammed and optimised to work on any Linux or Macintosh operating systems.
Initially, AnaCOM is a cluster-based lesion symptom mapping approach, which identifies clusters of brain lesions that are associated with a given deficit, i.e. the regions that are critical for a given function. In the context of this paper, AnaCOM2 used disconnectome maps instead of lesion masks, to identify cluster of disconnection that are associated with category fluency deficits, i.e. the networks that are critical for a given function. Compared to standard VLSM (Bates et al., 2003), AnaCOM2 regroups voxels with the same distribution of neuropsychological scores into clusters of voxels. Then, for each cluster, AnaCOM2 will perform a Kruskal-Wallis test between patients with a disconnection, patients spared of disconnection and controls. Resulting p-values are Bonferroni-Holm corrected for multiple comparisons. Subsequently significant clusters (p-value < 0.05) are used to perform a post-hoc Mann-Whitney comparison between two subgroups of interest (i.e. disconnected patients and healthy subjects). Post-hoc results are Bonferroni-Holm corrected for multiple comparisons (statistical tests and corrections are computed using R language: R Core Team 2016, https://www.r-project.org).
Patients-controls comparison have been chosen as a first step in order to avoid drastic reduction of statistical power when two or more non-overlapping areas are responsible for patients reduced performance (Kinkingnehun et al., 2007). Non-parametric statistics have been chosen as it is fair to consider that some clusters will not show a Gaussian distribution. AnaCOM2 resulted in a statistical map that reveals, for each cluster, the significance of a deficit in patients undertaking a given task as compared to controls.”

2. The authors have done several different analyses and it is very difficult to follow and does not look like hypothesis driven more exploratory analyses. For the reader would be very useful to have a pipeline figure to illustrate the various analyses steps.

Thank you for mentioning this we now indicate in the methods that all analyses were data drive and provide an outline of the analyses as supplementary figure 1

“The following sections of the manuscript are hypotheses driven and outlined in supplementary figure 1.”

3. Connectome maps based on only 10 normal participants for the DTI and comparing them to n=37 patient cohort is not very trivial? The covariance in the tracts was based comparing present or not present could also be artifacts?

Thank you for pointing at this. We kept the normal participants’ dataset as small as possible to avoid increasing drastically the size of the BCBtoolkit.
10 participants are usually considered as the minimum when it comes to build templates. However, the optimal number of participants has never been assessed in the context of the disconnectome maps. Here we explored this question gathering diffusion data from the 54

healthy participants explored behaviourally in our study. This material has now been added as supplementary material with the manuscript.

"The optimal number of participants was calculated disconnectome maps from separate paired populations of equal gender distribution. This approach was repeated for groups consisting of 4, 6, 8, 10, 12, 14, 16, 18 and 20 subjects. Squared spatial Pearson's correlations between each pair (i.e. square of fslcc from FSL) was employed to calculate the percentage of shared variance (i.e. the similarity). Figure 1a indicates a steep increase of shared variance between disconnectome maps produced from 4 to 10 participants followed by a slower increase from 10 to 20 participants. This result indicates that, using the disconnectome, 10 subjects are sufficient to produce a good enough disconnectome map that matches the overall population (above 70% of shared variance). A larger dataset (n = 36) can be downloaded on our website (http://www.bcblab.com/opendata). Additionally, HCP 7T data (n = 166) have been prepared for the disconnectome and are available on demand to the authors (hd.chrisfoulon@gmail.com or michel.thiebaut@gmail.com)."
We also measured whether the shape of the disconnectome changes over age. We assessed this question by producing disconnectome maps for each decade. We quantified similarities using squared spatial Pearson's for the 21-30-year-old maps and the maps for the other decades. The result indicates that disconnectome maps show a very high anatomical similarity between decades and no decrease of this similarity with age. Hence disconnectome maps in our sample did not show any age-related changes.

4. The category fluency task scores was a simple integer value over the whole paradigm which was then correlated is not clear?

The performance value corresponds to the number of animals the subjects were able to produce in 120 seconds

5. The authors bring in functional connectivity as support for the dysfunctional and disconnected area will impact the indirectly connected areas. Better approach would be to prove this by causal approaches like effective connectivity (direction of the connection) instead of functional connectivity which is only correlation.

The effective connectivity is beyond the scope of this study.

6. Some parts mainly the methods and the results are difficult to follow english can be improved like for ex: in discussion „consequences upon a patients' "

Thank you for pointing at this English mistake. The manuscript has now been read and edited by a native speaker.

Minor comments:

Full forms of acronyms at first mention: What is RR?
Thanks, we now clarified this point in the text.
