# Supplementary material for: Advanced lesion symptom mapping analyses and implementation as BCBtoolkit
Source: Gigascience. 2018 Feb 8;7(3):giy004. doi: 10.1093/gigascience/giy004 (PMC5863218; doi:10.1093/gigascience/giy004)
Supplement: Response-to-Reviewer-Comments_Revision-1.pdf [file giy004_response-to-reviewer-comments_revision-1.pdf]

Author's Response To Reviewer Comments

Dear dr. Zauner,

Thank you for considering our manuscript untitled ‘Advanced lesion symptom mapping analyses and implementation as BCBtoolkit’ for a technical report in Gigascience. Additionally, we wish to mention again that we will be happy to be included in the thematic series 'Brainhack: Open tools for Brain Science'

Concerning your question, I am afraid there is no protected data access scheme. This is because of the wording of the consent. We are sorry we could not find any way around this.

Please find below our point by point response to the minor suggestions of reviewer 1.

We are looking forward to your assessment.

Sincerely,

Michel Thiebaut de Schotten

1) The He article is cited under lesion-driven tractography but I believe this was a fcMRI study.
Actually, He article includes tractography as well as fcMRI. Results are reported in figure 7B of their paper.

2) The authors could clarify the comment about how much variance in fcMRI data is explained by the three network model.
Thanks for mentioning this was unclear. We modified the text accordingly.

3) It's not clear to me why Shannon entropy is included in the 'Structural changes in disconnected regions' section. It is a functional measure and perhaps a separate paragraph is warranted.
Apologies if this is confusing. Although Shannon entropy was derived from functional MRI, it is not a functional measure since it was acquired at rest (= no function) in order to estimate the intrinsic connectivity. We strongly believe it should remain in the structural section of the manuscript. This point is defended in the method section of the manuscript as follows:
“In the context of rs-fMRI, the entropy measures the local complexity of the Blood Oxygen Level Dependent (BOLD) signal as a surrogate of the complexity of the spontaneous neuronal activity [76, 77]. Since “cells that fire together wire together” [78], for each grey matter voxel Shannon entropy of rs-fMRI can be considered as a surrogate for the complexity of the connections within this voxel and between this voxel and the rest of the brain.”

4) Page 21, line 1. I think the authors meant to comment on adequate lesion coverage of the entire brain and not lesions that involve the entire brain.
Thanks for noticing this mistake! This has now been amended in the manuscript.
