# Supplementary material for: Advanced lesion symptom mapping analyses and implementation as BCBtoolkit
Source: Gigascience. 2018 Feb 8;7(3):giy004. doi: 10.1093/gigascience/giy004 (PMC5863218; doi:10.1093/gigascience/giy004)
Supplement: Reviewer-1-Report_Original-Submission -- Aaron Boes — 07 Aug 2017 Reviewed [file giy004_reviewer-1-report_original-submission.pdf]

Reviewer Report

Title: Advanced lesion symptom mapping analyses and implementation as BCBtoolkit

Version: Original Submission **Date:** 07 Aug 2017

Reviewer name: Aaron Boes

Reviewer Comments to Author:

In this article Foulon et al present a novel multimodal suite of software for evaluation of focal brain lesions and their associated networks. They demonstrate the utility of their tools using an analysis of 37 patients with frontal lobe lesions and relate lesions to category fluency performance. 54 healthy comparison subjects are also included and all subjects were asked to name as many animals as they could within 60 seconds. 10 of the 54 subjects have DTI imaging.

Subjects had structural T1 images and 10 minutes of resting state data.

Overall I think this is a valuable contribution to the literature and to the lesion mapping community. The main strength in my opinion is the sheer number of analyses performed that all attempt to address various aspects of lesion-associated networks as it relates to category fluency performance. The fact that these analyses can be done by anyone using freely available software packages provided by the authors is important. The authors provide a well-written and well-referenced introductory overview of lesion mapping and diaschisis.

Some of the limitations of the article in its current form include the following:

While the category fluency analysis servers primary to illustrate the novel methods used and is valuable in that regard the sample size for this complex functional task seems underpowered. If the primary goal of the article was to definitively outline the neural basis of category fluency I'm not sure this sample would warrant a high impact journal.

While the authors focus on lesion-associated networks it would still be useful to display a standard lesion overlap image and perform some type of VLSM on the lesions themselves. If lesion symptom mapping of the lesions themselves is not significant this doesn't necessarily hinder their conclusions and may actually support the need for lesion-associated network analyses.

Because there are so many novel analyses the methods section feels inadequate to me. It is difficult to follow exactly what analyses are being performed without reading carefully through the manuscript multiple times. Whenever possible the authors should be more explicit about what is being used as seed regions for a given analysis (e.g. lesion masks or statistical maps from prior analysis) and when they are using data from controls versus the patients themselves. If space limitation is the problem perhaps a supplemental methods section would help.

Normalization - Uses a creative method of filling in the damaged tissue by copying the images from the healthy hemisphere opposite the lesion. This helps with the accuracy of registration into MNI space. One limitation of this approach is the requirement of manually tracing the lesion in both the native space and in MNI152 space, but this will be a valuable tool nonetheless.

White matter disconnection -
This tool takes the lesion mask and its overlap with standard regions of interest defined using a white matter atlas (Rojkova, 2016).

I wonder why the authors chose this particular atlas. The white matter tracts appear quite large and if I understand the methods the defined the presence of a tract by a probability of 50% or greater. When doing this for the corticospinal tract as a quality check there are voxels that appear to me to be entirely outside of where the corticospinal tract is. I wonder if the authors could input more than one white matter atlas into their toolbox, such as other freely available atlases (e.g. JHU).

I also think it would be beneficial to use the probability data from the white matter atlas to weight the lesion involvement in the tract. For example if a lesion hits the center of a tract it would carry a greater weight than hitting the periphery. This could be done by using a summation of each voxel's probability from each white matter tract as opposed to thresholding and binarizing the white matter tract.

As such the lesion load for each tract could be treated as a continuous variable and correlated to the behavior of interest.

Direct disconnection -
This analysis used the lesion masks as seed regions of interest. Each lesion mask was brought into the native space of 10 healthy subjects that had DTI imaging and tracts were produced. The resulting tracts were thresholded, binarized and statistics were performed to relate the tracts to category fluency using AnaCOM2, a package for lesion symptom mapping.

The authors note they binarized the tracts produced in Trackvis. What threshold was used and what is the justification for choosing it?

fMRI Meta-analyses
The authors used a freely available dataset \ website to evaluate previously published fMRI results relating to 'fluency' and 'category.'

Indirect disconnection -
The significant clusters resulting from the direct disconnection analysis were used as seed ROIs for a resting state functional connectivity analysis using the normative fcMRI data at the group level.

How was the median network calculated at the group level?

Additional details about this analysis would be helpful. Were the significant 'disconnection' sites from the direct disconnection analysis used to seed fcMRI analyses in the control cohort and then the resulting network strength was tested using the patient data? How was connected versus disconnected status determined in these groups?

Structural Changes
Cortical thickness was assessed across entire networks as defined using the indirect disconnection analysis above.

It is not clear to me that there is any way to relate the cortical thinning to the lesion. For example it is possible that these patients have thinning in association with aging and poor vascular health and these factors contributed to category fluency deficits.

Shannon entropy as a structural measure could be better described.

Does thinning at these remote sites relate to lesion size?

Other comments:
The order of the methods does not match the results for structural changes and indirect disconnection.

The authors may consider adding larger datasets for the normative fcMRI and DTI analyses from the feely available human connectome project.

Level of Interest

Please indicate how interesting you found the manuscript: An article of importance in its field

Quality of Written English

Please indicate the quality of language in the manuscript: Acceptable

Declaration of Competing Interests

Please complete a declaration of competing interests, considering the following questions:

- Have you in the past five years received reimbursements, fees, funding, or salary from an organisation that may in any way gain or lose financially from the publication of this manuscript, either now or in the future?

- Do you hold any stocks or shares in an organisation that may in any way gain or lose financially from the publication of this manuscript, either now or in the future?
- Do you hold or are you currently applying for any patents relating to the content of the manuscript?
- Have you received reimbursements, fees, funding, or salary from an organization that holds or has applied for patents relating to the content of the manuscript?
- Do you have any other financial competing interests?
- Do you have any non-financial competing interests in relation to this paper?

If you can answer no to all of the above, write 'I declare that I have no competing interests' below. If your reply is yes to any, please give details below.

I declare that I have no competing interests

I agree to the open peer review policy of the journal. I understand that my name will be included on my report to the authors and, if the manuscript is accepted for publication, my named report including any attachments I upload will be posted on the website along with the authors' responses. I agree for my report to be made available under an Open Access Creative Commons CC-BY license (http://creativecommons.org/licenses/by/4.0/). I understand that any comments which I do not wish to be included in my named report can be included as confidential comments to the editors, which will not be published.

I agree to the open peer review policy of the journal

To further support our reviewers, we have joined with Publons, where you can gain additional credit to further highlight your hard work (see: https://publons.com/journal/530/gigascience). On publication of this paper, your review will be automatically added to Publons, you can then choose whether or not to claim your Publons credit. I understand this statement.

Yes
