# Supplementary material for: Advanced lesion symptom mapping analyses and implementation as BCBtoolkit
Source: Gigascience. 2018 Feb 8;7(3):giy004. doi: 10.1093/gigascience/giy004 (PMC5863218; doi:10.1093/gigascience/giy004)
Supplement: Reviewer-2-Report_Original_Submission -- Muthuraman Muthuraman — 15 Aug 2017 Reviewed [file giy004_reviewer-2-report_original_submission.pdf]

Reviewer Report

Title: Advanced lesion symptom mapping analyses and implementation as BCBtoolkit

Version: Original Submission **Date:** 15 Aug 2017

Reviewer name: Muthuraman Muthuraman

Reviewer Comments to Author:

In this manuscript the authors demonstrate impact of a lesion on the functional and structural connections or disconnections in frontal lobe lesion patients using category fluency task. Additionally cortical thickness was used which provide a bridge between the cognitive and behaviour measures and the applied methods are available in an open source toolbox called bcbtoolkit.

Major comments:

1. White matter disconnection- Which two groups were compared using the Kruskal Wallis test is not clear?
2. The authors have done several different analyses and it is very difficult to follow and does not look like hypothesis driven more exploratory analyses. For the reader would be very useful to have a pipeline figure to illustrate the various analyses steps.
3. Connectome maps based on only 10 normal participants for the DTI and comparing them to n=37 patient cohort is not very trivial? The covariance in the tracts was based comparing present or not present could also be artifacts?
4. The category fluency task scores was a simple integer value over the whole paradigm which was then correlated is not clear?
5. The authors bring in functional connectivity as support for the dysfunctional and disconneted area will impact the indirectly connected areas. Better approach would be to prove this by causal approaches like effective connectivity (direction of the connection) instead of functional connectivity which is only correlation.
6. Some parts mainly the methods and the results are difficult to follow english can be improved like for ex: in discussion „consequences upon a patients' "

Minor comments:

Full forms of acronyms at first mention: What is RR?

Level of Interest

Please indicate how interesting you found the manuscript: An article of importance in its field

Quality of Written English

Please indicate the quality of language in the manuscript: Needs some language corrections before being published

Declaration of Competing Interests

Please complete a declaration of competing interests, considering the following questions:

- Have you in the past five years received reimbursements, fees, funding, or salary from an organisation that may in any way gain or lose financially from the publication of this manuscript, either now or in the future?
- Do you hold any stocks or shares in an organisation that may in any way gain or lose financially from the publication of this manuscript, either now or in the future?
- Do you hold or are you currently applying for any patents relating to the content of the manuscript?
- Have you received reimbursements, fees, funding, or salary from an organization that holds or has applied for patents relating to the content of the manuscript?
- Do you have any other financial competing interests?
- Do you have any non-financial competing interests in relation to this paper?

If you can answer no to all of the above, write 'I declare that I have no competing interests' below. If your reply is yes to any, please give details below.

I declare that I have no competing interests

I agree to the open peer review policy of the journal. I understand that my name will be included on my report to the authors and, if the manuscript is accepted for publication, my named report including any attachments I upload will be posted on the website along with the authors' responses. I agree for my report to be made available under an Open Access Creative Commons CC-BY license (http://creativecommons.org/licenses/by/4.0/). I understand that any comments which I do not wish to be included in my named report can be included as confidential comments to the editors, which will not be published.

I agree to the open peer review policy of the journal

To further support our reviewers, we have joined with Publons, where you can gain additional credit to further highlight your hard work (see: https://publons.com/journal/530/gigascience). On publication of this paper, your review will be automatically added to Publons, you can then choose whether or not to claim your Publons credit. I understand this statement.

Yes
